# Oral and Posters, June 8

**DOI:** 10.3402/ejpt.v4i0.21127

**Published:** 2013-06-04

## XIII ESTSS CONFERENCE: “Trauma and its clinical pathways: PTSD and beyond”, Bologna, June 2013 ORAL, JUNE 8 A. PLENARY HALL

## Morning
*Keynote Address*

### Pediatric medical traumatic stress 8:45–9:45

#### M. A. Landolt University Children's Hospital, Zurich, Switzerland

Each year large numbers of children and adolescents are treated in hospitals for severe injuries or illnesses. A significant portion of these children face adverse and painful medical interventions and procedures which they often cannot completely understand, especially if they are young (e.g., organ transplantation, open-heart surgery, burn treatment, etc.). Previous studies have shown that children treated for accidental injuries and severe illnesses are at risk for developing short- and longer-term trauma disorders, such as post-traumatic stress disorder. Importantly, the findings suggest that fathers and mothers are affected too, sometimes even more than the children themselves. After an overview of the current state of research regarding the prevalence of trauma disorders among different groups of pediatric patients and their parents, a pediatric medical stress model is presented to explain the pathogenetic factors that are involved in the development of trauma-related disorders in ill and injured children. Based on this model, measures of primary, secondary, and tertiary prevention will be discussed. The lecture will end with some thoughts and suggestions for the future development of this field.

## Effects of trauma on families and children
*Invited symposium: Pregnancy and birth in refugee survivors of sexual violence with PTSD. Considerations for treatment*

### Cultural considerations in psychological assessment and treatment for pregnant women with PTSD following trafficking and sexual exploitation 10:00–10:20

#### E. Walsh Traumatic Stress Clinic, Camden and Islington NHS Mental Health Foundation Trust, London, UK

A number of studies have identified complex mental health needs in victims of trafficking, and specifically the high prevalence of PTSD (Zimmerman, 2006) and additional anxiety and mood disorders (Zimmerman, 2006). Statistics on women trafficked to the UK and sexually exploited identify that women are from a wide range of countries (UKHTC, 2012). Many women present to psychological services during pregnancy. They may have experienced traumatic events and hold specific cultural beliefs about their experiences that are outside the knowledge base and training of the therapist (such as traditional medicine rituals or beliefs about gender and responsibility for exploitation). Trauma-focused CBT (Grey, Young, & Holmes, 2002) is a recommended intervention for PTSD. Most studies have focused on using CBT with simple (single-incident) trauma, without a specific focus on cultural issues related to the PTSD. More recent studies have focused on using trauma-focused CBT for refugees (Grey & Young, 2008). This presentation will use clinical case material to illustrate the application of a CBT model for victims of trafficking from different cultures to the therapist, who are pregnant or new mothers, where specific cultural experiences and beliefs played an important role in the development and maintenance of PTSD symptoms.

References

Grey, N., Young, K., & Holmes, E. (2002). Cognitive restructuring within reliving: A treatment for peritraumatic emotional “gotspots” in Posttraumatic Stress Disorder. *Behavioural and Cognitive Psychotherapy*, *30*(01), 37–56.

Grey, N. & Young, K. (2008). Cognitive behaviour therapy for refugees and asylum seekers experiencing traumatic stress symptoms. *Behavioural and Cognitive Psychotherapy, 36*, 3–19.

Zimmerman, C., Hossain, M., Yun, K., Roche, B., Morison, L., & Watts, C. (2006). Stolen smiles: A summary report on the physical and psychological health consequences of women and adolescents trafficked in Europe. London: London School of Hygiene & Tropical Medicine.

### Psychological interventions to prepare and support victims of sexual violence with PTSD for and during labour and birth (10:20–10:40)

#### J. Blumberg Traumatic Stress Clinic, Camden and Islington Mental Health Foundation Trust

This first part of the symposium discusses psychological interventions aimed at preparing and supporting victims of sexual violence with PTSD for and during labour and birth. It is hypothesised that refugee and asylum seeking women who have suffered sexual violence and have PTSD are potentially particularly susceptible to re-traumatisation during labour and birth, as well as to subsequent post-natal depression. This is both because previous experience of sexual violence and aggression and previous psychiatric treatment are thought to be risk factors for the development of post-partum PTSD, as well as because of other variables such as lack of social support and cultural alienation (Polachek et al., 2012; Soet et al., 2003). Other commonly experienced stressors such as insecurity regarding immigration status, lack of access to benefits and lack of access to secure housing also seem likely to raise risks of post partum difficulties. Utilising 2 case studies, this aspect of the symposium will focus on interventions aimed at managing the needs of 2 rape survivors. Firstly, to minimise further traumatisation and triggering of past traumatic memories during labour and birth and secondly to manage risk of the development of post-natal depression. A description of the preparatory psychological work with the women themselves and the training of doulas from a voluntary organisation, Birth Companions, will be discussed, as well as multi-disciplinary work with other professionals who were involved in these women's care both pre- and post-birth. Qualitative feedback from the doulas and from the women themselves will be used to illustrate the utility of these interventions.

References

Polachek, I. S., Huller Harari, L. H., Baum, M., and Strous, R. D. (2012). Postpartum Post Traumatic Stress Disorder symptoms: The uninvited birth companion, *IMAJ*, *14*, 347–353.

Soet, J. E., Brack, G. A. & Dilorio, C. (2003). Prevalence and predictors of women's experience of psychological trauma during childbirth. *Birth*, *30*, 36–46.

### The treatment of PTSD in trafficked women who are pregnant 10:40–11:00

#### K. Robjant Traumatic Stress Service, Clinical Treatment Centre, Maudsley Hospital, London, UK and Vivo International

Women who have been trafficked into Europe have frequently experienced multiple incidents of sexual violence in the context of forced sex work. It is not uncommon for victims of trafficking to have already experienced trauma, loss and attachment difficulties in their own upbringing, which may contribute to their vulnerability prior to being trafficked. As a result of their experiences of multiple traumatic events, PTSD may develop. Trafficked women who have experienced sexual violence and become pregnant may represent a particularly vulnerable group and have unique needs. Through case study examples of clients eventually treated with Narrative Exposure Therapy (Schauer, Elbert & Neuner, 2005), the complex interplay between PTSD in relation to sexual violence and the physical and psychological effects of pregnancy and parenting in this client group will be discussed. The cases will illustrate particular dilemmas arising prior to and during treatment, which include decisions around termination, the impact on bonding and attachment and the prevention of intergenerational transmission of trauma.

Reference

Schauer, M., Neuner, F., & Elbert, T. (2005) Narrative exposure therapy. A short term intervention for traumatic stress disorder after war, terror or torture. Germany: Hogrefe & Huber.

## Impact of trauma on communities
*Symposium: Trauma and the legal process of seeking asylum*

### Psychological evidence and asylum decision making 11:45–12:00

#### J. Herlihy Centre for the Study of Emotion and Law, London, UK

A refugee, as defined by the Geneva Convention, is a person who, “… owing to well-founded fear of being persecuted for reasons of race, religion, nationality, membership of a particular social group or political opinion, is outside the country of his nationality and is unable or, owing to such fear, is unwilling to avail himself of the protection of that country …” (Article 1(2)). People seeking protection as refugees have to present their claim to a receiving state, usually including an account of past experiences—often highly traumatic – in order to establish a “well-founded fear”. In order to assess whether the person's situation meets with the refugee definition, state and judicial decision makers must assess whether (a) the account given fits with information known about the claimed country of origin and (b) whether they believe the account given. This “credibility assessment” risks being highly subjective. We will present studies that suggest that assessments of credibility can and should rely on the best available psychological evidence. One area of interest is the consistency of memory, particularly for traumatic experiences. One of the most common assumptions of decision makers is that when details change in the repeated telling of an account, this suggests that the account is untrue. Preliminary data will be presented from a study of repeated interviews of 69 UK trauma survivors, showing significant inconsistency of recall across different types of memories.

### Non-clinicians’ judgments about asylum seekers’ mental health: how do legal representatives of asylum seekers decide when to request medico-legal reports? 12:00–12:15

#### L. Wilson-Shaw^1^, N. Pistrang^1^ and J. Herlihy^2^

^1^University College London, London, UK; ^2^Centre for the Study of Emotion and Law, London, UK

*Background*: Procedures for determining refugee status across Europe are being speeded up, despite the high prevalence of mental health difficulties among asylum seekers. An assurance given is that ‘‘vulnerable applicants’’ will be identified and excluded from accelerated procedures. Although experts have recommended assessments to be undertaken by experienced clinicians, this is unlikely to happen for political and financial reasons. Understanding how non-clinically qualified personnel perform assessments of mental health issues is timely and crucial. Misrecognition of refugees due to the inappropriate use of accelerated procedures involves the risk of returning the very people who have the right to protection from further persecution. *Objective*: To examine the decision making of immigration lawyers, who are an example of a group of nonclinicians who decide when and whether to refer asylum-seekers for psychiatric assessment. *Method*: Semi-structured interviews were conducted with 12 legal representatives working with people seeking refugee or human rights protection in the United Kingdom. The resultant material was analysed using Framework Analysis. *Results*: Themes clustered around the legal case, the client, the representative and the systems, all with subthemes. A mapping exercise integrated these themes to show how representatives brought together questions of (1) evidential reasons for a report, influenced by their legal, psychological and case law knowledge, and (2) perceived evidence of mental distress, influenced by professional and personal experiences and expectations. *Conclusions*: The legal representatives interviewed were well-informed and trained in psychological issues as well as clearly dedicated to their clients. This helped them to attempt quasi-diagnoses of common mental health problems. They nonetheless demonstrated stereotypical understanding of post-traumatic stress disorder and other possible diagnoses and the role of subjectivity. The study has implications for other groups particularly those less trained and compassionate who are required to make clinical judgments without the necessary expertise.

### The importance of looking credible: the impact of the behavioural sequelae of post-traumatic stress disorder on the credibility of asylum-seekers 12:15–12:30

#### H. Rogers^1^, S. Fox^1^ and J. Herlihy^2^

^1^Royal Holloway, University of London, London, UK; ^2^Centre for the Study of Emotion and Law, London, UK

Memory difficulties following traumatic experiences have been found to result in testimonial inconsistencies, which can affect credibility judgements in asylum decisions. No investigations have looked into how/whether the behavioural sequelae of Post-Traumatic Stress Disorder [PTSD] impact decisions. This study aimed to investigate this by looking at whether observable symptoms of PTSD can be confused with perceived cues to deception. An actor performed four versions of a fictional ‘asylum interview’ that contained differing levels of pre-defined ‘deception’ and ‘trauma’ behaviours. Four groups of students (total n=118) each watched a different interview. They gave subjective ratings of credibility, plus quantitative and qualitative information about the factors that influenced their judgements. Despite the content of the interviews remaining the same, significant differences in credibility ratings were found between interviews; with the interview containing both ‘trauma’ and ‘deception’ behaviours being rated as significantly less credible than the interview containing only the PTSD behaviours. ‘Emotional congruence’ was conceptualised as an important factor in influencing credibility. Results are discussed in terms of possible heuristics involved in judgements of an asylum-seeker population, as well as implications for vulnerable asylum seekers whose symptoms do not conform to stereotypes. Limitations and avenues for future research are highlighted.

### Overgeneral memory in asylum seekers and refugees 12:30–12:45

#### B. Graham^1^, J. Herlihy^2^ and C. Brewin^1^

^1^University College London, London, UK; ^2^Centre for the Study of Emotion and Law, London, UK

Studies in Western samples have shown that post-traumatic stress disorder (PTSD) and depression are associated with overgeneral autobiographical memory retrieval. This study assesses whether this association extends to asylum seekers and refugees from diverse cultural backgrounds. The inclusion of specific details in personal testimony has been taken as a marker of credibility in legal guidance. An association could therefore have important practical implications for those providing testimony of their experiences when seeking asylum. 38 asylum seekers and refugees were recruited through clinics and community groups. Clinical interviews assessed PTSD and depression and participants completed a test of autobiographical memory specificity. When accounting for omissions, participants with PTSD and depression recalled a lower proportion of specific memories. Those with PTSD also failed more frequently to report any memory. This study indicated that lower memory specificity observed in people experiencing PTSD and depression in Western populations extends to asylum seekers and refugees from diverse cultural backgrounds. It adds to the literature suggesting that being recognised as a refugee fleeing persecution is more difficult for those with post-traumatic symptoms and depression. Limitations and future directions for research are discussed.

## Afternoon
*Keynote Address*

### Genetic and environmental correlates of trauma-related attachment patterns in children and adults 14:00–15:00

#### M. van IJzendoorn University of Leiden, The Netherlands

Disorganized attachment behaviors and unresolved attachment representations are key-concepts of attachment theory addressing issues of loss or other traumatic experiences across the life span.In this talk these concepts are defined against the background of more common psychiatric syndromes, such as reactive attachment disorder and post-traumatic stress disorder. Recent work on the (epi-)genetics and neurobiology of disorganized and unresolved attachment will be discussed, and environmental influences on the emergence and development of these attachments will be highlighted. Evidence for the effectiveness of interventions on disorganized or unresolved attachments is still scarce.

## Responding to disasters
*Symposium: American Red Cross disaster mental health response lessons learned from hurricane Sandy and the Sandy Hook school shooting*

### American Red Cross disaster mental health response to Hurricane Sandy and the Connecticut shooting: the View from National Headquarters 15:15–15:30

#### V. Cole American Red Cross National Headquarters, Washington, DC, USA

This presentation will describe the structure of the disaster mental health program and how it is mobilized in large disasters. The presenter will focus on the American Red Cross disaster mental health philosophy as it applied to events as diverse as Hurricane Sandy and a school shooting in a small town in Connecticut. In both cases, the community mental health needs differed markedly from what is addressed by typical mental health interventions or community-based psychosocial support programs. The American Red Cross three-element approach emphasizes tailoring services to the needs of the disaster-affected community and spans the disaster cycle from preparedness to response to recovery. Although the majority of the population is expected to be psychologically resilient, recent research shows that 30–40% of those directly exposed to the disaster will develop long-term psychological consequences such as post-traumatic stress disorder, anxiety disorder or major depression. DMH uses psychological triage and mental health surveillance tools to track trends and assist in the allocation of limited resources. Another important function of disaster mental health is to monitor and alleviate stress faced by other disaster responders. Both of these disasters posed significant challenges to staff assigned to the job. Strategies to protect the emotional health of the workforce will be discussed during this presentation as well.

### American Red Cross Response to Hurricane Sandy 15:30–15:45

#### D. Ryan American Red Cross, Washington, DC, USA

Hurricane Sandy made landfall on the New Jersey coast on October 29 with sustained winds of 80 mph. This major storm impacted an area the size of Europe and affected 11 states, the District of Columbia, and Puerto Rico. More than 16,000 Red Cross disaster responders were assigned to the relief operation, serving over 10 million meals and snacks and providing 107,000 health and mental health contacts. This presentation will discuss early mental health interventions such as psychological first aid and crisis intervention in shelters, hotels, home visits, community gatherings, on feeding trucks, and through neighborhood canvassing, along with particular considerations for communities with frail elderly and non-English-speaking populations. The Red Cross model of coordinated efforts between workers in disaster mental health, client case work, and health services will be examined, and use of the PsySTART behavioral surveillance tool will be described. The American Red Cross has a strong staff mental health program, and this presentation will include the distinct needs of staff working through the phases of this hardship relief operation, from the initial sheltering in place in Red Cross offices to assessing the impact of damage to their own homes, through the effects of a prolonged response with exposure to extensive suffering, and with special considerations during the holiday season. While the hurricane relief effort was ongoing, the Red Cross in Greater New York also responded to fatal fires, the Newtown shooting, and a ferry accident with 80+ injuries which required additional mental health response for clients and staff. A discussion of lessons learned will include the importance of pre-disaster partnerships with local, city, and state agencies and NGO's and the critical need for a staff mental health component on hardship disasters.

### Mental health role in death notifications Following the Shootings at Sandy Hook Elementary School 15:45–16:00

#### V. Cole^1^, D. Ryan^2^, W. Dailey^3^ and J. Halpern^4^

^1^American Red Cross National Headquarters, Washington, DC, USA; ^2^American Red Cross in Greater New York, NY, USA; ^3^Yale University School of Medicine, New Haven, CT, USA; ^4^Institute for Disaster Mental Health, State University of New York, New Paltz, NY, USA

In the United States when an unexpected death occurs outside the home in a non-institutional setting, a police officer or staff member from the coroner's office normally informs the family or significant others as soon as possible in a face to face meeting. The simplicity and straightforwardness of this approach reduces the likelihood that the victim's family will first learn of the death from another, perhaps less reliable, source, such as news media. While officials who perform death notifications attempt to do so in a compassionate and respectful manner, most have little, if any, training regarding the psychological consequences of traumatic events, or how to promote recovery among those who mourn an untimely death. Nevertheless, awareness of the need to improve death notifications has been growing. In the aftermath of the school shootings in Sandy Hook, Connecticut, officials decided to include mental health professionals and clergy members as part of three-person teams that then visited families of victims. While the efficacy of this approach has not been scientifically evaluated, anecdotal evidence suggests it helped comfort families of those killed and connected them at the outset with mental health and spiritual resources designed to promote subsequent recovery. In addition to describing the importance of involving mental health professionals in death notifications, this presentation will describe: (1) How and why mental health personnel became involved in notifications at Sandy Hook; (2) How mental health professionals might assist government officials in planning for and responding to mass casualty events; and (3) How to support self-care among mental health responders involved in death notifications and mass casualty events.

### Crisis counselling for survivors of the Sandy Hook Shooting 16:00–16:15

#### J. Halpern Institute for Disaster Mental Health, State University of New York, New Paltz, NY, USA

This presentation will discuss the mental health interventions used to support families, community members, and first responders in the first week after the Sandy Hook school shooting. Examples of “working the Maslow hierarchy” of basic needs will be presented. Counselors addressed basic safety needs as well as issues of meaning. Instrumental support was helpful to some survivors, who needed proper clothes or travel expenses for funerals. Counselors addressed safety needs as families were exposed to new threats as well as sights and sounds that triggered intense reactions. Families also needed protection from intrusive press, celebrities, onlookers and unwelcome acquaintances. Members of this religious community also benefitted from talking about meaning and religion. Counselors reinforced existing social support networks of trusted family, friends, and clergy and provided informational support or psychoeducation as parents sought counsel on how to help their surviving children. Parents’ many challenging questions such as “how to explain these events to young children” will be presented. Case examples demonstrating the importance of reassurance, safety, routine and honesty, will also be discussed. The clinical usefulness of the “Dual Process Model of Coping with Bereavement” will be reviewed as family members struggled not only with loss but with having to make difficult decisions such as whether or not they should stay or leave the community. The counseling that was delivered to surviving members of the school community will also be described. The challenge of providing an orderly mental health response when not all clergy or mental health providers were sufficiently trained or screened will be reviewed.

## Responding to disasters
*Symposium: Trauma informed practice; Lessons learned from the juridical and school system when interacting with surviving youth from the Norwegian massacre of July 22nd 2011*

### Police Interviews with Terror Exposed Youths - Experiences from police interviews after the Terror Attack in Norway, 2011 16:45–17:00

#### A. Langballe, J. Schultz and T. Wentzel-Larsen Norwegian Centre for Violence and Traumatic Stress Studies, Oslo, Norway

The objective of this study is to explore how the affected youths have experienced the conduct of the police interviews four months after the terror attack in Oslo. 281 youth responded to both closed and open ended questions about their experiences from the police interviews. They were asked to describe their emotions and reactions during and after the police interview, and to what extent they experienced this as a stressful and meaningful activity. The police interview could be characterized as a potentially secondary traumatizing event for the victim. Usually the young victim has no experience of the legal system which often appears to be totally out of his/ hers control. However, only 10% of the youth considered the interview as partly or extremely stressful situation. Through the victim's narratives the paper will discuss both factors that have decreased feelings of helplessness and stress and increased the positive experiences from the interviews, and how the youth at the same time felt the interview mentally and psychologically demanding. The findings will be discussed according to knowledge of principles based on traumatic stress exposure and forensic psychology.

### The Trial after the Terror Attack in Norway, 2011 - Terror Exposed Youth’ Experiences from the Trial Process 17:00–17:15

#### S. Laugerud and A. Langballe Norwegian Centre for Violence and Traumatic Stress Studies, Norway

The objective of this study is to explore how terror-exposed youth experienced the trial after the terror attack in Norway in 2011. A year after the attack the perpetrator was sentenced to jail. Approximately 2–4 months after the trial, a total of 290 (N=320) survivors were interviewed as a part of a longitudinal study measuring exposure, PTSD, and other indicators of mental health. The survivors were asked to describe how they experienced the trial and how it affected their emotional well-being. Among them 40 survivors testified in court and they were asked to give ample descriptions of how they experienced retelling and reliving the traumatic event in court. During the trial the survivors were exposed to the perpetrator and repeatedly exposed to pictures and descriptions of the traumatic event. Within Victimology the legal system has been characterized as a high-risk environment for victims with potential for “revictimization” and “retraumatization”. At the same time a trial may provide restoration of trust, safety, relationships, and contribute to repairing social, emotional and psychological harm. In the presentation preliminary findings will be discussed according to knowledge of the Norwegian legal system and the theoretical perspective of “revictimization” and “retraumatization”.

### Returning to School after Surviving the Norwegian Terror Attack in 2011 - Students Self Perceived Capacity for Learning after Being Terror Exposed 17:15–17:30

#### J. Schultz, A. Langballe, T. Wentzel-Larsen and G. Dyb Norwegian Centre for Violence and traumatic Stress Studies, Oslo, Norway

The objective of this quantitative study is to explore the relationship between trauma-related symptoms and school achievement among Norwegian terror-exposed youth present at the Utøya terrorist attack in 2011. A total of 220 adolescents participating in the study (N=320) were enrolled in school; ranging from lower and upper secondary school and higher education. They were answering a comprehensive questionnaire 4 months after the massacre. Exposure, post traumatic stress symptoms and indicators of mental health were collected in addition to indicators of school adjustment, school attendance, coping and school achievement. Students perceived capacity for learning 4 months after the attack is analyzed in relation to PTSD-symptoms. There will also be a presentation of the youths’ report of to what extent and in what way the schools have adjusted and adapted the student's learning environment in the first months after the massacre.

### Teachers’ Perceptions of Meeting their Terror Exposed Students - The Teacher Role in Facilitating and Adapting the Learning Process 17:30–17:45

#### M. Dalset^1^ and J. Schultz^2^

^1^Department of Special Needs Education, Faculty of Educational Sciences, University of Oslo; ^2^Norwegian Centre for Violence and Traumatic Stress Studies; Oslo, Norway

In the aftermath of the 22 July 2011 massacre in Norway, the Minister of Education and Research urged teachers and school leaders to go to the greatest possible length to adjust the learning environment of students that were directly affected by the terror. The objective of this study is to explore how teachers and school leaders perceive their role in terms of supporting students that were present during the Utøya terror attack. Using a qualitative design, the study explores feelings of uncertainty and confidence related to the follow-up of these students. 24 teachers and 12 school leaders from 6 schools with a total number of 36 exposed students were selected for the study. Preliminary findings indicate that the perceived role of teachers is unclear when it comes to which actions that should be taken in school settings. The informants seem to possess different levels of trauma knowledge and skills. Consequently, they report varying degrees of uncertainty when it comes to ways of transforming general trauma-related information into individually appropriate action; ensuring a flexible practice to accommodate student needs within a rigid school system; balancing demands and consideration; helping traumatized students achieve better educational outcomes; and talking with students about challenging topics. A broad spectrum of measures has been implemented, ranging from minimal actions on the one hand to extensive personal involvement on the other. According to prevailing theory, neither of these extremes is considered beneficial. So far limited research has been conducted on how trauma-informed practices in schools can help mitigate or intensify the impact of traumatic experience.

## ORAL, JUNE 8 B. PLENARY HALL

## Morning Impact of trauma on communities
*Panel: Human rights violations, trauma and the role of the ESTSSM*

### Human Rights violations, trauma and the role of the ESTSS 8:45–10:00

#### F. Orengo^1^, D. Adjukovic^2^, B. Lueger-Schuster^3^ and J. Den Otter^4^

^1^Spanish Society of Psychotraumatology (Sepet), Madrid, Spain; ^2^Faculty of Psychology, Zagreb, Croatia; ^3^Faculty of Psychology, Vienna; ^4^International Rehabilitation Center For Victims of Torture, Copenhagen, Denmark

The ESTSS is now committed to go a step ahead in the defense of HR, which is no less as the prevention of psychotraumatic disorders. We want to reflect on how the ESTSS can be much more active in HR issues. We want to create spaces, places or gathering and been together offering professional advice and experience but also the opportunity to sit and discuss the best therapeutic practices. The ESTSS wants to be a forum for those psychotraumatologists of different countries or social groups that are or were in conflict. We want to give them the opportunity, as the most skilled European scientific society in the field of trauma, to find a place of peace and communication, of respect and warm interchange.

**Presenters**:

Dean Adjukovic will discuss the longer-term consequences of traumatization in violent conflicts for communities and societies. Brigitte Lueger Schuster will present some suggestions about how the ESTSS may use its leading role in psychotraumatology to foster exchange about human rights issues by providing a frame for the gathering of research questions, sharing of experiences in treatment, and initiating of preventive measures, that is empowerment through human rights. Joost den Otter will comment on the work of the International Rehabilitation Center for Victims of Torture. Francisco Orengo will present on the positive and negative effects of the so- called “Ley de la memoria histórica”, a Spanish Law from 2004 that tried to repair / treat the legal and social problems related to the long term effects of the Spanish Civil war.

## The spectrum of trauma-related disorders
*Symposium: Sexual abuse and violence in the Catholic Church – research and practice on disclosure and the latent impact on psychopathology*

### Testimonials of victims of sexual abuse in the Roman Catholic Church—comparison of data collected by the victim hotline of the Roman Catholic Church in Germany and the contact point of the German Independent Commissioner 10:00–10:15

#### M. Rassenhofer^1^ and A. Zimmer^2^

^1^Department of Child and Adolescent Psychiatry/Psychotherapy, University of Ulm, Ulm, Germany; ^2^Diocese Trier, ZB 1.3.2 Counceling Services, Germany

*Background*: As reaction to the “German abuse scandal” in 2010, caused by the disclosure of former cases of child sexual abuse in some Catholic and pedagogical institutions, the Roman Catholic Church in Germany as well as the Independent Commissioner, assigned by the German Government, established telephonic contact points for victims of sexual abuse. The hotline of the Catholic Church included internet counseling by psychological experts. The contact point of the Independent Commissioner aimed at collecting information for the political process. Burdened callers were referred to counseling services. *Objective and method*: Both contact points were accompanied by research processes, information, and experiences callers transferred in conversations were documented and analyzed. This presentation focuses on description and comparison of testimonials of victims in the context of the Roman Catholic Church given to the two contact points. The two samples are compared concerning demographic aspects as well as characteristics and dynamics of abuse. Furthermore, psychosocial consequences that were reported by the victims are presented and contrasted. *Results*: From the victim hotline of the Roman Catholic Church resulted N=753, from the contact point of the Independent Commissioner N=413 analyzable data sets of victims who reported sexual abuse within a Catholic context. While callers of the Catholic hotline were predominantly Catholics or former members of the Roman Catholic Church (95%), this group only represents a relatively small part of the population of victims that addressed themselves to the contact point of the Independent Commissioner (9%). Testimonials given to the two contact points are relatively similar. Callers were mostly middle-aged and mainly informed about repeated abuse in the past by a male offender. Often victims reported even several psychosocial problems as consequences of the abuse. *Discussion*: Testimonials of victims deliver further insight into the dynamics of sexual abuse. Prevention and intervention strategies can be derived.

### Victim protection in Austria—an overview of the work of the Independent Victims Protection Commission (UOK) 10:15–10:30

#### U. Konrad Independent Victim Protection Commission, Austria

In 2010 the “Independent Victims Protection Commission (UOK)” was established after reports of violence and sexual abuse in institutions of the Catholic Church against children shocked the public. In this presentation, I will report on the structure and tasks of the UOK. The main objective of the UOK is to collect and administer reports of these cases (“clearing”). It provides legal and psychological support for the survivors. Other important tasks are the active and preventive protection of victims accompanied by campaigns to raise awareness and inform the public. The UOK is formed by professionals from different fields (law, psychiatry and psychology, education, and media) and is led by the former governor of Styria. In total, the UOK registered and archived 1,439 allegations and was able to process 1;221 allegations. From 2010 until the end of December 2012, the UOK completed 904 cases. Survivors contacted the UOK from all Austrian regions The UOK developed guidelines for compensation: after the first assessment interview at the UOK, the clearing phase is initiated with up to 10 hours of interviews and supportive counseling with a psychologist. The victims are able to choose freely the psychologist they want to work with. More than 100 psychologists, specialized in trauma psychology, work with the UOK. Furthermore, there are also lawyers available to the survivors for legal advice and procuration. After the clearing phase, the survivors are offered further psychological treatment paid by the Catholic Church. Also financial compensation between 5,000 and 25,000 Euros are given. Since 2010, more than 23,500 hours of psychological treatment have been provided and more than 8 million Euros have been paid to the survivors. The guidelines and the procedure of case-management as well as the individual process management will be presented. Recommendations for further commission work will be discussed.

### Survivors of institutional abuse committed by the Austrian Catholic Church—a study on the posttraumatic outcome and prevalence of abusive acts 10:30–10:45

#### B. Lueger-Schuster^1^, D. Weindl^2^, V. Kantor^2^, R. Jagsch^2^, Y. Moy^2^, A. Butollo^2^ and M. Knefel^2^

^1^Clinical Unit, Institute of Applied Psychology, University of Vienna, Vienna, Austria; ^2^Faculty of Psychology, Unit of Clinical Psychology, University of Vienna, Vienna, Austria

*Background*: Since the 1990s, Austrian survivors of institutional abuse (IA) have been demanding acknowledgment and criminal investigations. In April 2010, an Independent Survivors’ Protection Commission was established to redress and support the survivors. This study analyzed the data of 450 survivors of IA, who disclosed to the commission. *Objectives and Methods*: The prevalence of IA committed by clerical professional workers and the abuse-related disorders were analyzed. Different kinds of data collection were used. Four-hundred fifty (age *M=*55 years, range 25–80) survivors gave written informed consent to scientifically analyze their clearing documents. These documents comprised written reports (passive participation), including psychological assessment given by mental health professionals. Of these 450 survivors, 185 completed self-report questionnaires (BSI, PCL-C, and instruments measuring resilience). *Results*: We present results from the analysis of the clearing documents and the questionnaire data. IA was experienced with an average age of 10 years. 75% of the sample was men. From the 185 who filled in the questionnaires almost 50% suffered from PTSD. 82% of the 185 reported intrusions and 71.6% reported a clinical relevant psychopathological symptom distress. More boys than girls suffered from childhood sexual abuse, whereas girls were more exposed to acts of violence. However, the prevalence of PTSD is higher in the females. Those with less PTSD symptoms were more optimistic and resilient. No differences were found in demographic factors and the numbers and types of exposure. *Conclusion*: IA always leaves a mark on the survivors. The results shed light on the complex dynamics of IA and its consequences on psychopathological outcome. Further research on the complex relationship of IA and its psychological consequences is needed to enhance the development of specialized treatments.

### “What if …”—survivors personal views on the impact of institutional abuse in Catholic Church in later life 10:45–11:00

#### V. Kantor^1^, D. Weindl^2^, M. Knefel^2^, Y. Moy^2^, A. Butollo^2^, R. Jagsch^2^ and B. Lueger-Schuster^2^

^1^Clinical Unit, Institute of Applied Psychology, University of Vienna, Vienna, Austria; ^2^Faculty of Psychology, Unit of Clinical Psychology, University of Vienna, Vienna, Austria

*Background*: In 2010, numerous adult survivors of institutional abuse (IA) committed by members of the Austrian Catholic Church disclosed their experiences to a specifically established victim protection commission. In cooperation with this commission a research project at the University of Vienna assessed the survivors with standardized questionnaires. Although these instruments provided interesting results, a deeper insight into the very personal consequences of IA was needed because previous research paid no attention to these issues. The primary aim of this study was to understand survivors’ perceptions of the effects of IA on their later life. Qualitative research based on phenomenography was carried out to explore survivors’ explanatory approach toward their experiences. *Method*: Specially trained clinical psychologists conducted 47 semi structured in-depth interviews with 39 male and 8 female survivors of IA (Age M=58.66, range: 38–80). All interviews were tape-recorded and transliterated verbatim. For in-depth analysis, quantitative content analysis (Mayring, 2010) was used. The main interview features were: (1) personality before/after the IA; (2) “what if” the IA had not had happened; (3) feelings of shame and guilt; (4) breaks in life according to IA. *Results*: All participants experienced physical, emotional, and/or sexual violence by members of the Catholic Church. Almost all participants described personality changes related to the IA. They also reported small to large consequences of the IA on their lives’ path (e.g., effects on interpersonal relationships, career, etc.). Feelings of shame and guilt were especially prevalent in cases of sexual violence. *Conclusion*: These constructs resulting from the analysis of narratives are discussed in the light of recent findings. Our results contribute to a better understanding of the effects of IA and have important implications for psychotherapy and clinical work.

Reference

Mayring, P. (2010). Qualitative Inhaltsanalyse. Grundlagen und Techniken [Qualitative Content Analysis] (11th ed.). Basel: Beltz Verlag.

## Miscellaneous
*Symposium: Trauma assessment 2.0: using customized computer apps in clinical research and praxis*

### A generic questionnaire framework supporting psychological studies with smartphone technologies 11:45–12:00

#### J. Schobel^1^, M. Ruf-Leuschner^2^, R. Pryss^1^, M. Reichert^1^, M. Schickler^1^, M. Schauer^2^, R. Weierstall^3^, D. Isele^3^, C. Nandi^3^ and T. Elbert^2^

^1^Institute of Databases and Information Systems, University of Ulm, Ulm, Germany; ^2^Department of Psychology, University of Konstanz & vivo international, Konstanz, Germany; ^3^Department of Psychology, University of Konstanz, Konstanz, Germany

Many psychological studies are performed with specifically tailored “paper & pencil”-questionnaires. Such a paper-based approach usually results in a massive workload for evaluating and analyzing the collected data afterwards, e.g., to transfer data to electronic worksheets or any statistics software. To relieve researchers from such manual tasks and to improve the efficiency of data collection processes, we realized smart device applications for existing psychological questionnaires (e.g., the KINDEX, PDS, or CAPS questionnaire). Based on these applications, we were able to demonstrate the usefulness of smart devices (e.g., smartphones or tablets) for mobile data collection in the context of psychological questionnaires. Although the implemented applications already have shown several advantages in respect to data collection and analysis, they have not been suitable for psychological studies in the large scale yet, e.g., due to the high maintenance efforts for the psychologists. More precisely, changes to a questionnaire or its structure still must be accomplished by computer scientists, since its implementation is hard-coded. What is needed instead is an easy-to-use and self-explaining framework for creating, running, and evolving the questionnaires of psychological studies on mobile and smart devices. In this context, supporting the complete questionnaire lifecycle is essential, i.e., IT support for creating, using, evaluating, and archiving questionnaires is required to assist end-users having no programming background. We present our generic questionnaire framework, which encompasses the following three parts: a questionnaire configurator to create the questions and questionnaires, a way of integrating mobile devices to deploy, run and log questionnaires, and a middleware enabling a secure data exchange. Finally, we discuss how smartphone technology and mobile devices can be used to suitably support psychologists in their daily work with questionnaires. As major benefit of the framework, better data quality, shorter evaluation cycles, and significant decreases in workload will result.

### Detecting adverse childhood experiences with a little help from tablet computers 12:00–12:15

#### D. Isele^1^, M. Ruf-Leuschner^2^, R. Pryss^3^, M. Schauer^2^, M. Reichert^3^, J. Schobel^3^, A. Schindler^3^ and T. Elbert^2^

^1^Department of Psychology, University of Konstanz, Konstanz, Germany; ^2^Department of Psychology, University of Konstanz & vivo international, Konstanz, Germany; ^3^Institute of Databases and Information Systems, University of Ulm, Ulm, Germany

Adverse childhood experiences, ranging from abuse to emotional neglect, damage the mental and physical health and may impede the treatment of mental disorders. However, validated instruments that assess childhood adversity including the full range of childhood maltreatment are lacking. The adverse childhood experiences index (ACE; Dube et al., 2003; Felitti et al., 1998) retrospectively assessed different forms of abuse, neglect, and household dysfunctioning during the first 18 years of life, and quantified the “breadth of the experienced adversities”, by means of the ACE score. Thus, this instrument allows quantifying the magnitude or “dose” of toxic childhood experiences. A recent modification of the ACE index, by Teicher and colleagues (2011, MACE Scale), gathers in even greater detailed and in more comprehensive ways information about the various types of maltreatment: self experienced abuse or neglect, as well as peer victimization and witnessing domestic violence are all explored in detail. Supplementary information gained about emotional reactions to the events, and temporal anchoring of the experienced, are highly valuable for psychotherapeutic and research purpose. We present short versions of the MACE and a pediatric version (Isele et al., in prep.), adjusted to the cognitive and emotional development status of minors. These new versions fill the need for structured clinical interviews, mapping abuse, and neglect in this sample. Their application in clinical research and therapeutic contexts is shown including an electronic tablet-computer supported assessment.

### Preventing further trauma: KINDEX mum screen—assessing and reacting towards psychosocial risk factors in pregnant women with the help of smartphone technologies 12:15–12:30

#### M. Ruf-Leuschner^1^, R. Pryss^2^, M. Liebrecht^2^, J. Schobel^2^, A. Spyridou^1^, M. Reichert^2^ and M. Schauer^1^

^1^Department of Psychology, University of Konstanz & vivo international, Konstanz, Germany; ^2^Institute of Databases and Information Systems, University of Ulm, Ulm, Germany

The KINDEX mum screen has been designed to be administered by gynecologists and midwives during pregnancy for the assessment of the main psychosocial developmental risk factors, which include traumatic experiences of the parents, intimate partner violence, drug abuse, a history of mental health problems, poverty, acute stress, and others. In addition, we have developed a self-assessment version that runs on tablet computers (iPads). Validation of the KINDEX has been successfully completed in Germany, Spain, Greece, and Peru. Gynecologists or midwives interviewed 80–120 pregnant women in each country. A randomized sub sample of respondents was assessed by trained clinical psychologists using standardized structural interviews to assess perceived stress and mental disorders. 14-months after giving birth the new mothers were interviewed again and the predictive value of the KINDEX was assessed by structured clinical interviews and the analysis of the cortisol levels (deposited in hair over a month) of mother and child as indicator for stress. The results show that the KINDEX assesses valid information about existing risk factors through a structured 15-minute interview with the pregnant women or through the application of this instrument as self-rating on a tablet computer. The tablet computer application in addition to the paper–pencil version has the advantage of automatic analysis of the data and instant recommendation for further support of the pregnant woman.

### Screening for mental disorders in post-conflict regions using computer apps—a feasibility study from Burundi 12:30–12:45

#### A. Crombach^1^, C. Nandi^1^, M. Bambonye^2^, M. Liebrecht^3^, R. Pryss^3^, M. Reichert^3^, T. Elbert^4^ and R. Weierstall^1^

^1^Department of Psychology, University of Konstanz, Konstanz, Germany; ^2^Université Lumière de Bujumbura, Bujumbura, Burundi; ^3^Institute of Databases and Information Systems, University of Ulm, Ulm, Germany; ^4^Department of Psychology, University of Konstanz & vivo international, Konstanz, Germany

A high level of psychosocial functioning is essential for survival in many resource-poor countries and is needed for development in these regions. Organized violence, often in combination with other stressors such as poverty and familial conflict, however, result in a range of mental disorders and damage socio-economic progress. An efficient assessment of mental health is a prerequisite for prevention and intervention measures. However, this may require considerable resources that are difficult to obtain in resource-poor countries. We present new methods for the efficient and effective assessment of mental, especially trauma- and stress-related disorders that can easily be administered by trained local paramedics. For decades, Burundi has been a staging ground for armed conflicts leaving behind many survivors with trauma-related illness. In a study with over 900 combatants and veterans from the military as well as former rebels in Burundi, we used a tablet-computer (ipad)-based survey for the assessment of trauma-related syndromes, especially PTSD, in need for treatment. All participants reported the experience of serious traumatic stressors and a substantial portion presented severe symptoms of the trauma-spectrum. Based on the PSS-I and other standardized screening instruments, an ipad app guided the semi-structured clinical interviews. Psychologists from the University of Konstanz, the Burundian military as well as psychologist students from the University Lumière, Bujumbura, Burundi carried out the interviews. In this contribution, we use the Burundian example to portray the logistics and technology of data acquisition and present respective data. We demonstrate the feasibility of using a computer-based screening approach in the field and in clinical settings. We provide evidence, that the computerized assessment of clinical symptoms can be a useful tool for mental health assessment and screenings, both in research and practice.

## Afternoon
*Keynote Address*

### Difficult pathways to childbirth: post-partum PTSD and its implications for mother and child 14:00–15:00

#### P. Di Blasio Research Center on Developmental and Educational Dynamics (CRIdee), Dipartimento di Psicologia, Universitá Cattolica del Sacro Cuore, Milano, Italy

Studies of childbirth-related PTSD gained significant attention after the DSM-IV (APA, 1994) revisions. Such revisions introduced the concept of “stressful situations in which a person had experienced, witnessed, or was confronted with an event that involved actual or threatened death or serious injury, or a threat to the physical integrity of self or others” replacing the assumption of “extreme events that were outside the range of usual human experience” (DSM-III, 1980). Research, then, started to explore childbirth as a potential in connection with both negative and traumatic experiences related to this event, such as stillbirth, pregnancy loss, premature birth, perinatal death. Furthermore, research has also started to uncover how childbirth-related negative experiences may interconnect with a spectrum of risk factors for posttraumatic stress symptoms, such as a history of pre-existing psychological problems, anxiety, a negative cognitive appraisal of the delivery, negative contacts with the medical staff, obstetric variables, being a single parent, lack of partner support, a difficult pregnancy, previous sexual abuse, or other types of previous traumatic experiences. More recently, the scientific community has put forward the idea that women may also develop posttraumatic stress disorder (PTSD) even in cases of a regular childbirth involving a full-term pregnancy and a healthy child and mother. In other words, the typical childbirth may also be experienced as such, a emotionally charged event that it can lead to the development of posttraumatic stress symptoms or even to a full diagnosis of PTSD. Childbirth is a complex experience, which differs from other traumatic events because it is associated with a range of positive and negative emotions. Childbirth elicits psychological and physiological adjustments; also, it is usually positively perceived by the partner and family even when mothers-to-be are worried about their physical integrity and their child's health. Worries are also about uncertainties concerning their ability to adapt competently to the new requirements of the child and to the social context. Prevalence rates of childbirth-related symptoms satisfying full criteria for PTSD are between 1.5 and 5.6% and prevalence rates of threshold PTSD are between 24 and 33%. In a few cases, PTSD symptoms co-occur with post-natal depression. Posttraumatic symptoms together with comorbidities may have a considerable impact on the mother's ability to process information about the child, frequently leading to a negative perception and representation of the child. In fact, women who present PTSD-related emotional numbing may be at higher risk of parenting problems due to a lack of emotional responsiveness to the child's behavior, and to a lack of communication and attuned interaction with the child. The PTSD-related hyper arousal may, in turn, elicit maternal irritability and anxiety toward the child. It is warranted that health professionals support mothers to elaborate the stress mechanisms of post-partum PTSD—even in typical childbirths—to avoid any emotional overloading of women who are already experiencing a spectrum of stressful tasks to welcome the new born.

## Effects of trauma on families and children
*Symposium: Prevention and treatment of trauma in children and adolescents new directions and special challenges*

### Intergenerational transmission of trauma and posttraumatic stress disorder in an epidemiologic sample 15:15–15:30

#### A. L. Roberts^1^, S. Galea^2^, S. B. Austin^1^, M. Cerda^1^, R. J. Wright^3^, J. W. Rich-Edwards^4^ and K. C. Koenen^2^

^1^Harvard School of Public Health, Boston, USA; ^2^Columbia University, New York, USA; ^3^Harvard Medical School, Boston, USA; ^4^Brigham and Women's Hospital, Boston, USA

*Background*: Research conducted using small samples of persons exposed to extreme stressors has documented an association between parental and offspring posttraumatic stress disorder (PTSD), but it is unknown whether this association exists in the general population and whether trauma exposure mediates this association. We sought to determine whether mothers’ posttraumatic stress symptoms were associated with PTSD in their young adult children and whether this association was mediated by higher trauma exposure in children of women with PTSD. *Methods*: Using data from a cohort of mothers (*n*=6924) and a cohort of their children (*n*=8453), we calculated risk ratios (RR) for child's PTSD and examined mediation by trauma exposure. *Results*: Mother's lifetime posttraumatic stress symptoms were associated with child's PTSD in dose-response fashion (mother's 1–3 symptoms, child's RR=1.2; mother's 4–5 symptoms, RR=1.3; mother's 6–7 symptoms, RR=1.6, compared to children of mothers with no symptoms, *p*<0.001 for each). Mother's lifetime symptoms were also associated with child's trauma exposure in dose-response fashion. Elevated exposure to trauma substantially mediated elevated risk for PTSD in children of women with symptoms (mediation proportion, 74%, *p*<0.001). *Conclusions*: Intergenerational association of PTSD is clearly present in a large population-based sample. Children of women who had PTSD were more likely than children of women without PTSD to experience traumatic events; this suggests, in part, why the disorder is associated across generations. Health care providers who treat mothers with PTSD should be aware of the higher risk for trauma exposure and PTSD in their children.

### Reducing the impact of child trauma exposure: reaching children and parents through trauma-informed systems and online 15:30–15:45

#### N. Kassam-Adams Children's Hospital of Philadelphia, Philadelphia, USA

How can we reduce the impact of acute trauma for children and parents? Mental health professionals play an important role in addressing the impact of child trauma, but many children and families exposed to trauma will not seek mental health services, especially in the acute aftermath of trauma exposure. Thus, a key challenge to our field is to reach beyond traditional mental health service models to prevent the development of persistent and troubling traumatic stress responses and other psychological sequellae of acute trauma in children. This presentation will describe two areas of research and program development that are reaching beyond mental health services to address the impact of child trauma exposure: 1) promoting trauma-informed practices within systems that reach many children proximal to the time of trauma exposure (e.g., health care, schools, law enforcement), and 2) developing online and e-Health approaches that can have wide reach to children and families. Mental health professionals have opportunities to help develop and support trauma-informed services in other systems. Taking health care systems as an example, the presentation will provide an update on current work to promote trauma-informed practice by pediatric health care providers, and to develop and evaluate practical methods for screening and secondary prevention in the health care setting. We will also describe e-Health approaches in this area, including innovative web-based tools for parents, children, and health care professionals. The cutting edge of current research and practice in this area is systematic examination of which interventions work, for whom, during which time period post-trauma, and how these can be integrated into the health care delivery context. In the area of e-Health, rigorous development and evaluation are underway, next steps include attention to dissemination of resources so that parents and children have timely access to online resources after trauma exposure.

### Challenges in the application and dissemination of TF-CBT in community settings 15:45–16:00

#### A. Mannarino Allegheny General Hospital, Pittsburgh, USA

Trauma-focused cognitive-behavioral therapy (TF-CBT) has been extensively studied in 13 randomized clinical trials and demonstrated to be effective in reducing children's trauma symptoms and behavior problems and parents’ emotional distress and depressive symptoms. Nonetheless, there are always challenges in transporting an evidence-based treatment (EBT) from an academic, research setting to frontline community agencies. This presentation will focus on some of these challenges and also provide some ideas as to how to overcome them. One major challenge is that clinicians often do not feel a great deal of administrative support when implementing TF-CBT and over time, begin to use the model less frequently and/or consistently. Accordingly, it is essential to educate administrators and supervisors about trauma impact and the clinical benefits to families of using TF-CBT so that they can support their therapists both in terms of obtaining the necessary training and supervision and implementing the model with fidelity. Another challenge occurs when organizations are implementing more than one EBT at the same time. In these situations, there can be some tendency toward “therapist drift” as clinicians may integrate different therapeutic models or incorporate some aspects of one EBT into a different EBT. Ongoing supervision and consultation is critical to assist therapists in determining with what cases TF-CBT is appropriate and how to implement the model with fidelity. Other challenges include the additional financial costs of implementing an EBT such as TF-CBT, how to sustain the model at a given agency when TF-CBT-trained clinicians leave the organization, and how to encourage clinicians and administrators to use objective assessment measures to evaluate treatment progress.

### Prevalence and risk factors of PTSD in children and adolescents after the 2012 earthquake in the Emilia Romagna region: implications for intervention 16:00–16:15

#### B. Forresi, C. Del Giovane, F. Soncini, G. Aggazzotti, R. d'Amico, E. Parmelli, E. Righi and E. Caffo University of Modena and Reggio Emilia, Modena, Italy

PTSD is one of the psychological disorders that occurs after natural disasters. Many cases will remit within a few months, however in some estimates nearly one-third of cases have a chronic course. Delay-onset PTSD and progressive increase of symptoms seem to be very common. Given the significant rates of PTSD among children and adolescents after earthquakes and the long-term impact on their mental health, it is of primary importance to identify and treat symptoms effectively. The authors will present preliminary data from a cross-sectional study aimed at evaluating the prevalence of PTSD in a sample of children and adolescents nine months after the 2012 earthquake that hit the Emilia Romagna region in northern Italy. Data concerning risk (e.g., level of trauma exposure and parental psychopathology) and protective factors for the development and the persistence of the disorder will be also presented. Children and adolescents (age range: 9–14 years), randomly selected from schools in the Province of Modena, have been assessed using an exposure questionnaire on objective/subjective experiences during the earthquake, the UCLA PTSD index for DSM-IV (UPID), and the strengths and difficulties questionnaire (SDQ). Parental symptomatology has been also assessed, in order to evaluate the influence of parental psychopathology on offspring's adjustment. Given the few studies conducted in Italy about the long-term psychological impact of natural disaster on children and adolescents, the present research has important implications for the prevention and treatment of traumatized children and adolescents in Italy, as well as for the development of effective posttrauma interventions.

## Vivo Invited Symposium: Psychological consequences and needs of survivors of organized violence

### Mental health needs of survivors of acute armed conflict and consequences of family and community violence 16:45–17:15

#### U. Karunakara Medecins sans Frontieres, Geneva, Switzerland

Organizations working in regions where armed conflict takes place are confronted with huge challenges, e.g. providing food, shelter, security and medical assistance to large groups of refugees. During the last 15 years, these organizations have learned that it is necessary to expand their focus and also include the mental health consequences into their assistance. More recently, many organizations had to realize that domestic and community violence are additional problems in refugee settlements with multiple consequences. The presentation will present multiple case examples and a general analysis and will discuss consequences for international crisis intervention and humanitarian aid.

### Mental health of asylum seekers and refugees in Italy: an epidemiological study 17:15–17:30

#### E. Danese Vivo International, Italy

Asylum seekers and refugees usually have experienced a large number of traumatic and stressful events, such as torture, imprisonment, direct exposure to the war, as well as physical and sexual violence. Considering these dramatic rates of trauma it is not surprising that asylum seekers and refugees are considered at high risk to develop post migration mental health problems. In fact, there is consistent evidence showing that compared to the general population these populations have much higher rates of psychiatric disorders, in particular Posttraumatic Stress Disorder (PTSD) and depression. In Italy, the situation of asylum seekers and refugees is particularly difficult because of the lack of studies assessing their psychological wellbeing. The present epidemiological study was conducted to estimate the magnitude and type of mental health problems related to traumatic stress in a sample of 125 asylum seekers and refugees (mean age=28; 78.4% men, 21.6% women) living in Northern Italy. Clinical psychologists carried out structured interviews including standardized measures for the assessment of exposure to traumatic events, for the diagnosis of PTSD as well as the severity of depression and suicidal ideation. More than 80% of the participants have experienced torture; a similar percentage has been directly exposed to the war. As a consequence, 30% of the sample was diagnosed with (mostly severe) PTSD and 25% with Depression. 15% of the participants reported suicidal thoughts and 70% had complaints about somatic problems. A clear dose-effect relationship was found between exposure to various traumatic stressors and PTSD; those who had been exposed to a large number of traumatic events had a higher risk for developing psychological problems. The results support findings regarding the mental health of asylum seekers and refugees from other countries and point to a rather alarming situation in Italy. More research in this field is needed, most urgently to develop and evaluate adequate types of intervention.

### Life stories of asylum seekers in Italy: experiences from a medical care project 17:30–17:45

#### A. Nava Medecins sans Frontieres, Rome, Italy

In the center of this presentation will be the exemplary life stories of two refugees out of 350 who came to Italy and were followed up on their way in the host country. In the center of MSF's work in Italy is a holistic approach that does not reduce the refugees problems to the DSM definition of traumatic stress and PTSD but includes the idea of the refugee him-/herself about his/her project of life. This includes that their life back in their country of origin was characterized by suffering, losses and violence, that they decided to leave their original social context (often collecting debts) and that they started a long journey facing dangerous situations and humiliation before finally reaching Italy. They gradually approach the new context in Europe which is at first imagined and unknown, which later became unfamiliar and finally obscure, due to the impossibility and denial of the right to reach what they had in their mind and what they had hoped for hoped for. When disillusioned and deeply involved into a total lack of ‘normality’, they start to vent different physical and mental symptoms and they are obliged to spend the last energy for a new struggle for survival. What means at this point to start a path of integration or rehabilitation, looking towards which ‘destination’? Is the work on their story essential to start any kind of integration? Which kind of resilience we are going to raise? Is their ‘trauma’ the result of what is behind them or is still waiting for them?

### War at home - consequences of organized violence for family life 17:45–18:00

#### C. Catani Vivo International and University of Bielefeld, Germany

A substantial body of research shows the detrimental effects of war on the mental health of the civilian population. One of the most vulnerable groups in this context are children. Data from various studies including vivo's work in different conflict zones around the world range from a 20% to 50% prevalence of posttraumatic stress disorder (PTSD) in children and adolescents even years after the traumatic war experience. In addition, many children in these contexts suffer from comorbid affective and somatic problems. Apart from these direct or immediate effects of war exposure, the development of children in (post-) conflict areas is threatened by a wide range of secondary factors, such as homelessness, malnutrition, loss of a parent, or family violence. Whereas the direct consequences of organized violence have been addressed by a large number of studies, only very few have considered the indirect and multifaceted effects of war on family life and on the social and economic conditions that affect the family. One important finding from this new line of research is that war, together with its concomitants, seems to make families particularly vulnerable to an increased perpetration of violence especially towards their children. This presentation will introduce and discuss these findings including recent studies conducted by our workgroup with families in post-war countries Northern Sri Lanka and Northern Uganda. Besides pointing to the well-known harmful consequences of parental violence on the healthy development of the affected children, our data also show that positive and supportive parenting can buffer the negative effect of war on mental health in children. The presentation will end by addressing implications of these findings for the treatment of war-traumatized families and the prevention of further use of adverse parenting strategies and violence towards children.

## ORAL, JUNE 8 HALL AUDREY GRACE

## Morning
*Symposium: Traumatic parenthoods and perinatal depression: adaptation and psychopathology in stressful conditions*

### The Family wound 10:00–10:15

#### R. de Bernart ITFF (Istituto di Terapia Familiare Firenze), Florence, Italy

From the beginning of Systemic Family Therapy, we found a connection between the symptom presented by the identified patient and his/her function in a delicate balance of family relationships. This function often (if not every time) was responsible of stopping the growth of the person affected, because this was not compatible with continuing the function for the family. More recently working on the meaning of the symptom we found that it was always connected with a *family wound*, which took place often in a previous generation and which was the cause of the need of the function. In many cases, a perinatal problem (like for instance the death of a parent or a serious problems in the quality of life of the family, or other traumatic events) was behind this wound. The author will try to introduce this theoretical concept trough some clinical examples possibly with images and videos.

### Attachment, adaptation and psychopathology in perinatal period: the father’s role 10:15–10:30

#### F. Baldoni Attachment Assessment Lab, Department of Psychology, University of Bologna, Bologna, Italy

In the perinatal period, fathers may suffer from affective disorders similar to post-partum depression with a frequency ranging in the world from 2% to 31.3%, with a mean of 10.4% in 2010 (Paulson, Bazemore, 2010, Baldoni, Ceccarelli, 2010). The clinical expression of *Paternal Perinatal Depression* (PPND) differs from maternal perinatal depression. In these cases, the depressive symptoms are less severe, less definite, and often occur in comorbidity with other disorders whose symptoms could overlap with the affective one causing complicated clinical pictures. In particular anxious disorders, illness behavior alteration (in particular somatically focused) and behavioral acting outs (aggressiveness, alcoholism, addiction disorders) are frequent. Moreover, in the perinatal period the mother's and father's emotional states are linked and empirical research has found a significant correlation between PPD and MPD. In fact, anxious or depressed fathers, or those with behavioral problems, can be a handicap for the emotional equilibrium of their companion and for the good development of the relationship between mother and child. A lack of their “secure base” protective function can foster an affective disorder in the mother and negatively influence the attachment and psychomotor development of the child. Some research data that confirm this hypothesis will be presented, in particular:

1. Fathers whose companions have undergone affective post-partum disorders show anxiety, depressive symptoms, irritability, somatic complaints and worry about their own health and paternal role up to the fifth month of pregnancy (Baldoni, Baldaro, Benassi, 2009);
2. During in vitro fertilization and embryo transfer procedure (IVF-ET), when the male is anxious, depressed or hostile, women manifest more severe affective disorders, anxiety and somatization independently of the success of the procedure (Baldoni, et al. 2010);
3. Depression, low dyadic sensitivity and insecure attachment forerunners in fathers influence the development of preterm born children (Baldoni, et al. 2012).

### Prevalence and course of maternal postnatal depression following preterm birth 10:30–10:45

#### F. Agostini^1^, F. Monti^1^, E. Neri^1^ and A. Biasini^2^

^1^Department of Psychology, University of Bologna, Bologna, Italy; ^2^U.O. Intensive Neonatal Care, Bufalini Hospital, Cesena, Italy

*Introduction*: Prematurity is a very traumatic experience for both infant and his/her parents. Important consequences regard the infant's survival and the risks for long-term developmental, cognitive, emotional and behavioral problems; at the same time, the premature birth is associated to parents’ worries, fears, concerns and negative affective states relative to the survival of the baby. Perinatal depression has been detected in about 50% of preterm babies’ mothers, a percentage which is higher than the one found in the general population (Brandon et al., 2011). At 6 months of corrected age, these levels tend to decrease to 20% and remain stable up to 2 years (Miles et al., 2007; Poehlmann et al., 2012), representing a relevant risk for the development of the preterm child, up to 3 years (Singer et al., 1999). *Objectives and methods*: The project aims at evaluating prevalence and course of postnatal depressive symptoms and comorbid anxious symptomatology in preterm babies’ mothers at relevant moments of infant development: 3, 9 and 12 months of infant's corrected age. Analyses will be carried on comparing three groups of mothers differentiated by: 1) Preterm delivery of a baby weighing less than 1500 grams (Very Low Birth Weight); 2) Preterm delivery of a baby weighing less than 1000 grams (Extremely Low Birth Weight); 3) Term delivery of a healthy baby. At every step of the project, mothers will complete the Edinburgh Postnatal Depression Scale (Cox et al., 1987), Parenting-Stress Index (Abidin, 1995), Penn State Worry Questionnaire (Meyer et al. 1990), Social interaction and Anxiety Scale (Mattick, Clarke, 1998). Besides, the level of child development will be assessed using the Griffiths Mental Development Scales (Griffiths, 1996).

### The relationship between attachment style, affect dysregulation and perinatal depression 10:45–11:00

#### G. Craparo^1^, C. Crisafi^2^, N. Ragonese^2^ and V. Caretti^3^

^1^Faculty of Human and Social Sciences, Kore University of Enna, Enna, Italy; ^2^Department of Psychology, University of Palermo, Palermo, Italy; ^3^Università La Sapienza, Rome, Italy

*Introduction*: Many authors considers maternal post-partum depression as a syndrome related to depression during pregnancy. This study aims to explore the relationship between attachment style, alexithymia (a specific difficulty to perceive and communicate to others emotional states characterized by affective dysregulation) and perinatal depression (prenatal and post-partum)*. Methods*: Fifty-nine women with an age ranged from 16 years to 40 years (*M*=28.05; *SD*=5.85) at 5th–8th months of pregnancy completed the following self-report questionnaires: *Beck Depression Inventory—II* (BDI-II: Beck, Steer, Brown, (1996); *Toronto Alexithymia Scale—20 items* (TAS-20: Bagby, Parker, Taylor, 1994; Bagby, Taylor, Parker,1994); *Attachment Style Questionnaire* (ASQ: Feeney, Noller, Hanrahan, 1994). At 2–4 weeks postpartum the following self-report questionnaires were administered: *Beck Depression Inventory - II* (BDI-II: Beck, Steer, Brown, (1996); *Postpartum Depression Screening Scale* (PDSS: Beck, Gable, 2000); *Attachment Style Interview* **(**ASI: Bifulco, et al., 2002). *Results and conclusions*: Analysis of data evidenced a significant correlation between alexithymia, insecure attachment and perinatal depression (prenatal and post-partum). In particular, this study suggests that an insecure attachment style and the presence during pregnancy of alexithymic traits accompanied by affective dysregulation and depression symptoms seem to be important risk factors of post-partum depression.

## Symposium: Early identification and early treatment of the psychopathological consequences of torture

### The extreme trauma and torture survivors identification interview (E.T.S.I.): preliminary outcome of a clinical trial in Cameroon and Chad 11:45–12:05

#### L. Mosca and M. Germani San Giovanni Hospital, Rome, Italy

The *E.T.S.I. Interview* was developed by the Centre for post-traumatic and stress pathologies of San Giovanni Hospital in Rome. It was designed as a semi-structured clinical interview for the early identification of the clinical consequences of extreme trauma and torture in asylum seekers. It comprises five different sections, exploring: 1) Trauma related symptoms; 2) Previous traumatic experiences; 3) Resilience; 4) Interviewer's clinical evaluation; 5) Background information. A reliable and prompt identification of survivors of extreme traumas is a rather pressing need to maximize the possibility of a successful treatment. Indeed, it enables the clinician to proceed with early referrals to specialized centers, where they can receive the most appropriate treatments. On the contrary, delayed screening and diagnosis will result in inappropriate treatments, higher levels of individual suffering, possible social consequences, in addition to higher expenses for the public health system and social services. The *E.T.S.I. Interview* also provides a specific Triage evaluation tool, to determine, through a specific color code, the likelihood a person has experienced extreme traumas and the urgency for medical and psychological interventions. A first clinical and statistical trial project was conducted in 8 first reception centers in Italy, which showed the reliability of the *E.T.S.I. Interview*, as well as the high correlation rates between its sections. A second clinical and statistical trial project is being carried out in Chad and Cameroon, in cooperation with the Trauma Centre Cameroon and the Association Jeunesse pour la Paix et la Non Violence in Chad. Preliminary results will be presented.

### Early interventions in the treatment and rehabilitation of torture and extreme trauma survivors 12:05–12:25

#### M. Germani and M. Luci San Giovanni Hospital, Rome, Italy

Due to the severity and the specificities of the psychopathological consequences of complex traumas, an effective treatment requires the integration of different therapeutic interventions either at the same time, or consecutively. The clinical experience acquired in specialized centers for the treatment of torture survivors, in accordance with the new therapeutic approaches and techniques defined by the most recent scientific literature, has identified three main stages within the treatment of individuals surviving extreme traumas. These, can be defined as: 1) Early phase or stabilization and symptoms reduction; 2) Integration of traumatic memories; 3) Integration of the personality, development of relational and metacognitive skills. In the specific case of refugees surviving torture, the role of social instability and marginality, their emotional “desert”, the uncertainties regarding their future, the loss of the previous cultural, social and familiar identities, increase the burden of the severe psychopathological wounds produced by the experiences of violence and torture. These issues add uncertainties and hindrances to the clinical evolution of torture survivors to overcome their severe post-traumatic impasse. Hence, the natural history of the complex psychopathological conditions in these patients moves towards exacerbation and chronicity, if no adequate treatment is implemented. The early phase of the treatment is, therefore, crucial. The possibility to have access to different, integrated multidisciplinary interventions, conducted by experienced professionals, should be considered as a conditio sine qua non in the treatment of complex traumas in refugees, who survived torture. Moreover, supporting teams must include medical doctors, psychologists, social workers, legal professionals and trainers. In this field, different approaches, which are generally not considered therapeutic interventions, as social holding, legal assistance, psycho-social rehabilitations, are a relevant part of the treatment. This multidisciplinary approach only can generate a sufficient therapeutic compliance, the possibility of a positive therapeutic relationship and the achievement of a symptomatic stabilization. In this presentation, the different specific interventions of the early phase, will be discussed: pharmacological and psychopharmacological treatments, supportive psychotherapeutic treatments, psychoeducation, etc.

### From theory to clinical practice: the case of Mr. Kole 12:25–12:45

#### C. Pagani, M. Curia and C. Ruffetta Centro di Consultazione Etnopsichiatrica di Milano, Italy

The clinical case in question regards a young man who fled the Ivory Coast, leaving his wife and small baby. He arrived in Italy seeking asylum in July 2011. The huge sense of humiliation and shame induced by profound memories prevented him from preparing a claim for the Refugee Commission's audition. His credibility was at risk because of inconsistencies in his accounts and confusion. In June 2012 the System Protection for Asylum Seekers (S.P.R.A.R.) referred Kole to the Centre of Etnopsychiatry for his memory problems. A week before his first appointment at our Centre, the patient had a bad accident on the first day of job-training, breaking his leg. The dissociation symptoms caused by the intrusive nature of his memories had been undervalued. The psychotherapy revealed that Kole was in a state of “un-reflectiveness”, overwhelmed by disruptive feelings due to a complex grief and the depression for the loss of his family members. The patient felt pangs of remorse and cried for several weeks before being able to confess persecution and violence perpetrated over generations to his family, accused of anthropophagy. The treatment had to face the huge feeling of guilt and the interiorization of the internal persecutor. History and psychology were both necessary to shed light on the historical, political and psychological mechanisms of torture. In addition to the treatment, we prepared a psychological certification for the Refugee Commission that allowed Kole to self-disclose 2 months later on the day of the interview and be recognized as a refugee. The therapy supported the patient in progressively assuming personal responsibility for his future, adjusting the distorted perception of the self. This clinical case demonstrates the importance of reestablishing the truth and justice for the healing process.

## Afternoon
*Debate: Towards a trauma-informed listening - strategies for journalists and clinicians across different domains*

### Towards a trauma-informed listening: strategies for journalists and clinicians across different domains 16:45–17:45

#### Convenors: E. Newman^1^, G. Rees^2^ and V. Ardino^3^

^1^University of Tulsa, USA; ^2^Dart Centre for Journalism and Trauma, London, UK; ^3^PSSRU Unit, London School of Economics and Political Science, London, UK

Good listening is an essential skill for both journalists and clinicians, especially those working with trauma survivors. But, what do we mean by good, trauma-informed listening? Do different modes of listening have different implications for the accuracy of the interview or assessment and for the wellbeing of the person being listened to? How does power need to be understood in trauma-informed listening strategies? This workshop aims to explore three different understandings of what might constitute good listening. Rees will look at the interview encounter from the perspective of journalists interrogating powerful political and business figures, Newman will explore the challenges of working with those in a structurally powerless situation and Ardino will discuss the challenges of the forensic interview, where the subject may simultaneously be both victim and perpetrator, and where malingering may be an issue. The workshop will also offer an overview of strategies for training mental health professions and journalists.

## ORAL, JUNE 8 HALL DIAMANTE

## Morning
*Open Papers: Response to disasters*

### The Six-C's Model—guidelines for the emergency mental health providers 10:00–10:15

#### M. Farchi Department of Stress, Trauma & Resilience Studies, Tel-Hai College, Israel

The Six-C's Model—Guidelines for the Emergency Mental Health Providers

The SIX C's model was created for addressing the need to standardize the mental health interventions during Acute Stress Reaction (ASR). The model is based on 4 theoretical models:

**Hardiness** (Kobasa, 1979; Maddi, 2006): A combination of three attitudes (commitment, control, and challenge) that together provide the courage and motivation needed to turn stressful circumstances from potential calamities into opportunities for personal growth.

**Sense of Coherence** (Antonovsky, 1979): A model that describes the personal dispositions serve to make individuals more resilient to the stressors they encounter in daily life.

**Self-Efficacy** (Bandura, 1988): The belief of one's ability to succeed in specific situations and challenges.‘

**Psychoneuroimmunology** (**PNI**): The relation between the limbic system and the prefrontal cortex during stressful events (Gidron, 2001).

These models are the platform for the Six C's Model aimed to *shift the person from a helplessness state to a coping survivor*. This goal can be achieved using the six following elements:

**Cognitive Communication**: Using cognitive channel for verbal communication.

**Challenge**: Activation through physical and cognitive challenges.

**Control**: Activation with encouragement of the ability to choose from deferent options

**Commitment**: Verbal commitment to once safety.

**Continuity**: Restructuring once memory into logical chronological sequence.

This model was successfully implemented over 250 traumatized persons during the latest Israeli operation in Gaza.

The presentation will include demonstration of the model using case studies & Videos. Implementations for more targeted population (as ER patients) will be discussed.

### Recovery in psychological distress and self-rated health in survivors of a natural disaster—a longitudinal comparison with a population sample 10:15–10:30

#### L. Wahlstrom, H. Michélsen, A. Schulman and M. Backheden Karolinska Institutet, Stockholm, Sweden

Knowledge of the course of psychological health in survivors of disasters are often hampered by the lack of access to predisaster data or relevant comparison groups. We had the opportunity to compare longitudinal data from survivors of the 2004 Indian Ocean tsunami with a matched population sample. A total of 831 survivors from Stockholm were categorized according to the combination of different types of exposure (severe injury, life threat, loss, presence on the beach/in water), and compared on measures of Self Rated Health and General Health Questionnaire-12, with a matched population sample of 3322 individuals from the same region. Data were collected in three waves; 14 months, 3 years, and 6 years postdisaster from survivors, and in the same year as the third wave, from controls. Analyses with ordinal data were performed with logistic regression for proportional odds, and with control for a number of sociodemographic variables. In comparison to the population sample, for the more exposed groups, levels of psychological distress were still higher at 3 years, and had with few exceptions levelled out at 6 years. In data from the sixth year post-disaster, survivors of most exposure groups reported higher levels of Self Rated Health, than the matched population sample. We will discuss the use of the Self Rated Health measure, and the General Health Questionnaire, and the mechanisms behind this paradoxical benefit, in the context of disaster.

### Conceptualizing events outside of “normal human experience” in trauma victims following a disaster 10:45–11:00

#### A. Ahmad University College London, London, UK

Psychologically fragmented individuals, suffering the effects and consequences of a disaster, creates certain challenges for the responding medical practitioner. How may such trauma be understood? Norris et al. (2002) reviewed literature on the psychosocial consequences of a disaster, concluding that over 1/3 of studies described individuals who suffered from severe distress including diagnosable disorders. An ontological distinction will be made between a trauma that occurs (in)ternal to an individual and a trauma that occurs (ex)ternal to the individual. Natural disasters are unlike other forms of trauma because the individual can suffer from the destruction of property, and the disintegration of their lifestyle and livelihood. In this paper, I refer to phenomenological aspects, which are apparent during a rupture of the land. For example, my analysis focuses on how the individual's rupture of experience can accommodate an ownership of their being-in-the-world (their territory) and an understanding of their altered narrative (their landscape). The paper leads onto discuss an individual's psychological symptoms as part of a symbiotic relationship with their land; the other. This allows for a re-framing of the individual's situation as a transformative narrative rather than a pathology. Finally, I explore the implications of my argument for cross-culturally applying a PTSD diagnosis.

### Applications of psychological first aid around the world 11:00–11:15

#### L. Snider^1^, M. Van Ommeren^2^ and A. Schafer^3^

^1^War Trauma Foundation, Diemen, The Netherlands; ^2^Department of Mental Health and Substance Abuse, World Health Organization, Geneva; ^3^World Vision International, Melbourne, Australia

Psychological first aid (PFA) has been recommended by many international expert groups, including the Inter-Agency Standing Committee, the Sphere Project and World Health Organization's (WHO) mhGAP Guidelines Development Group. PFA involves humane, supportive and practical help to fellow human beings suffering serious crisis events. Crisis responders around the world expressed a need for resource materials that could be adapted to varied socio-cultural contexts, applied to a range of emergency events and easily translated between languages. In particular, there were no widely-agreed upon resources for use in low and middle countries that suffer disproportionately from effects of humanitarian emergencies. In 2011, WHO, War Trauma Foundation and World Vision International launched the *PFA: Guide for Field Workers* to address these requests for resources. The guide underwent an extensive process of international peer review, incorporating input from over 60 professionals from a variety of socio-economic contexts. Endorsed by 24 international humanitarian agencies it reflects the emerging science and international consensus on supporting people in the immediate aftermath of extremely stressful events. Since its release, it has been translated into over 8 languages and used in extensively in varied contexts to orient professionals and volunteers. For staff and volunteers helping in emergencies, the guide offers information on the most supportive things to say and do for people in distress, approaching a crisis situation safely, and supporting people in ways that respect their dignity, culture and abilities. The guide is a model requiring adaptation to the varied contexts in which it is applied. The dilemmas and creative solutions that arise in translating and adapting the guide—as well as modifying training and orientation approaches —will be presented as lessons learned. The larger question of whether PFA reflects a universal world model of shared human responses and recovery modes following trauma will be explored.

## Open Papers: Cultural issues

### Long-term adaptation of Holocaust among survivors living at the site of their victimization 11:45–12:00

#### B. Kahana^1^, E. Kahana^2^, J. Lee^2^ and T. Bhatta^2^

^1^Cleveland State University, Cleveland, OH, USA; ^2^Case Western Reserve University, Cleveland, OH, USA

This paper explores unique challenges faced by Holocaust survivors who continue live where their victimization was perpetrated. Our study was based on interviews with 104 elderly Holocaust survivors living in Hungary. This group of survivors also endured serial psychological trauma during the communist era and after the fall of communism. We considered the cultural context of post-traumatic adaptations by comparing the reports of Holocaust survivors living in the United States or to Israel. In our interview of Hungarian Holocaust survivors, their perceptions on the conditions of their life during five critical historical periods after the end of World War II were rated. Findings reveal that Hungarian survivors perceived their life the most difficult during the height of communism in the Stalin era. They also identified their sources of concerns as economic hardships and political issues such as anti-semitism. Concerns about anti-Semitism reemerge during the brief 1956 revolution and after subsequent historical periods. Problems with social integration as well as family conflicts are found among Hungarian survivors compared to survivors in the States and in Israel. The divorce rates were seven times higher among Hungarian survivors compared to Israeli and by American survivors. Furthermore, themes from qualitative data reveal themes of family strife among the sample in Hungary, exemplified by elderly parents who are distant from their adult children. Furthermore, survivors living in Hungary also reported significantly greater psychological distress than their counterparts who relocated to new homelands. Consistent with prior research with other groups of victims experiencing trauma (Lawyer et al., 2006), our findings confirm that remaining in a previously traumatizing environment may have adverse psychological effects on the well-being and adaptation of trauma survivors.

### Adversity in childhood predicts depressive symptoms and suicide attempts at women's community 12:00–12:15

#### A. Maia, V. Pinto and J. Alves Escola de Psicologia, Universidade do Minho, Braga, Portugal

Exposure to adversity in childhood has been associated with depressive symptoms and suicide attempts in adulthood. The objectives of this study were to examine the prevalence of 10 types of self-reported adverse experiences in adult women in the community and assessed whether these experiences were predictive of depressive symptoms and suicide attempts. Two hundred and twenty-five Portuguese women, aged between 18 and 78 years, completed *Adverse Childhood Experiences (ACE) Study Questionnaire* and the depression subscale of the *Psychopathological Symptom Inventory* (BSI). Almost 96% of women reported having experienced at least one adverse experience during childhood and adolescence. In a linear regression, adversity explains 6.6% of the variance in depressive symptoms. Logistic regression indicated that for every one point increase in the standard deviation of adversity total, the risk of suicide attempts is increased by 1.818 times. Exposure to adverse experiences during childhood is frequent and the degree of exposure is a predictor of depressive symptoms and suicide attempts. It is essential to develop prevention and intervention programs in the community in order to promote improved health in this context.

### Arrivals: migrants’ stories through the use of images 12:15–12:30

#### D. Manduri Istituto Terapia Familiare di Bologna, Italy

Starting from the difficulty of listening and supporting migrants and, in particular, their stories and their pain, the Institute of family therapy of Bologna has created and tested a technique of storytelling that departs from what images suggest to migrants. In doing this, the institute has integrated two techniques of training and family therapy: the systemic-relational thinking and the use of images. We have created an ad-hoc narrative-tool, making use of Shuan Tan's graphic novel ‘The Arrival’. The highly suggestive images are viewed, chose and narrated in groups, by migrants who have known each other for a period of time. We tested ‘Arrivals’ in five classes of migrants studying Italian, for a total of 100 migrants. This paper will explore some of the results of our experimentation and provide some preliminary conclusions, which seem to tell us that images facilitate the recall, recount, acceptance and understanding of complex migrant stories, including those rich and painful.

### An epidemiological study of substance use among Karbala university students in Iraq in 2010 12:30–12:45

#### A. Al-Mousawi^1,2^ and A. Lovell^3^

^1^Karbala Medical college; ^2^Chester University Chester; ^3^Department of Mental Health and Learning Disability, Health and Social Care Faculty, Chester University, UK

*Objective*: To determine substance (tobacco, alcohol and illicit drug) use prevalence rates and trauma and Post-Traumatic Stress Syndrome and symptoms among Karbala University students in Iraq in 2010 and their associations. *Background*: Substance (tobacco, alcohol and illicit drug) use disorders are a major and a rapidly growing worldwide problem. Smokers’ number in the world exceeds 1.3 billion while alcohol disorders affect 76 million. *Methods*: A total of 5446 students in Karbala University participated voluntarily in a cross sectional survey in 2010. Based on widely used reliable and valid self-report questionnaires, the survey instrument was prepared and piloted before implementation. Chi-square and multinomial logistic analyses were used at a significance level of <0.001. *Results*: The prevalence rate for current cigarette smoking was 10% while for shisha was 8%. Although most smokers (83%) reported a desire to stop smoking, only 4% quitted smoking. The majority (84%) began smoking by the age of 20. Smoking and alcohol consumption was significantly higher among: males, older age, married, evening study students, environmental smoke exposed students and those with more smoker friends. Parental smoking and fathers’ higher education significantly increased their children smoking. Alcohol drinking prevalence was very low (2%) and was significantly higher among smokers. Lifetime illicit substance use prevalence rates were: 19% for sedative pill, 13% for codeine containing cough while it was low (1–3%) for other illicit drugs. Males had a significantly higher trauma exposure than females and mean trauma frequency. All smoking output variables and illicit drug use were significantly higher with exposure and frequency of trauma. Post-Traumatic Stress Disorder and symptoms showed positive association with smoking and illicit drug use behaviours. *Conclusions*: Significant association were found between substance use and gender, marital state, parent and friends smoking. Predictors with significantly high odds ratios for substance use behaviors were determined.

### The advantages of art therapy in PTSD treatment 12:45–13:00

#### K. A. Schouten Foundation Centre ‘45, Diemen, The Netherlands

Art Therapy treatment is an integrated part of Foundation Centre '45, partner in Arq. In clinical practice Art Therapy shows good results and several experts describe the benefits. Art Therapy offers a safe way to access and express feelings and memories about traumatic experiences. And especially with traumatized refugees from several cultures, it provides an intercultural language: the language of art. Art Therapy might provide a safe and graduate treatment. The visual and experiential characteristics of Art Therapy correspond with the nature of traumatic memories. The inability to put traumatic experiences into words appears to underline the indication of art therapy in PTSD treatment. Preliminary investigations showed that 1) there is some proof that Art Therapy interventions might be effective in reducing PTSD symptoms and depression, and 2) Art Therapy experts seems to have consensus about the efficacy of art therapy in PTSD treatment. In Foa's guidelines three recommendations for the Arts Therapies are described: randomized controlled research; developing and testing specific Art Therapy treatment for PTSD and further research of intercultural art therapy as effective intervention to bridge barriers of language and culture. In this presentation the preliminary results of the feasibility study of Art Therapy according to protocol as PTSD treatment for adults will be presented and illustrated with examples from clinical practice.

## Afternoon
*Open Papers: Military research*

### Effects of home on the mental health of British forces serving in Iraq and Afghanistan 15:15–15:30

#### N. Greenberg^1^, N. Jones^2^, K. Mulligan^2^ and M. Davies^2^

^1^Academic Centre for Defence Mental Health, Weston Education Centre, London, UK; ^2^Academic Centre for Defence Mental Health (ACDMH), King's College London, London, UK

*Background*: Most studies of the mental health of UK armed forces focus on retrospective accounts of deployment and few sample personnel while they are deployed.*Aims*: This study reports the results of a survey of deployed personnel, examining the perceived impact of events at home and military support for the family on current mental health during the deployment. *Method*: Surveys were conducted with 2042 British forces personnel serving in Iraq and Afghanistan. Prevalence of common mental disorders was assessed with the 12-item General Health Questionnaire (GHQ-12) and posttraumatic stress disorder (PTSD) was assessed with the PTSD Checklist – Civilian version (PCL-C). *Results*: The prevalence of common mental disorders was 17.8% and of probable PTSD was 2.8%. Perceived home difficulties significantly influenced the mental health of deployed personnel; the greater the perception of negative events in the home environment, the greater the reporting of adverse mental health effects. This finding was independent of combat exposure and was only partially mitigated by being well led and reporting subjectively good unit cohesion; however, the effect of the totality of home-front events was not improved by the latter. Poor perceived military support for the family had a detrimental impact on deployment mental health. *Conclusions*: The armed forces offer many support services to the partners and families of deployed personnel and ensuring that the efforts being made on their behalf are well communicated.

### The role of victims associations on the adaptation process: roles and meanings from the point of view of the war handicapped veterans 15:30–15:45

#### A. Maia^1^, J. Mendes^2^, L. Sales^2^, P. Araujo^2^, A. Dias^2^ and R. Lopes^2^

^1^ CIPsi University of Minho and Centre for Social Studies of Coimbra University; ^2^Trauma Centre/Centre for Social Studies of Coimbra University, Coimbra, Portugal

*Introduction*: The role of victim's associations in the adaptation process after trauma exposure is still poorly understood. The aim of this qualitative study was the comprehension of the experience of handicapped veteran's in a victims association. We intended to explore the meaning attributed to this participation and how it contributes to the process of recovery and rehabilitation. *Methods*: We conducted interviews with *Handicapped of the Armed Forces Association* (ADFA) members. We looked at the coping strategies and the meaning attributed to each, with an emphasis on how participation in the association is conceptualized by the handicapped veterans. *Results*: Victims report individual strategies as more relevant than social oriented strategies, and lack of social resources. Some use self-distraction coping strategies like refuge on work or art as a way of emotional expression. There are also descriptions of non-efficient coping strategies, like alcohol abuse or isolation. In the other hand participation on the association activities has a meaning of struggle for rights. Most of the individuals report the importance of their participation in group activities with people who went through the same kind of experience. This participation seems to contribute to a shared identity as victim and as handicapped. Informal meetings, as are annual lunches of the associates, appear to be crucial for their sense of identity. *Conclusion*: The literature is not clear on the relevance of belonging to victims associations to the coping process after trauma. This study suggests that belonging to ADFA bring to subjects a sense of identity and the feeling of legal rights struggle. Those two meanings may represent important contributions to the adaptation process, but have the risk of offering a “rigid” identity as victim and handicapped, instead of promoting alternative views of themselves.

### The diagnostic accuracy of the posttraumatic stress disorder checklist (PCL-C) in the context of military psychological screening 15:45–16:00

#### A. Searle^1^, M. Van Hooff^1^, A. Mcfarlane^1^, C. Davies^2^, K. Fairweather-Schmidt^1^, S. Hodson^3^, H. Benassi^4^ and N. Steele^4^

^1^Centre for Traumatic Stress Studies, University of Adelaide, Adelaide, Australia; ^2^Data Management and Analysis Centre, Discipline of Public Health, University of Adelaide, Adelaide, Australia; ^3^Department of Veterans Affairs, Canberra, Australia; ^4^Mental Health, Psychology and Rehabilitation Branch, Joint Health Command, Canberra, Australia

*Introduction*: Post-traumatic stress disorder (PTSD) is prevalent among Allied Forces military personnel, especially in the current climate of repeated Middle East deployments. Several military forces, including the Australian Defence Force (ADF), routinely screen for PTSD with the widely-used and well-validated Post-traumatic Stress Disorder Checklist (PCL-C). However, it is unknown whether the established cut-off of 50 is optimal for military personnel. This has important implications given that personnel's scores may ultimately result in receipt of treatment, or conversely, social stigma or career harm (e.g., inability to deploy). This study is the first to test the diagnostic accuracy of the PCL-C in a large representative military population (the ADF). *Methods*: In the ADF Mental Health Prevalence and Wellbeing Study, a large representative sample of currently-serving Navy, Army and Air Force members (*n*=24,481) completed the PCL-C. Then, a stratified subsample (*n*=1798) completed a structured diagnostic interview to detect 30-day PTSD. Using demographic information from military records, data were weighted to represent the entire ADF population (*n*=50,049). *Results*: Results of ROC analyses showed the PCL-C had good overall diagnostic efficiency, with an area under the ROC curve of .85. The optimal screening cut-off of 29 showed a good balance of high sensitivity and specificity, and was very similar to the cut-off (1) currently used by the ADF, and (2) identified as optimal in a sample of US military personnel. Using its established cut-off (of 50), the PCL showed poor sensitivity (though high specificity), and did not identify the majority of personnel with PTSD. *Conclusions*: The PCL represents a cost-effective and clinically useful means of screening for PTSD in the ADF. Results also support the assertion that military populations may need a less-stringent screening cut-off than civilian populations. The established cut-off appears more useful for epidemiological research purposes.

### Resources and strategies used by handicapped veterans 16:00–16:15

#### A. Maia^1^, J. Mendes^2^, L. Sales^2^, P. Araujo^2^, A. Dias^2^ and R. Lopes^2^

^1^CIPSI University of Minho and Centre for Social Studies, University of Coimbra; ^2^Trauma Centre/Centre for Social Studies, University of Coimbra, Portugal

*Introduction and aim*: Most research resorts to coping questionnaires to assess the strategies used after traumatic exposure, analyzing the relation between coping and adaptation. The aimed of this study is to describe coping strategies, including the type of social, public and informal resources to which war veterans make us of, and their perception of the utility of these resources. *Method*: Members of the Portuguese *Handicapped of the Armed Forces Association* (ADFA) were invited to participate and report on all the resources they used and their level of satisfaction with them. Participants answered a Questionnaire built where a list of NGO's, public institutions and private services were included. Other strategies, as religious practices and social support, were also included. Finally we measured coping strategies (Brief COPE), PTSD symptoms (PCL), global psychopathology (BSI) and global satisfaction in various areas of life. *Results*: Preliminary analysis shows a very low use of most social resources. Most of the participants report no use of public institutions, other than medical services. Change in religious beliefs was reported by 22% of the participants and actual symptoms are correlated to most of coping strategies in unexpected ways: for example, social and instrumental support coping and expression of emotions is related to higher PTSD symptomatology. *Discussion*: These preliminary results show that the amount of resources used, when compared with the available institutions, is very low. Future analysis and the inclusion in the study of qualitative data would help us understand specific characteristics of this group in order to suggest intervention strategies that suit their characteristics and needs.

### Prevalence and correlates of self-reported psychiatric illness and suicidality in treatment-seeking Canadian peacekeeping and combat veterans with PTSD 16:15–16:30

#### J. D. Richardson^1^, K. St Cyr^2^, A. McIntyre-Smith^2^ and J. Elhai^3^

^1^Department of Psychiatry and Behavioral Neuroscience – McMaster University/Operational Stress Injury Clinic- St. Joseph's Health Care London-Parkwood Hospital, London, UK; ^2^Operational Stress Injury Clinic- St. Joseph's Health Care London-Parkwood Hospital, London; ^3^Department of Psychology, University of Toledo, Toledo, USA

The impact of peacekeeping and combat on PTSD, depression, and anxiety in military personnel is well-documented. Clinician-administered mental health assessments require substantial resources to administer; therefore the use of client-administered questionnaires can be useful in the screening process. This study examined the prevalence of self-reported PTSD and comorbid depressive and anxiety disorders; and the associated suicide ideation. Actively serving Canadian Forces members and veterans seeking treatment at the Parkwood Hospital Operational Stress Injury Clinic (*N*=279) completed measures such as the PRIME-MD Patient Health Questionnaire (PHQ), *modified* Brief Traumatic Brain Injury Survey (BTBIS), the PTSD checklist (PCL-M). The majority ofpatients met criteria for a depressive disorder (79.6%, *n*=232), while 73.1% (*n*=201) screened positively for PTSD. In regression analyses, the number of items endorsed on the PCL-M was significantly associated with suicide ideation (β=0.425, *p*=0.000), however after controlling for depression severity and possible mTBI, number of PCL-M items was not significantly associated with suicide ideation (β=0.013, *p*=0.866). Depression severity emerged as the most significant predictor of suicide ideation (β=0.661, *p*=0.000).

## Open Papers: Effects of abuse and violence

### Understanding torture with Rorschach Inkblot Test: torture and its psychological effects on personality structure 16:45–17:00

#### C. Alozkan and M. Paker Psychology Department, Istanbul Bilgi University, Istanbul, Turkey

This study mainly investigates the torture's effects on the personality structures. As a part of an extensive research project of torture and its psychological effects which held in Turkey between the years of 1990–1995, Rorschach Inkblot Test has been applied to the four groups of participants (*tortured/activist*=*26*, *tortured/non-activist=25, non-tortured/activist*=30, *non-tortured/non-activist*=*28*) and the protocols were coded using Exner's Comprehensive System (1990). ANOVAs were carried out to analyze torture's effects on the six main axis of Rorschach and the results yield significant effects of being tortured on the cognitive and affective characteristics, self perception and the interpersonal world. Main effect of political activism was also investigated and significant effects on the included contents, ideation and self perception were found. Interactions of the two main effects were also significant and showed how political activism status may change the effects of being tortured. This study is important since it is one of the first controlled studies that investigated the relationship with torture and Rorschach by considering the subjective meaning of political activism.

### Perspectives on overlapping psychopathological symptoms and PTSD diagnosis in a sample of adult survivors of institutional abuse: a comparison of DSM-IV versus DSM-5 criteria 17:00–17:15

#### M. Knefel, V. Kantor, D. Weindl, A. Butollo, R. Jagsch, Y. Moyand and B. Lueger-Schuster Department of Clinical Psychology, Faculty of Psychology, University of Vienna, Vienna, Austria

*Background*: Institutional abuse (IA) has very severe consequences in terms of psychopathological symptoms for adult survivors. Not only PTSD but many other psychological disorders have been observed in studies of those adults who lived in an institution and experienced various forms of abuse (sexual, physical, emotional). Questions of interest in this context are: Is PTSD an adequate measure to describe the existing underlying psychopathology? Which psychopathological symptoms are else present and in how far do they overlap? Are different people diagnosed with PTSD using the new DSM-5 criteria compared to DSM-IV criteria? *Method*: 184 adult survivors of IA committed by members of the Austrian Catholic Church filled in self-report questionnaires: The PTSD Checklist—Civilian Version (PCL-C), the Brief Symptom Inventory (BSI), and the Coping Inventory for Stressful Situations (CISS), among others. New DSM-5 criteria were operationalized via single BSI and CISS items that covered diagnosis relevant symptoms. *Results*: In our sample, 89 persons (48.4%) fulfilled the DSM-IV criteria for PTSD. All of them also reported other psychopathological symptoms (BSI) such as anxiety and depression. 84.9% of all 184 persons showed clinical relevant symptoms on at least one out of ten symptom dimensions (nine BSI subscales and PTSD). Using DSM-5 criteria (B-E), prevalence dropped to 40.2% (74 persons). 16 persons did not fulfil PTSD criteria any longer; only one person fulfilled DSM-5 criteria without fulfilling DSM-IV criteria. All of those 16 persons scored positive on at least one BSI scale, indicating other psychological problems. *Conclusion*: These findings allow a very comprehensive view at psychopathology following IA. PTSD does not seem to be a sufficient diagnosis for psychopathology of survivors of IA. DSM-5's PTSD criteria are narrower and diagnose fewer people with PTSD than DSM-IV. Clinical implications are being discussed.

### Narrative exposure therapy and physioterapy in treatment of torture survivors 17:15–17:30

#### H. Stenmark, M. Hellen, T. Hogstad and S. Nomat Regional Centre on violence and traumatic stress, St. Olav University Hospital, Trondheim, Norway

Studies on treatment of torture survivors with PTSD have shown conflicting findings. Some studies show little effect on symptoms of PTSD after treatment, while other studies show some effects of treatment, even though a proportion of the torture survivors do not improve. The need to explore better treatment options is therefore evident. This study explores the use of a prolonged version of Narrative Exposure Therapy combined with Physiotherapy to help torture survivors diagnosed with PTSD and severe body pain. Nine torture survivors were screened before treatment with Clinician Administered PTSD Scale, Hamilton Rating scale for Depression and Numeric Pain Rating Scale, and then screened again three months after treatment and six months after treatment. The patients also completed rating scales for PTSD symptoms and pain after every session. In the presentation preliminary results will be presented along with clinical vignettes from the treatment.

### Impact of childhood life events and trauma on the occurrence of depressive and anxiety disorders 17:30–17:45

#### J. Hovens Leiden University Medical Center, Leiden, The Netherlands

*Objective*: To investigate the effect of childhood life events and childhood trauma on the occurrence of depressive and/or anxiety disorders over a 2-year period. *Methods*: Longitudinal data were collected from 1167 adult participants, without a baseline diagnosis of depressive or anxiety disorder, in the Netherlands Study of Depression and Anxiety (NESDA). Childhood life events and childhood trauma at baseline were assessed with a semi-structured interview. The CIDI based on DSM-IV criteria was used to diagnose depressive and/or anxiety disorders over a 2-year period. *Results*: At baseline, 14.7% reported at least one childhood life event and 35.3% any childhood trauma. Childhood life events did not predict the occurrence of depressive or anxiety disorders. During 2 years of follow-up, 226 (19.4%) of the participants developed any depressive or anxiety disorder. Emotional neglect, psychological, physical and sexual abuse were all associated with an increased risk of occurrence of either depressive disorders or comorbid anxiety and depressive disorders. However, when the presence or absence of lifetime psychopathology was taken into account, emotional neglect remained the only predictor (p=0.048). The occurrence of depressive and/or anxiety disorders seemed to be mediated through a higher baseline severity of depressive symptoms and the presence of a prior history of a disorder at baseline. *Conclusion*: Emotional neglect was prospectively associated with an increased risk of the occurrence of depressive disorders and comorbidity over a 2-year period, in adults without baseline psychopathology, likely mediated through subsyndromal depressive symptoms and kindling effects.

## ORAL, JUNE 8 HALL FALCO

## Morning
*Invited symposium: State of the art of cortisol, propranolol in the context of PTSD treatment*

### Footprints: A translational approach to advance treatment for anxiety disorders 10:00–10:15

#### M. Kindt
University of Amsterdam, Amsterdam, The Netherlands

Until the end of the 20st century, a dominant view in psychology and psychiatry was that once fear memory traces are formed, they are resistant to change. In the last decade, there is increasing evidence that upon retrieval a previously formed fear memory can return to a destabilized phase requiring protein synthesis to be re-stabilized. This opens a window of opportunity to weaken or even erase a previously formed fear memory. In a series of laboratory studies we showed that the systemic administration of propranolol, either before or after memory retrieval, erases previously learned fear responding in humans. Propranolol is a relatively non-toxic drug that passes the blood brain barrier and is supposed to block the beta-adrenergic receptors in the amygdala, thereby reducing the release of norepinephrine – a neurotransmitter crucial in the protein synthesis required for the synaptic changes underlying the formation and reconsolidation of fear memory. At present, cognitive behavioural therapy is the dominant and most effective intervention for people suffering from emotional disorders such as PTSD. Yet, in many cases, the results are short-lived, and the fear returns with the passage of time. One notable finding in our studies on disrupting the reconsolidation of fear memory is that after participants had been treated with propranolol combined with memory reactivation, the fear memory expression did not return when triggered by well-established retrieval techniques for fear responses. This indicates that the fear memory is either fully eradicated, or is no longer accessible. Thus, while the participants could still remember the fear association, this memory no longer generated the emotional response of fear. These findings are promising and seem to open new avenues for treatments of patients suffering from excessive fear such as trauma victims or people with other anxiety disorders. Even though the fear-reducing effect in the laboratory is strong, the effect is also subtle by showing that retrieval per se is not sufficient to destabilize the fear memory and hence to disrupt the process of fear memory reconsolidation. In this talk, I will present a series of studies demonstrating some necessary conditions for disrupting reconsolidation of fear memory by beta-adrenergic blockade. We show that the destabilization of fear memory depends on whether memory retrieval engaged an experience of new learning. In addition, our findings provide insights into the transition from memory retrieval - to reconsolidation - to extinction. Finally, I will touch upon the promises and challenges of translational research to develop better treatments for emotional disorders such as PTSD.

### Is hydrocortisone an effective pharmacological treatment of intrusive memories in PTSD? - a randomized, placebo-controlled, crossover study 10:15–10:30

#### P. Ludäscher Zentralinstitut fuer Seelische Gesundheit, Mannheim, Germany

*Background*: Posttraumatic stress disorder (PTSD) is characterized by traumatic memory processing. It is an ongoing debate whether reduced cortisol secretion in these patients might promote PTSD symptoms. Extensive evidence indicates that elevated glucocorticoid levels impair the retrieval of emotionally arousing information. Hence, the hypothesis was proposed that elevation of cortisol might decrease risk and symptoms of PTSD by inhibiting retrieval of traumatic memories. *Methods*: We conducted a study with two doses of hydrocortisone within a double-blind, randomized, placebo-controlled, cross-over design. 30 participants with PTSD were assigned to either : 1) 1 week placebo- 1 week hydrocortisone (10 mg/d)- 1 week placebo- 1 week hydrocortisone (30 mg/d) or 2) 1 week hydrocortisone (30 mg/d) - 1 week placebo- 1 week hydrocortisone (10 mg/d) - 1 week placebo. The primary outcome was the frequency and the intensity of intrusions assessed three times per day. *Results*: We could not find any differences of the frequency and the intensity of intrusions between 10 mg hydrocortisone- 30 mg hydrocortisone- and placebo condition. Overall symptomatology also did not differ between the three conditions. *Conclusions*: For the first time we included a sample size of 30 female participants with PTSD to test the impact of hydrocortisone on automatic memory retrieval. However, we could not replicate previous findings showing a significant impact of hydrocortisone on automatic memory retrieval. Regarding the small sample sizes of previous studies the results of our study challenge the idea of a positive treatment effect of hydrocortisone on symptoms of PTSD.

### Increased Plasma Levels of Endocannabinoids and Related Primary Fatty Acid Amides in Patients with Post-Traumatic Stress Disorder 10:30–10:45

#### I. Tatjana Kolassa^1,2^, D. Hauer^3^, H. Gola^1,2^, P. Campolongo^4^, J. Morath^1,2^, B. Roozendaal^5^, G. Hamuni^1,2^, A. Karabatsiakis^2^ and G. Schelling^3^

^1^Center of Excellence in Psychotraumatology, Department of Psychology, University of Konstanz, Germany; ^2^Clinical and Biological Psychology, Institute of Psychology and Education, University of Ulm Germany; ^3^Departments of Anaesthesiology, Ludwig-Maximilians-University, Munich, Germany; ^4^Department of Physiology and Pharmacology, Sapienza University of Rome, Rome, Italy; ^5^Donders Institute for Brain, Cognition and Behaviour, Radboud University Nijmegen, The Netherlands

Endocannabinoids (ECs) and related N-acyl-ethanolamides (NAEs) play an important role in the regulation of our stress response, anxiety and fear, as well as the encoding, recollection and extinction of traumatic memories. Since circulating EC levels are elevated under acute mild stressful conditions in humans, we hypothesized that individuals with traumatic stress exposure and post-traumatic stress disorder (PTSD) would also show alterations in plasma EC and NAE levels. We determined the plasma concentrations of the two ECs (anandamide, ANA; 2-arachidonoylglycerol, 2-AG), and the levels of four NAEs (palmitoylethanolamide, PEA; oleoylethanolamide, OEA; stearoylethanolamine, SEA; N-oleoyldopamine, OLDA) in individuals with PTSD, trauma-exposed individuals without PTSD, and healthy controls. Individuals with PTSD showed significantly higher concentrations of ANA, 2-AG, OEA, SEA and significantly lower OLDA levels than healthy controls. Trauma-exposed individuals took an intermediate position in all ECs and NAEs (except for 2-AG) between PTSD patients and healthy controls. PTSD patients had significantly higher 2-AG and PEA levels than trauma-exposed individuals without PTSD. PTSD symptom severity (as measured by the Clinician Administered PTSD Scale) correlated positively in trauma-exposed individuals (traumatized persons with and without PTSD) with PEA and negatively with OLDA. CAPS subscores for intrusion, avoidance and hyperarousal were negatively associated with OLDA levels. Individuals with PTSD but also trauma-exposed individuals without PTSD show changes in plasma ECs/NAEs concentrations. The specific functional role of EC and NAE alterations in PTSD pathophysiology needs to be determined in future studies.

### Effects of cortisol on memory retrieval in patients with PTSD 10:45–11:00

#### K. Wingenfeld Department of Psychiatry, Charité Universitätsmedizin Berlin, Campus Benjamin Franklin, Germany

In posttraumatic stress disorder (PTSD) enhanced negative feedback of the hypothalamus pituitary adrenal (HPA) axis is a prominent finding which has often been interpreted in the context of enhanced glucocorticoid receptor (GR) sensitivity (Yehuda 2009; Rohleder, Wolf et al. 2010). Neuropsychological alterations are also an important feature in PTSD. Problems particularly with learning and memory have been found, including deficits in verbal declarative memory as well as autobiographical memory. In healthy humans, most studies suggest impairing effects of glucocorticoids on memory retrieval (Wolf 2009). Up to now, studies that investigate the effects of cortisol administration on memory in patients with PTSD are rare and yielded inconclusive results (Bremner, Vythilingam et al. 2004; Grossman, Yehuda et al. 2006; Yehuda, Harvey et al. 2007; Yehuda, Golier et al. 2010). In a placebo controlled cross over study we compared the effect of exogenous cortisol on memory retrieval in PTSD patients (N=44) with the effects in healthy controls (N=65). Opposing effects of cortisol on memory were observed when comparing patients with controls. In controls, cortisol had impairing effects on memory retrieval, while in PTSD patients cortisol had enhancing effects on memory retrieval. The present results suggest beneficial effects of acute cortisol elevations on hippocampal mediated memory processes in PTSD. Possible neurobiological mechanisms underlying these findings are discussed.

Reference

Bremner, J. D., Vythilingam, M., et al. (2004). Effects of dexamethasone on declarative memory function in posttraumatic stress disorder. *Psychiatry Res* 129(1): 1–10.

Grossman, R., Yehuda, R., et al. (2006). Cognitive effects of intravenous hydrocortisone in subjects with PTSD and healthy control subjects. Ann N Y Acad Sci 1071: 410–421.

Rohleder, N., Wolf, J. M., et al. (2010). Glucocorticoid sensitivity of cognitive and inflammatory processes in depression and posttraumatic stress disorder. *Neurosci Biobehav Rev* 35(1): 104–114.

Wolf, O. T. (2009). Stress and memory in humans: twelve years of progress? *Brain Res* 1293: 142–154.

Yehuda, R. (2009). Status of glucocorticoid alterations in post-traumatic stress disorder. *Ann N Y Acad Sci* 1179: 56–69.

Yehuda, R., Golier, J. A., et al. (2010). Hydrocortisone responsiveness in Gulf War veterans with PTSD: effects on ACTH, declarative memory hippocampal [(18)F]FDG uptake on PET. *Psychiatry Res* 184(2): 117–127.

Yehuda, R., Harvey, P. D., et al. (2007). Enhanced effects of cortisol administration on episodic and working memory in aging veterans with PTSD. *Neuropsychopharmacology* 32(12): 2581–2591.

## Workshop: Children following disaster: PTSD predictors and risk factors

### Children Following Disaster: PTSD Predictors and Risk Factors 11:45–12:45

#### E. de Soir^1^, L. Scaut^2^

^1^Royal Higher Defence College, Brussels, Belgium; ^2^De Weg-Wijzer Centrum voor Psychotherapie, Leopoldsburg, Belgium

This workshop aims to discuss the risk factors for the development of posttraumatic stress reactions (PTSR) in children and youth involved in disaster. Results from previous scientific research indicate that several risk factors are related to the development of PTSD; the type of exposure to the disaster and peritraumatic dissociation during or immediately after the disaster, i.e. peritraumatic dissociation. In a first part, the presenters will provide a theoretical overview from literature on children involved in large scale accidents and disasters. During this part of the workshop, a recent research on child survivors of the Ghislenghien gas explosion (Belgium, July 30th 2004) will be presented. In a second part, recent disaster situations in Belgium i.e. the Pukkelpop Rock disaster (August 2011) and the Sierre bus disaster (March 2012) in which 22 children died - will be used to present a community driven and practice-based trauma intervention model.

## Afternoon The spectrum of trauma-related disorders
*
Symposium: Trauma, PTSD and substance use*

### Alcohol abuse in war-affected rural communities in Northern Uganda? 15:15–15:30

#### R. Saile, F. Neuner, V. Ertl and C. Catani Bielefeld University, Bielefeld, Germany

During two decades of civil war in Northern Uganda, almost the entire population was forcibly displaced into Internally Displaced Person (IDP) camps. Besides a multitude of adversities characterizing life in the camps, hazardous alcohol use, especially amongst men, emerged as a major problem as it has been linked to a loss of functioning and an escalation of partner violence in intimate relationships. Although war exposure appears to be associated with higher levels of hazardous alcohol use, little is known about the relationship between trauma exposure, psychopathology, and hazardous drinking in post-conflict societies. The current study presents preliminary findings on hazardous alcohol use in seven heavily war-affected communities in Northern Uganda after most IDPs have left the camps. We tested previous trauma exposure and posttraumatic symptoms as potential risk factors for higher levels of alcohol-related problems and subsequently examined hazardous alcohol use in men as a predictor of female experiences of partner abuse. Analyses are based on data from structured interviews with 365 women and 304 men comprising 235 couples. We employed the Alcohol Use Identification Test (AUDIT) to collect information on alcohol-related problems. We found that 46% of men and 1% of women engaged in hazardous drinking. In men, childhood maltreatment, but not war-related trauma exposure, predicted higher levels of hazardous drinking. There was a trend towards more alcohol-related problems when men suffered from more severe PTSD symptoms, whereas depressive symptoms were associated with less hazardous alcohol use. When controlling for a range of risk factors, alcohol-related problems in male partners proofed a significant predictor of female-reported experiences of partner abuse. Alcohol abuse amongst men in Northern Uganda is still widespread and future research is needed to assess the extent of alcohol-related disorders and risk factors for hazardous alcohol use in order to develop effective prevention and treatment programmes.

### Childhood trauma and its association with prolonged use of benzodiazepines in opioid-maintained patients 15:30–15:45

#### M. Vogel^1^, K. Duersteler-MacFarland^1^, M. Walter^1^, J. Strasser^1^, S. Fehr^1^, L. Prieto^2^ and G. Wiesbeck^1^

^1^Division of Substance Use Disorders, Psychiatric University Clinics, Basel; ^2^London School of Hygiene & Tropical Medicine

The association of traumatic experiences with the development of substance use has been demonstrated in a wide variety of studies. In opioid-maintained patients, traumatic experiences are highly prevalent and are often related to adversities in childhood but also consequences of the lifestyle of individuals who are dependent on illegal drugs. A large proportion of opioid-maintained patients also use additional substances such as cocaine, amphetamines or prescription drugs. Particularly, use of benzodiazepines is common. We conducted a cross-sectional survey of 193 patients maintained on oral opioids or injectable diacetylmorphine. We assessed adverse childhood experiences with the Childhood Trauma Questionnaire (CTQ), patterns and motives for benzodiazepine use with a specifically designed questionnaire, as well as clinical diagnoses and current substance use. Sixty-seven per cent of participants reported a moderate-to-severe score in at least one subcategory of traumatic childhood experiences. Furthermore, prolonged use of benzodiazepines was associated with higher overall CTQ scores, and more moderate-to-severe subscores for emotional abuse, and emotional and physical neglect. This association remained significant while controlling for potential confounders in multivariate analysis. Corresponding to the self-medication theory, subjective motives for use were often self-therapeutic, comprising relief from anxiety, sleeping problems or traumatic memories. Trauma may be at the bottom of these symptoms, leading to the development of psychiatric comorbidities and concomitant substance use. However, conventional treatment strategies for substance using patients usually fail to address traumatic experiences. Moreover, trauma-specific therapy is often reserved for patients that have successfully addressed their substance use beforehand. In the face of failure of conventional treatment attempts, this dilemma often leads to addiction therapists prescribing benzodiazepines on a long-term basis for treatment of trauma-related symptoms or disorders such as PTSD, depression or anxiety disorders.

### PTSD and khat misuse in Somali refugees in Nairobi 15:45–16:00

#### M. Widmann^1^, J. Mikulica^1^, J. Von Beust^1^, D. Ndetei^2,3^, M. Al'absi^4^ and M. Odenwald^1^

^1^University of Konstanz, Konstanz, Germany; ^2^University of Nairobi, Kenya; ^3^Africa Mental Health Foundation, Kenya; ^4^University of Minnesota, Minneapolis, MN, USA

The leaves of the khat tree (Catha edulis) are traditionally chewed in African and Arab countries and contain the amphetamine-like alkaloid cathinone. Over the past years, Somalis have been repeatedly exposed to war, violence, famine and displacement. Here we report associations of PTSD and excessive khat use among Somali refugees living in Nairobi. We compared male Somali khat chewers (33) fulfilling the DSM-IV criteria for khat dependence and comparable non-chewers (15) of the same age. PTSD and khat dependence were assessed with the Mini International Neuropsychiatric Interview. We quantified the number of experienced traumatic events by the event list of the Somali version of the Posttraumatic Diagnostic Scale. Additionally we assessed current khat use patterns and khat use history. The studied group was heavily burdened by traumatic events and posttraumatic symptoms. Khat use patterns varied from moderate to excessive (up to 18 hours per day on average last week). Khat addicts reported more traumatic event types and had more often PTSD than non-users (27% vs. 0%, p=.04). Eighty-five percent reported functional khat use, i.e. that khat helps to forget painful experiences. Binge khat users (11) chewed khat for more than 24 hours in a row last week and reported the highest levels of trauma load and psychopathology. We found evidence for self-medication of trauma consequences by using khat among Somali refugees. Findings need to be replicated with a representative sample. Somali refugees are highly burdened by psychopathology. Adequate community-based treatments need to be developed.

### Trauma load moderates the association between motivation to change and treatment outcome in German alcohol detoxification 16:00–16:15

#### M. Odenwald^1^ and P. Semrau^2^

^1^Department of Psychology, University of Konstanz, Konstanz, Germany; ^2^Forel Clinic, Islikonerstrasse, Ellikon an der Thur, Switzerland

Motivation to change is thought to be crucial for achieving long-term alcohol abstinence, however empirical results are contradictory. Patients taking part in addiction treatment report frequently traumatic experiences and PTSD but this has not systematically been studied in terms of effects on treatment outcomes. This study aimed to clarify whether individual Trauma Load explains some of the inconsistencies between motivation to change and behavioral change. Fifty-five patients admitted to an alcohol detoxification unit were enrolled in this study. At treatment entry, we assessed lifetime traumatic experiences (Trauma History Questionnaire, THQ) and motivation to change (University of Rhode Island Change Assessment, URICA). Mode of discharge was taken from patient files after therapy. We tested with multivariate methods whether Trauma Load moderates the effect of motivation to change on dropout from alcohol detoxification. Dropout of detoxification treatment occurred in 55.4%, 44.6% were completers. Age, gender and days in treatment did not differ between completers and dropouts. Patients who dropped out reported on average more traumatic event types than completers. Treatment completers had higher scores in the URICA subscale Maintenance. Multivariate methods confirmed the moderator effect of Trauma Load: Among participants with high Trauma Load treatment completion was related to higher Maintenance and Contemplation scores at treatment entry but not among patients with low Trauma Load. We report first evidence that the effect of motivation to change on detoxification treatment completion is moderated by Trauma Load: Among patients with low Trauma Load, motivation to change is not relevant for treatment completion; among highly burdened patients, however, who have a priori a greater risk to drop out, a high motivation to change might make the difference. This finding justifies research into specific interventions for alcohol patients with severe life-time trauma history to increase their motivation to change.

## Psychobiology and PTSD
*Invited symposium: Neurobiological studies in traumatized children and adolescents*

### HPA-axis reactivity in traumatized youth; cortisol levels before and after trauma-focused psychotherapy 16:45–17:00

#### J. B. Zantvoord, K. Schmitt, J. Diehle and R. Lindauer AMC/deBascule Amsterdam, The Netherlands

Posttraumatic stress disorder (PTSD) is characterized by alternations in the HPA-axis and glucocorticoid pathway. Findings in several adult populations with PTSD indicate enhanced HPA-axis reactivity, including increased negative feedback. However the small number of endocrinological studies conducted in traumatized youth show somewhat diverged findings as compared with adults. These diverged findings render further investigation, particularly considering the substantial developmental changes in brain regions involved in the regulation of the HPA axis which take place throughout childhood and adolescence. Furthermore publications on longitudinal studies, investigating the effects of trauma focused psychotherapies on HPA-axis function, are scares in traumatized adults and nonexistent in youth. Against this background we will present our first cross-sectional and longitudinal cortisol data in traumatized youth. HPA axis reactivity was assessed by measuring salivary cortisol levels before, during and after a script driven imagery procedure. Salivary cortisol was collected in youth with PTSD (aged 8–17) and their traumatized but healthy peers. Children and adolescents diagnosed with PTSD were assessed before and after 8 sessions of trauma focused psychotherapy (either TF-CBT or EMDR). Endocrinological outcome data are correlated with PTSD symptoms scores as measures with the CAPS-CA. Results will be presented in developmental framework and potential clinical significance will be discussed.

### Gene×environment; the impact of 5HTTLPR genotype on the risk of developing posttraumatic stress symptoms in children and adolescents 17:00–17:15

#### J. Ensink, J. B. Zantvoord, E. Van Meijel, M. Gigengack and R. Lindauer AMC/deBascule Amsterdam, The Netherlands

In the past decade a vast body of epidemiological and experimental data has shown that gene by environment (G x E) interactions play an important role in the etiology of several psychiatric disorders. Because posttraumatic stress disorder (PTSD) is a disorder that is dependent on exposure to an environmental pathogen, G×E studies examining genetic risk for PTSD have been a topic of increasing interest. Twin research has shown that genes play a considerable role in the etiology of PTSD. Association studies in adults have focused on genes involved in the dopaminergic and serotoninergic systems. These studies suggests that variations in a polymorphism (5HTTLPR) of the serotonin transporter gene (5-HTT) are involved the development of PTSD in adults. However, the number of GxE studies in traumatized children exploring the role of 5-HTTLPR polymorphisms is still limited. Against this background we examined the association between the serotonin transporter gene polymorphism (G) and trauma exposure (E) in children and adolescents in a pilot study. Participants age 11 to 21, were interviewed with the Anxiety Disorder *Interview* Schedule IV (*ADIS*-IV) three months post trauma to measure PTS symptoms. To explore the impact of 5TTLPR on specific endophenotypes we measured the executive functions with the Behavior Rating Inventory of Executive Function (BRIEF). DNA was extracted from saliva samples. Saliva samples were used to differentiate between versions of the 5-HTTLPR genotype. We will present our main outcomes on PTSD symptoms and executive functions. Furthermore, we will propose future lines of GxE research in traumatized children and adolescents and discuss the potential clinical implications of findings from these studies.

### Neuroimaging in children, adolescents and young adults with psychological trauma 17:15–17:30

#### M. A. W. Rinne-Albers^1^, N. J. A. Van Der Wee^2^, F. Lamers-Winkelman^3^ andR. R. J. M. Vermeiren^4^

^1^Curium-LUMC, The Netherlands; ^2^Leiden Institute for Brain and Cognition, LUMC, Leiden, The Netherlands; ^3^Vrije Universiteit Amsterdam, The Netherlands, Kinder en Jeugd Traumacentrum Haarlem; ^4^Curium-LUMC Leiden, The Netherlands, Leiden Institute for Brain and Cognition Leiden, The Netherlands

Childhood psychological trauma is a strong predictor of psychopathology. Preclinical research points to the influence of this type of trauma on brain development. However, the effects of psychological trauma on the developing human brain are less known and a challenging question is whether the effects can be reversed or even prevented. The presentation gives an overview of neuroimaging studies in traumatized juveniles and young adults up till 2012. Neuroimaging studies in children and adolescents with traumatic experiences were found to be scarce. Most studies were performed by a small number of research groups in the United States and examined structural abnormalities. We could not identify any studies investigating treatment effects. Neuroimaging studies in traumatized children and adolescents clearly lag behind studies in traumatized adults as well as studies on ADHD and autism.

### Cross-sectional and longitudinal study of salivary cortisol and dehydroepiandrosterone sulfate in adolescent rape victims with PTSD 17:30–17:45

#### I. A. E. Bicanic^1^, R. M. Postma^2^, G. Sinnema^2^, C. De Roos^3^, M. Olff^4^, F. Van Wesel^5^ and E. M. Van De Putte^6^

^1^University Medical Centre, National Psychotrauma Centre for Children and Youth Utrecht, The Netherlands; ^2^University Medical Centre, National Psychotrauma Centre for Children and Youth, The Netherlands; ^3^Psychotrauma Centre for Children and Youth, GGZ Rivierduinen Leiden, The Netherlands; ^4^Centre for Psychological Trauma, Department of Psychiatry and Academic Medical Centre, University of Amsterdam, Amsterdam, The Netherlands; ^5^Department of Methodology and Statistics, University of Utrecht, Utrecht, The Netherlands; ^6^Department of Paediatrics, University Medical Centre, Utrecht, The Netherlands

*Background*: Rape is associated with Post Traumatic Stress Disorder (PTSD), which can be treated successfully with trauma-focused treatment. PTSD as a result of sexual trauma has been associated with dysregulation of the Hypothalamic Pituitary Adrenal (HPA) axis. In adolescent rape victims with PTSD, lower cortisol and lower Dehydroepiandrosterone Sulfate (DHEAS) have been found, but as yet no studies have examined changes in HPA-axis functioning after trauma-focused treatment. *Aims*: To examine changes in psychological and HPA-axis functioning in adolescent rape victims after trauma-focused treatment with parallel parent guidance. *Methods*: Twenty-one female adolescents with rape-related PTSD completed Cognitive Behavioural Therapy or Eye Movement and Desensitization Reprocessing. Their parents received parallel parent guidance. Basal salivary cortisol and DHEAS were assessed at pre- and post-treatment at 0, 15, 30, 45 and 60 minutes after awakening. Self-report questionnaires and a clinical interview were used to assess psychological functioning and presence of PTSD at pre- and post-treatment. Outcome data were compared with previously published data on psychological and endocrinological functioning of 37 non-traumatized controls (Bicanic et al., 2012). *Results*: Post-treatment, PTSD and depression symptoms were significantly lower than at pre-treatment. PTSD diagnosis was no longer present in 86% of the patients. Significantly higher levels of DHEAS were found post-treatment. Non-significant increases in cortisol levels were observed. Post-treatment cortisol and DHEAS levels corresponded to levels of non-traumatized controls. *Conclusion*: The findings suggest a normalization of the HPA-axis in adolescent rape victims after trauma-focused treatment with parallel parent guidance. Future randomized controlled trials should be conducted to confirm whether trauma-focused treatment is effective in changing HPA-axis functioning.

## ORAL, JUNE 8 HALL GARGANELLI

## Morning
*Open Papers: Children and young people I*

### Realities of night and day—prison rioting as a response to structural violence and severe traumatization in a juvenile facility in Georgia 10:00–10:15

#### L. Tsiskarishvili, K. Pilauri and N. Kvavilashvili The Georgian Centre for Psychosocial and Medical Rehabilitation of Torture Victims—GCRT, Ilia State University, Tbilisi, Georgia

In 2009 the Georgian government decided to shift the criminal justice system from a zero tolerance approach to a more humane and prisoner-centered approach. The EU agreed to support this undertaking financially. One of the main priorities set, was to reform the juvenile justice system. For this purpose a psychosocial unit, with trained psychologists and social workers, was set up in the juvenile correctional facility. For the first time in history Georgian juvenile delinquents were provided with access to day activity programs, vocational training programs, schooling and individual sentence planning. However, in August 2012 a massive riot took place in the juvenile facility. Most of the property and equipment was damaged and some of the convicts received mild injuries as well. In order to analyze the causes of the riot, the Georgian Centre for Psychosocial and Medical Rehabilitation of Torture Victims (GCRT) conducted a study among the juveniles who were in the facility at the time of the riot. The study consisted of a semi-structured in-depth interview and the use of a mental health needs assessment instrument—SQIFA SIFA. The study reveals that in parallel to introduced innovations (psychosocial services, schooling, etc.) the juveniles were systematically subjected to severe physical and psychological violence by the administration: group beatings, sleep deprivation, insults and humiliation of family members, among others. Interestingly enough, none of the newly hired professional staff was aware of what was going on in the facility after their working hours were over; juveniles were very strictly warned that they will would wind up in even worse conditions, if they would disclose the violence. The presentation will reflect upon the reasons why these two parallel realities in the facility existed, as well as the impact of incongruent attitude towards the juveniles on their mental health and psychosocial wellbeing.

### Perceived social support and psychological distress in terror victims: exploring the social causation and social selection hypotheses in young survivors from the shooting at the Utøya Island, Norway, July 22 2011 10:15–10:30

#### S. Thoresen^1^, T. Wentzel-Larsen^1^, T. Jensen^2^ and G. Dyb^1^

^1^Norwegian Centre for Violence and Traumatic Stress Studies; ^2^Department of Psychology, Faculty of Social Sciences, University of Oslo, Oslo, Norway

Research has repeatedly demonstrated associations between perceived social support and psychological distress. Such associations are usually interpreted as support for the social causation hypothesis, indicating a buffering effect of social support against the development of mental health symptoms. A competing explanation has received much less attention, namely the social selection hypothesis. This hypothesis proposes that healthy individuals are selected into social relationships, and individuals who develop mental health problems may risk a loss in social support. The aim of this study was to investigate the social causation and social selection hypoteses in terror victims. Survivors from the shooting at the Utøya Island in Norway July 22. 2011 participated in face-to-face interviews approximately 5 months (T1) and 14 months (T2) after the shooting. T1 includes 325 participants, with a response rate of 66%. Preliminary results for T2 is a response rate of approximately 60% of the original total population. At both time points, participants completed the Duke-UNC Functional Social Support Questionnaire (FSSQ), the UCLA Posttraumatic Stress Disorder Reaction Index (PTSD-RI), in addition to various demographic and exposure-related measures. We will investigate the social causation and the social selection hypotheses by analyzing both the relationship between social support at T1 and posttraumatic stress reactions at T2, and the relationship between posttraumatic stress reactions at T1 and social support at T2, adjusting for other relevant factors. A better understanding of the relationship between social support and mental health may have important implications for clinical practice with trauma victims.

### BEAR: building emotional and affect regulation in children 10:30–10:45

#### R. Pat-Horenczyk Mevasseret Zion, Jerusalem, Israel

A growing number of studies have investigated protective factors that can increase resilience of children in the face of adversity. There is evidence that exposure to traumatic events may impair regulation in children and the capacity for self-regulation may play a central role in coping and posttraumatic adaptation. The Building Emotional & Affect Regulation (BEAR) intervention is a new program for building resilience in children who have been removed from their homes due to exposure to significant traumatic events including physical and sexual abuse, neglect, and severe psychological illness of the parent. The program is based on our experience in building resilience in children with a new emphasis on enhancing emotional regulation as a major protective factor. We implemented a pilot of the BEAR group intervention in five children's homes in Singapore. The children living in these homes were characterized by the psychologists as suffering from outbursts of anger and aggression, excessive clinging behavior, and shame. Thirty-eight children participated in the six-session intervention which focused on enhancing self-regulation abilities in the physical, cognitive, emotional and social domains. The intervention consisted of exercises to increase mindfulness and self awareness, psycho-education, experiential and artistic activities, and enhancing social support. The pilot program was evaluated through questionnaires administered to the children, their caregivers, and the group facilitators at pre- and post-intervention and were also assessed at a 3-month follow-up. Evaluation measures focused on the child's perceived strengths and weaknesses, coping abilities, and emotional self regulation. Preliminary results show significant increase in the caregiver's perception of the child's ability to cope with day-to-day problems and their overall level of self regulation, as well as a significant reduction in the child's general level of distress.

### Evidence-based trauma interventions: how and for whom do they work? Results from “Teaching Recovery Techniques” intervention among Palestinian children 10:45–11:00

#### S. Kangaslampi^1^, K. Peltonen^1^, Q. Samir^2^, M. Diab^3^ and R. Punamaki^1^

^1^University of Tampere, Tampere, Finland; ^2^Islamic University Gaza; ^3^Gaza Community Mental Health Programme

Recent reviews of trauma interventions for war exposed children call for discovering the mechanisms underlying their effectiveness (Peltonen & Punamäki 2010; Tol et al., 2010). In other words, concerning the treatment of PTSD in children, it is important to know not just *what* is effective, but also *why* it is effective and for *whom* interventions should be targeted at. This study examines the effectiveness of a CBT-based intervention called Teaching Recovery Techniques (TRT) among 482 Palestinian school children. TRT is an intervention aimed at the secondary prevention of persistent problems and speeding up recovery after traumatic events. It is designed to be used in a group setting, most commonly and easily in schools. The group randomized control data is analyzed according to the principles of Conditional Process Modeling as described by Hayes (2012). The results suggest that the intervention was effective in reducing children's PTSD symptoms but, contrary to our expectations, not through decreasing negative cognitive appraisals or self-attributions. There was also some evidence that depressed children benefited more from the intervention than their non-depressed peers. Combining the results of this study and earlier results based on the same data (Qouta et al., 2012) we learn that certain mental health problems such as depression and peritraumatic dissosiation can affect the treatment of PTSD in different ways, even when manualized and evidence-based interventions are used. In addition, the mechanisms by which trauma interventions work must be studied in more detail.

### Media and terror victims: media contact, satisfaction and regrets in young survivors from the shooting at the Utøya Island, Norway, July 22 2011 11:00–11:15

#### S. Thoresen and G. Dyb Norwegian Centre for Violence and Traumatic Stress Studies, Oslo, Norway

When a disaster or a terror attack strikes, it is followed by massive media attention. Journalists may face ethical dilemmas in their contact with highly traumatized victims. Little is known about how victims percieve media attention and media exposure. The aim of this study was to investigate negative and positive experiences with media exposure in terror victims, and factors associated with negative or postitive perceptions. Survivors from the shooting at the Utøya Island 22 July 2011 participated in face-to-face interviews approximately 5 months (T1) and 14 months (T2) after the shooting. T1 includes 325 participants, with a response rate of 66%. Preliminary results for T2 is a response rate of approximately 60% of the original total population. In T2, the respondents were asked to report if they had or had not been contacted by media, if these contacts were perceived as positive or negative, and if they had been interviewed by media about their terror experiences and/or about their experiences with the trial. In addition, they were asked to rate the degree to which their media exposure had been perceived as stressful and/or positive, and their potential regrets about participation. We will investigate if the media experiences were associated with gender, age, level of trauma exposure, peri-traumatic stress reactions, or posttraumatic stress reactions (PTSR). Results will broaden our understanding of how media exposure is perceived in subgroups of terror victims, and may also be useful for ethical considerations in journalism.

## Open Papers: Cognition and emotion I

### “Why me?” At risk for PTSD after war and terror—sign of weakness or virtue? 11:45–12:00

#### H. K. Smebye Hospital of Østfold, Fredrikstad, Norway

Is research into pretrauma risk-factors for PTSD asking all the right questions? People surviving war and terror with PTSD may compare their fate to survivors with no lasting psychic problems and ask: “Why did I develop severe problems, while my comrades didn't?” A preliminary report after the Utøya-massacre in July 2011 found that while half of the young survivors developed symptoms close to or fulfilling a PTSD-diagnosis, 9% reported improved school results and 25% improved social functioning. If Utøya-survivors with PTSD searched published pretrauma PTSD risk-factors for an answer to their “Why me?”-question, they would find only signs of weakness: e.g. lower education, previous trauma, general childhood adversity, psychiatric history, reduced hippocampus and extinction learning. Clinical evidence from traumatized refugees with severe PTSD suggests an additional set of factors predisposing survivors for PTSD. The therapeutic approach built on Ehlers and Clark's PTSD-theory stressing the importance of appraisals of the trauma and its aftermath for developing a PTSD. These appraisals often are storylines behind nightmares. Changes in dysfunctional appraisals reduce PTSD-symptoms. This presentation will show how appraisals of many severely traumatized refugees are related to truly heroic values. Many soldiers and freedom fighters risked their own life helping others—with little regard to own safety. It is speculated that these altruistic and empathic values have predisposed them for developing PTSD: In their present nightmares they often experience fear of killing others, or accusations from persons they did not manage to save—resulting in feelings of guilt and shame. When developing their trauma-narratives, they realize that without their strong desire to help others, they would not be so troubled by their traumatic past. This suggests that developing PTSD might for some be a sign of virtue. Helping them realizing this creates an important basis for restoring their self-respect.

### Validation of health grief prototype narrative 12:00–12:15

#### V. Sousa, M. Sa, L. Ferreira and J. C. Rocha UnIPSa; Centro de Investigação em Ciências da Saúde (CICS), Instituto Superior de Ciências da Saúde—Norte, CESPU. Rua Central de Gandra, Gandra, Portugal

Bereavement is a natural and universal phenomenon that involves a continuous process of adaptations on part of the human being to integrate experienced events. The individual organizes himself and its experiences around coherent, complex and diverse narratives. To better understand the experience of healthy grief arises the relevance to determine the discourse of the prototype narrative of healthy Grief (HG). The aim is to validate this narrative and compare with other disorders prototype narratives. Participants are identified by structured interview that include the following instruments: sociodemographic questionnaire; Inventory of Complicated Grief (ICG). For Validation the HG narrative we had 28 participants with Complicated Grief (24 women and 4 men, age M=74,15 SD=22,0) and for HG group 45 participants (ICG cutoff ≤25; 38 women and 7 men, age M=22,0 SD=5,56). The results of the validation shows that this narrative is accurate with the experience of health grief, and the comparison with other prototype narratives showed significant differences and similarities that will be discussed. The divergent and convergent validation results of the HG prototype narrative allow discussion about the value of constructed narratives for clinical practice and research.

### Trauma-related guilt and posttraumatic stress among journalists 12:15–12:30

#### T. Browne^1^, M. Evangeli^2^ and N. Greenberg^3^

^1^Traumatic Stress Service, South West London & St Georges Mental Health Trust, London; ^2^Royal Holloway University, London; ^3^Academic Centre for Defence Mental Health, Kings College London, London

There is growing recognition that journalists routinely cover traumatizing events which may leave them at risk of work-related psychopathology. It was hypothesized that unique aspects of their role (e.g. to observe and not intervene) and job-specific factors (e.g. the requirement to sensationalize an event) may make them particularly vulnerable to guilt in the aftermath of a work-related traumatic event. This study investigated the relationship between trauma-related guilt and symptoms of work-related posttraumatic stress disorder (PTSD), and the role of guilt in mediating the effect of work-related traumatic events on PTSD symptoms. Fifty journalists, recently exposed to work-related trauma, completed an online questionnaire that explored their work-related experiences of trauma, PTSD symptoms, and guilt cognitions. Higher levels of exposure to work-related trauma were significantly associated with higher levels of PTSD symptoms and guilt cognitions. Guilt cognitions were significantly positively associated with PTSD symptoms, and were found to partially mediate the relationship between exposure to work-related trauma and PTSD symptoms. The association between guilt cognitions and PTSD symptoms indicated that trauma-specific cognitions might be significant in understanding the impact of work-related trauma on the mental health of journalists. The findings provide tentative support for PTSD models that focus on posttrauma appraisals, in addition to the impact of peritraumatic fear on memory. This study provides preliminary evidence of a relationship between guilt cognitions and PTSD symptoms among journalists exposed to work-related trauma. It implies there may be specific factors that increase vulnerability to guilt among certain high risk occupational groups.

Reference

Browne, T., Evangeli, M., & Greenberg, N. (2012). Trauma-related guilt and posttraumatic stress among journalists. *Journal of Traumatic Stress, 25*, 201–210.

### The moderating effects of previous losses and emotional clarity on complicated grief and traumatic stress 12:30–12:45

#### J. C. Rocha and S. I. Castro Instituto Superior de Ciências da Saúde- Norte, Cespu—Gandra, Portugal

The connections between complicated grief and traumatic stress have previously been investigated; however, the learning effects resulting from previous losses and emotional clarity are still unclear. Understanding these effects may shed more light on the general hypotheses of emotional aging (more than chronological aging). We aimed to assess the moderating effects of emotional clarity and previous losses on bereavement outcomes: complicated grief and traumatic stress Sample has 190 participants (147 female and 43 male) with a average age of 37.49 (SD=23.75) evaluated using a sociodemographic questionnaire, the Inventory of Complicated Grief, the Impact of Event Scale–Revised, and the Lack of Emotional Clarity sub-scale of Difficulties in Emotion Regulation Scale. Results show that those participants with more than two previous losses have a higher risk for Complicated Grief, however there is no evidence of an increased risk for PTSD. The moderations reveal important interaction effects: the number of previous losses change the relationship between complicated grief and traumatic stress (R2 of interception model of,469) and that emotional clarity changes the association between previous losses and complicated grief. The results are discussed on the perspective of the confounding nature of cumulative effects on both Complicated Grief and Traumatic Stress, and the key role of emotion clarity and the importance of the number previous losses to explain relations between grief and traumatic stress.

### The association between Posttraumatic Growth and Defence Mechanisms 12:45–13:00

#### M. Boerner^1^, S. Joseph^1^ and D. Murphy^2^

^1^School of Sociology and Social Policy, Nottingham, UK; ^2^School of Education, Nottingham, UK

*Background*: Posttraumatic growth has become a major topic of research investigation. As such researchers and practicing therapists have started to think about the facilitation of posttraumatic growth. However, interest in the therapeutic facilitation of posttraumatic growth could be premature as several issues remain to be resolved. Most notably, it is not clear whether posttraumatic growth is always associated with adaptive psychological processes or whether at times posttraumatic growth may result from maladaptive processes. Defensive processes may moderate stress responses and could also influence the perception of posttraumatic growth. The aim of the present study is to examine whether certain defence styles are associated with posttraumatic growth. *Methods/Design*: A convenience sample of students [N=93] of the University of Nottingham completed a questionnaire package that included the Posttraumatic Growth Inventory (PTGI), and the Defense Styles Questionnaire (DSQ-40). The DSQ-40 captures, for example, denial by the item “I fear nothing”. *Results*: Ninety three participants representing a response rate of 46.97% [58.1% females and 41.9% males] took part. They had a mean age of 22.09 [SD=3.47]. The reliability of the measurements were satisfactory (PTGI) to acceptable (DSQ-40) with the exception of the neurotic defence style of the DSQ-40 [α=.41]. In a 1-tailed test, posttraumatic growth was associated with mature [r=.341; p<.01], neurotic [r=.385; p<.01], and immature defences [r=.298; p<.01]. *Conclusion*: Mature and neurotic defences were found to be associated with posttraumatic growth. These results suggest that the report of posttraumatic growth is caused by adaptive and maladaptive defensive processes. Therefore, self-reported posttraumatic growth may be associated with mechanisms which have the potential to impede as well to facilitate the healing process.

## Afternoon
*Open Papers: Cognition and emotion II*

### South Asian women and sexual assault: honor, shame, and trauma 15:15–15:30

#### R. Correia Barts Health NHS, London, UK

People who have been sexually assaulted are often reluctant to disclose their experiences to or seek help from health professionals and only around one in ten victims of rape report the offence to the police. Seeking support following sexual assault can be particularly difficult for some individuals due to cultural, religious and family honor reasons. For example, South Asian women are often held responsible for maintaining family honor and avoiding shame, which can make it difficult for women to leave violent relationships. Arranged and forced marriages are also common amongst this population often resulting in repeat victimization and more severe and persistent mental health problems. In addition to cultural barriers to seeking support the more vulnerable are sometimes illegal in the country with no recourse to public funds, socially isolated and unable to speak English. In cases where the violence is disclosed, the women may be disowned by their families and ostracized by the community. Guilt, shame and self-blame are often reported by those who have experienced sexual violence. This study is a retrospective case note review looking at past and current mental health needs of South Asian women attending the Haven-Whitechapel (Sexual Assault Referral Centre) following acute sexual assault for forensic medical examination and/or follow-up medical and psychosocial care. Data was collected on demographics, details of the assault and services involved in aftercare. Attention is also paid to risk assessment and risk management including referral to external agencies. The clinician reflects on cultural influences and psychological difficulties experienced by South Asian women and the role of clinical psychology in alleviating distress. Cases will be discussed and clinical dilemmas commonly affecting the work with these clients will also be addressed.

### Guilt and trauma: how to survive to have caused the death of others? 15:30–15:45

#### P. Andreatta University of Innsbruck, Innsbruck, Austria

Traumatic reactions after accidents (e.g. motor vehicle accidents) and the role of attribution for the event is more focused in research in recent years. Furthermore Kubany et al. (1995) formulated a Multidimensional Model of Trauma-Related Guilt and developed the Trauma Related Guilt Inventory. The factor attribution is still controversially discussed (Sholomskas et al. 1990; Siol et al. 2003; Janoff-Bulman, 1977; Montada, 1995). The role of self-blame, taking responsibility, blaming others and such more for adaption, coping and well-being seems to be complex. In addition it is often not distinguished between “guilt feeling” and “being guilty”. This research would like to contribute to this discussion. Aim is to view the person who is responsible or at least a partial liable for the “unintended” death or serious injury of others: How is the person “dealing” with this experience? Qualitative data from *N*=25 “causer” of the death or severe damage of others were collected. Their stories were acquired through narrative interviews (Hermanns, 1995) and collected data approached by a combination of Grounded Theory (Strauss & Corbin, 1996) as well as qualitative content analysis according to Mayring (2002). Results show a huge variety of different variables: Posttraumatic reactions, shattered assumptions, suicidal thoughts, and different types of guilt feelings. Expectations to get punished by “life” itself were found. Psychodynamic defense and different styles of attribution, which leads even to degradation of victims, is another outcome. Taking responsibility leads for some to acceptance of the situation, for others to not being able to “overcome”. Actions of compensation and reconciliation are actively approached by “causers”. Beneficial versus failed processes of communication between “causer” and victim are shown.

### Beyond Posttraumatic Stress Disorder: posttraumatic growth in children in the aftermath of the Indian Ocean tsunami 16:00–16:15

#### S. Exenberger^1^ and B. Juen^2^

^1^Research Department, SOS Children's Village International, Innsbruck, Austria; ^2^Department of Psychology, University of Innsbruck, Innsbruck, Austria

In the worst hit part of India, the Southern state Tamil Nadu, mostly fishing families were affected by the Indian Ocean tsunami in 2004. This paper presents one work-package of the project “Post-tsunami” funded by the European Commission and goes beyond posttraumatic stress disorder as a main reaction to a traumatic event. It aims to show subjective positive change in children in the long-term aftermath of tsunami, which has been named as posttraumatic growth (PTG) (Tedeschi and Callhoun, 1995, 2004). In 2009, 175 tsunami-affected children aged 8 to 17 gave answers to the Revised Posttraumatic Growth Inventory for Children (PTGI-C-R) and the Children's Impact of Event Scale-13 (CRIES-13). They are single and double orphans, either living with their biological parent or in an out-of-home care organization providing family based care (SOS Children's Village). In addition, 41 children living at a SOS Children's Village gave answers to the open-ended questions of the PTGI-C-R. The quantitative data were analyzed using PASW Statistics 18 software. The qualitative data were analyzed on the basis of the qualitative research methodology “Grounded Theory”. Results indicated that posttraumatic growth is present in children belonging to an Asian culture as 55.5% of them had an average response of some perceived change four years post-event. Older children showed significantly higher PTG scores than younger ones, and no sex differences were found. The total CRIES-13 score correlated significantly with the total PTG score (*r*=.18*, p<.05). About 25 children who gave answers to the open-ended questions described spontaneously positive changes. The results are discussed from an evolutionary and cultural perspective taking into consideration that the hallmark behaviours of PTSD are all adaptive behaviours to extreme threats which can become pathological, but also can be viewed as the foundation of PTG.

### Absence of corpse as a risk factor for complicated grief: the case of the tragedy of Entre-os-Rios, Portugal 16:15–16:30

#### L. Ferreira, V. Almeida and J. Rocha Instituto Superior de Ciências da Saúde- Norte, Cespu—Gandra, Portugal

The loss of a loved one with whom we develop bonds is a source of suffering, pain and despair. When the loss is associated with multiple losses and the absence of the dead bodies of those who died, we can considerer as a situation with high risk for complicated grief. That happened in Entre-os-Rios Portuguese village when a bridge fell on Douro River, all 59 passengers from one bus and three cars died and 36 bodies have nor been recovered. This study aims to reveal several dimensions of the grief process of relatives from the tragedy of Entre-os-Rios victims, 10 years after the tragedy, comparing with grievers from road accidents.To this end, we performed a cross-sectional comparative and exploratory study with a sample of relatives of victims of the tragedy of the Entre-Rios (n=20) in which at least one dead body was not recovered (experimental group) and a sample of relatives of victims of road traffic accidents (n=20), with the same time from bereavement (control group). Were used the Impact of Event Scale—Revised, the Inventory of Complicated Grief and a semi structured interview.There was the prevalence of complicated grief in relatives of victims of the tragedy of Entre-os-Rios (ICG≥25) in 95% and a prevalence of Traumatic Stress (IES-R> 35) of 70%. The difference between groups, experimental and control, was statistically significant using the t-test. We can once again conclude that the absence of body is an important factor in the process of complicated and traumatic grief.

## Open Papers: Cognition and emotion III

### Defensive character of posttraumatic growth: effects of mortality reminders on reports of growth among patients with life-threatening illness and their caregivers 16:45–17:00

#### A. Luszczynska^1^, A. Bukowska-Durawa^1^ and N. Knoll^2,3^

^1^Warsaw School of Social Sciences and Humanities, Warsaw, Poland; ^2^Institute of Medical Psychology, Charité, Universitätsmedizin Berlin, Berlin, Germany; ^3^Department of Psychology, Freie Universität Berlin, Berlin, Germany

Individuals confronted with a life-threatening illness often report posttraumatic growth or finding benefits in disease. These positive evaluations of personal strength, perceptions of improved personal relations and new possibilities may represent a defensive response (cf. Janus-face model). Three studies investigated the effects of mortality reminders on reports of posttraumatic growth or benefit findings among people living with life-threatening illness or their caregivers. Eighty people living with HIV (Study 1), 164 breast cancer survivors (Study 2), and 50 family caregivers for a patient with Huntington Disease (Study 3) were randomly assigned to the experimental (mortality reminders) or control conditions. Across three studies, those exposed to mortality reminders reported lower posttraumatic growth or benefit finding, compared to the controls. These effects were moderated by time elapsed since diagnosis: mortality reminders led to lower posttraumatic growth/benefit finding among those who received the diagnosis more recently. The results provide an insight into the defensive character of posttraumatic growth/finding benefits in illness and changes in the character of these beliefs over time elapsing since diagnosis.

### Childhood trauma and PTSD increase the risk of cognitive impairment in a sample of former indentured child laborers in old age 17:00–17:15

#### A. Burri, A. Maercker, S. Krammer and K. Simmen-Janevska Department of Psychology, University of Zurich, Zurich, Switzerland

*Background*: Recently, links between trauma, posttraumatic stress disorder (PTSD) and increased risk of dementia have been suggested. According to several pieces of evidence, stress experienced early in life induces structural, functional, and epigenetic changes in brain regions involved in cognition. *Aim*: To investigate the association between childhood trauma exposure, PTSD and neurocognitive function in a unique cohort of former indentured Swiss child laborers in their late adulthood. To the best of our knowledge this is the first study to investigate the relative role of childhood versus adulthood trauma in the risk of cognitive impairment in later life. *Material and methods*: According to PTSD status and whether they experienced childhood trauma (CT) or adulthood trauma (AT), participants (n=96) were categorized as belonging to one of four groups: CT/PTSD+, CT/PTSD-, AT/PTSD+, AT/PTSD-. Information on cognitive status was assessed using the Structured Interview for Diagnosis of Dementia of Alzheimer Type, Multi-infarct Dementia and Dementia of other Etiology according to ICD-10 and DSM-III-R, the Mini-Mental State Examination, and a vocabulary test. Depression symptoms were investigated as a potential mediator for neurocognitive functioning. *Results*: Individuals screening positively for PTSD performed worse on all cognitive tasks compared to healthy individuals, independent of whether or not they reported childhood adversity. When controlling for depression symptoms, the relationship between PTSD and cognitive impairment became stronger. *Conclusion*: Overall, results tentatively indicate that PTSD is accompanied by cognitive deficits which appear to be independent of earlier childhood adversity. Our findings suggest that cognitive deficits in old age may be partly a consequence of PTSD or at least be aggravated by it. Consideration of cognitive deficits when treating PTSD patients and victims of lifespan trauma without diagnose of a psychiatric condition is crucial. Furthermore, early intervention may prevent long-term deficits in memory function.

### The role of EEG data in guiding treatment and evaluating treatment outcome of refugees affected by torture and war trauma 17:15–17:30

#### J. Aroche NSW Service for the Treatment and Rehabilitation of Torture and Trauma Survivors (STARTTS), Sydney, Australia

Clients seeking treatment at STARTTS tend to exhibit complex presentations that often include Post Traumatic Stress Disorder (PTSD) as well as a complex array of other symptoms. The treatment available at STARTTS includes approaches with a substantial evidence base, such as Eye Movement Desensitisation and Reprocessing (EMDR) and exposure based Cognitive Behavioural Therapy (CBT), as well as other less well researched approaches, such as Neurofeedback Therapy (EEG Biofeedback). It is also often complemented with a variety of other psychosocial and body based interventions. One of the challenges inherent to this model is establishing a coherent basis for making decisions about the optimum choice of treatment for different clients presenting to the service. Increasingly, STARTTS has been utilizing Electro EncephaloGraphy (EEG) data to assist in making these decisions, guide treatment and evaluate results for the more severely affected client cohort. This paper will outline how EEG data is being utilized to complement clinical observations and other assessment methods, and provide examples from case studies to illustrate the encouraging results obtained.

### Post-traumatic stress disorder and allocentric spatial memory 17:30–17:45

#### K. Smith^1^, N. Burgess^2^, C. Brewin^3^ and J. King^3^

^1^Royal Holloway; ^2^Institute of Cognitive Neuroscience; ^3^University College, London, UK

*Objectives*: This study will investigate the effect of stress on hippocampal functioning, following trauma, and examine whether PTSD sufferers have impaired hippocampal functioning as suggested by a prominent model for PTSD: Brewin's (1996, 2001) Dual Representation Theory. The hippocampus integrates perceptual and memory systems in a way that means that the hippocampus is uniquely central to memory for spatial location and navigation, and so it is reasonable to expect that it would be involved in contextual fear conditioning. The effect of PTSD on the hippocampus will be investigated by assessing performance on two tasks of allocentric spatial memory compared with trauma-exposed controls. *Design*: This study will compare the performance of a group of PTSD sufferers and a trauma-exposed control group on two tests of allocentric spatial memory. A higher total score reflects greater hippocampal functioning. The independent variables will therefore be the PDS total score and the total score on the BDI-II. *Methods*: Participants will complete two Subtests on the “four mountains” task (topographical perception and topographical memory) (Hartley et al., 2007) and two Subtests of the “town square” task (same viewpoint and shifted viewpoint) (King, 2002). *Results*: Participants in the PTSD group performed worse on two tests of allocentric spatial memory than trauma-exposed controls. There was no difference between groups on egocentric memory performance (same viewpoint condition on the town square task). Performance will correlated with PTSD symptom severity on the PDS and symptoms severity on the BDI-II. *Conclusion*: Impaired or reduced hippocampal processing is implicated in PTSD.

### The role of propranolol in reducing emotional memory consolidation and reconsolidation in healthy individuals and symptoms of posttraumatic stress disorder in patients: a systematic review 17:45–18:00

#### L. Olivera Figueroa^1^, M. Lonergan^2^ and A. Brunet^3^

^1^Department of Psychiatry, Yale University School of Medicine; ^2^Department of Psychology, Concordia University, Montréal, Québec, Canada; ^3^Psychosocial Research Division, Douglas Mental Health University Institute, Montréal, Québec, Canada

*Rationale:* A growing body of animal and human research suggests that the beta-blocker propranolol has the capacity of selectively affecting emotional memory consolidation and reconsolidation for negative stimuli. These findings have inspired researchers to assess Propranolol's potential to serve as a pharmacological adjunct for the treatment of memory-based psychiatric conditions like posttraumatic stress disorder (PTSD). However, studies on this phenomenon have yielded incongruent findings. *Objective:* We evaluated whether administration of propranolol, compared to placebo, can interfere with the consolidation and reconsolidation of emotional memories in healthy populations, as well as with symptoms of PTSD. *Methods*: A systematic search for randomized control trial articles published in English, French, and Spanish-language journals after January 1994 until March 2011 was conducted across the PubMed, PsycInfo, ISI Web of Knowledge, PILOTS, and Cochrane Central databases. To assess the quality of each study, the Jadad Scale was appraised. *Results*: Our systematic search revealed 15 studies examining the effect of propranolol on consolidation and 7 studies examining reconsolidation of emotional memory in healthy populations. Furthermore, 7 articles were found addressing propranolol's role in treating PTSD. *Conclusions*: After a qualitative analysis of each selected study, some support was found for the notion that propranolol may impair the consolidation and reconsolidation of emotional memories. Moreover, the overviewed literature showed preliminary support for the potential of propranolol in the treatment of PTSD. However, further research is necessary before forming firm conclusions on these topics, particularly on whether propranolol has the capacity to affect the reconsolidation of previously-formed traumatic memories.

## ORAL, JUNE 8 HALL GLORIA

## Morning Responding to disasters
*Workshop: Childhood trauma reactions and the role of teachers and schools post-natural disaster - Training the trainer*

### Childhood trauma reactions and the role of teachers and schools post-natural disaster: training the trainer 10:00–10:20

#### R. Le Brocque^1^, J. Kenardy^1^, A. De Young^1^, S. March^1^ and N. Triggell^2^

^1^School of Medicine, University of Queensland, Brisbane, Australia; ^2^Education Queensland, Brisbane, Australia

In February 2009, the Black Saturday bushfires tore across the Australian state of Victoria and resulted in Australia's highest ever loss of life from a bushfire: 173 people died and 414 were injured. The fires burned over 4,500 km2 (450,000 hectares, 1.1 million acres). In December, 2010 and January, 2011, a series of floods hit Australia, primarily the state of Queensland. The floods killed 35 people. At least 70 towns and over 200,000 people were affected. The area affected covered over 1.35 million square kilometers (half a million square miles). Three-quarters of the state of Queensland was declared a disaster zone. In response to these events which affected many children, we developed a training and information package for teachers and school based mental health professionals, ‘Childhood Trauma Reactions: A guide for Teachers from Pre-school to Year 12’. The training was delivered across the state of Queensland and positive evaluations relating to content, delivery, and accessibility were received from both child health specialists and school personnel. Teachers are in a unique position to identify children experiencing difficulties following natural disasters because of their role, expertise, and extended contact with children. This resource package is designed to assist teachers and health professionals in becoming more attuned to identifying emotional and behavioral difficulties in young people following a traumatic event and provides information on the prevention and management of long-term adverse reactions. This workshop, in a 3–4 hour training format, will provide an introduction to the program which is freely available on our web. In this workshop we will overview child trauma reactions across development including very young children to adolescents, explore the role of teachers and schools in helping children after traumatic events such as natural disasters, and discuss strategies for identifying children who are in need of more specialized psychosocial support.

### The role of teachers and child mental health specialists in helping children after natural disaster 10:20–10:40

#### R. Le Brocque^1^, J. Kenardy^1^, A. De Young^1^, S. March^1^ and N. Triggell^2^

^1^School of Medicine, University of Queensland, Brisbane, Australia; ^2^Education Queensland, Brisbane, Australia

Research suggests that there is a range of ways that schools respond following natural disasters. Some schools decide to promote and establish a normal routine as soon as possible. This is often the case for high schools where there is immense pressure for the young adults to perform in end of year university entrance examinations. However, for a variety of reasons, other schools may also choose to re-establish normal routines. Despite the preference for normal routines, these schools may often experience the intrusion of disaster related content in the children's work, find that students are unable to concentrate for long periods, and find that academic functioning may not be optimal. Alternatively, some schools provide counseling and support for students and their families and often find the role of teacher blurred within the post disaster environment. Some schools incorporate disaster planning and knowledge into their curriculum in the form of content on environmental recovery or projects on understanding weather patterns and weather events. Psychosocial skills, such as resilience and anti-bullying projects, are also increasingly being integrated into the school curriculum and these coping skills can be used in the post-disaster environment. Teachers often ask how they can help young people in their class who have experienced a traumatic event. Although teachers play an important role in identifying mental health concerns in their students, their primary role is in continuing and supporting children's education. This workshop explores how schools and teachers can help their students in the acute and long term following natural disasters. The workshop also explores how teachers and child mental health specialists can work together to provide psychosocial support for the young people in their care in the wake of a natural disaster.

### Childhood trauma reactions: a guide for teachers from preschool to year 12. Integrating lessons learned from a disaster recovery program for children aimed at teachers and mental health professionals 10:40–11:00

#### R. Le Brocque^1^, J. Kenardy^1^, A. De Young^1^, S. March^1^ and N. Triggell^2^

^1^School of Medicine, University of Queensland, Brisbane, Australia;^2^Education Queensland, Brisbane, Australia

In response to a series of natural disasters occurring in Australia in 2009 to 2011 a training and information package for teachers and school based mental health professionals was developed, ‘Childhood Trauma Reactions: A guide for Teachers from Pre-school to Year 12’. The training was delivered across the state of Queensland and positive evaluations relating to content, delivery, and accessibility were received from both child health specialists and school personnel. Teachers are in a unique position to identify children experiencing difficulties following natural disasters because of their role, expertise, and extended contact with children. This resource package is designed to assist teachers and health professionals in becoming more attuned to identifying emotional and behavioral difficulties in young people following a traumatic event and provides information on the prevention and management of long-term adverse reactions. This session presents a summary of the information package and training delivered to teachers and mental health professionals and highlights how one state responded to the emerging mental health needs of children post-disaster. Participants in this session will discuss the challenges of screening and identifying at-risk children in the immediate and short term following disaster. Workshop participants will also identify resources within their own community which can be accessed to help support children and adolescents post disaster.

## Responding to disasters
*Symposium: Optimal psychosocial care concerning shocking incidents. Experiences and evaluation part I*

### Evaluating post-disaster psychosocial care using a multi-dimensional heuristic framework: lessons for research and practice 11:45–12:05

#### M. Duckers^1^ and J. Yzermans^2^

^1^Impact, Diemen and ^2^Nivel, Utrecht, The Netherlands

Psychosocial care provided to those affected by disasters and critical incidents has received increased attention in recent years in international literature. The topic covers all disaster-related problems, falling within the scope of a variety of service providers. However, how these actors should function in this context, is not always easy to determine. Models to support decision-making are welcome, as optimal care intervention is debated, and empirical evidence is scarce. In order to gain a better understanding of post-disaster psychosocial care a framework is constructed, comprised of three dimensions derived from the international literature and several practice guidelines. Psychosocial care can differ in (1) ‘objective’, varying between support and clinical care; (2) ‘attitude’, between waiting and outreaching; and (3) ‘quality’, from low to high. The dimensions constitute a three-dimensional parabolic distribution area where care provision can be positioned. Theoretically, attempts to prevent an overly passive or active attitude towards those affected by disasters are to be promoted, regardless of the objective. Sensitivity to people's needs, vulnerabilities and adaptive capacity (resilience) can potentially contribute to quality. This framework is used to evaluate different disasters and crises in the Netherlands. Lessons for the future planning of psychosocial care and research into it are presented.

### Validation of a screening instrument for victims of a crash with Turkish airlines in 2009 12:05–12:25

#### J. Gouweloos^1^, J. Ten Brinke^2^, M. Sijbrandij^3^ and H. Te Brake^1^

^1^Impact, Diemen, The Netherlands; ^2^GGD Kennemerland, Hoofddorp, The Netherlands; ^3^VU University Amsterdam, The Netherlands

After a disaster, an important minority develops long-term mental health problems (most commonly posttraumatic stress disorder, other anxiety disorders or depression). In order to provide optimal psychosocial care, it is important to identify those who are at risk of mental health problems and in need for psychological treatment at an early stage. After the crash with Turkish Airlines at Schiphol Airport (the Netherlands) in 2009, Impact developed a short screening instrument: “Trauma Checklist Getroffenen” (TCG, Trauma Checklist for Affected). The instrument measured the psychosocial health and treatment needs of the victims. It is based on the methods used after the London bombings in 2005 (Brewin, 2008). This presentation describes the accuracy of the TCG in predicting mental health problems. It will also highlight the treatment needs of the victims of the crash. Interviewers of the local public health organization called the victims 6 weeks (N=89), 9 months (N=84) and 3 years (N=56) after the crash. They completed the TCG by telephone. To measure the sensitivity and specificity of the TCG respondents also completed a structured clinical interview, the M.I.N.I. International Neuropsychiatric Interview (Sheehan et al., 1998), 3 years after the crash. The last interviews were conducted in December 2012. This presentation will describe how accurate the TCG can predict PTSD, depression and flight phobia 3 years after an airplane crash. It will also compare the treatment needs of victims in the first year and 3 years after the crash. This will shed light on the questions if the needs of the victims have been met, if those who are mostly in need for professional help are reached and if an active, outreaching approach can be recommended.

### A fine balance. How can we supply necessary support and outreach services and at the same time avoid interfering with survivors own coping efforts? 12:25–12:45

#### G. Dyb^1^, T. Jensen^1^, K. Alve Glad^1^, E. Nygaard^2^, S. Thoresen^1^

^1^Norwegian Centre for Violence and Traumatic Stress Studies, Oslo, Norway; ^2^University of Oslo, Oslo, Norway

On July 22nd 2011, Norway experienced a terror attack at an island summer camp for young members of the Labor Party. 69 youth died and 490 survived. A national intervention program included; early and proactive outreach from crises teams, continuity in outreach from a contact person in the municipalities and targeted interventions by specialized mental health services to survivors and families in need. To aid identifying people with clinical needs a screening instrument was developed, and recommended to be performed at three time points. The intent was to ensure that all survivors in need for services were identified and offered relevant attention. Survivors from the Utøya Island (N=325, response rate=66%) were interviewed 5 months after the shooting. Trauma exposure, loss of someone close, peritraumatic reactions and post-traumatic stress reactions were measured. Provided services were registered including perceived usefulness of care. Trauma exposure was very high in the survivors, e.g. 64% witnessed someone get injured or killed, and 87% reported to have seen dead people. The interviews indicated that survivors had received services from crises teams in the acute phase (87%) that 84% had a contact person in the municipalities. Specialized mental health services were often involved (73.1%). Twenty survivors (6.2%) had not received any help. Perceived usefulness of services will be presented in relation to trauma experiences and levels of distress. A high level of exposure is a risk factor of long lasting suffering after trauma, and proactive outreach and early involvement of specialized services may have promoted healing. However, proactivity may influence survivors’ coping strategies negatively. Youth's perceived usefulness of services can shed light on these difficult considerations.

## Afternoon Evidence-based practice on trauma
*Symposium: How to prevent PTSD - explorations of novel interventions early after trauma*

### Intranasal oxytocin administration to prevent PTSD in recently traumatized individuals: the rationale and design of a randomized controlled trial 15:15–15:30

#### J. Frijling^1^, M. Van Zuiden^1^, L. Nawijn^1^, S. Koch^1^, D. Veltman^2^ and M. Olff^1^

^1^Department of Psychiatry, Academic Medical Center, University of Amsterdam, Amsterdam, The Netherlands; ^2^Department of Psychiatry, VU University Medical Center, Amsterdam, The Netherlands

Currently there are no evidence-based interventions to be administered shortly after trauma exposure that prevent the development of post-traumatic stress disorder (PTSD) in traumatized individuals. The neuropeptide oxytocin is a potent regulator of several important processes associated with PTSD development. Oxytocin regulates physiological and behavioral stress and fear responses. In addition, oxytocin administration influences socio-emotional processes such as attachment behaviors. Interestingly, dysregulated stress and fear responses prior to and immediately post-trauma, as well as a lack of perceived social support early after trauma are risk factors for the development of PTSD. Therefore, we hypothesize that early oxytocin administration may be a promising strategy for the prevention of PTSD, by ameliorating dysregulated stress and fear responses as well as facilitating adaptive social functioning. We have initiated a randomized double blind placebo-controlled trial to investigate the effectiveness of an intranasal oxytocin treatment regimen in preventing the development of PTSD in recently traumatized individuals at increased risk for PTSD. Participants at risk for PTSD are recruited from Emergency Departments of two medical centers in Amsterdam. The one-week intranasal oxytocin/placebo treatment starts at the latest at day ten post-trauma. Participants are assessed for PTSD symptoms at 1.5, 3 and 6 months post-trauma. In this presentation, the rationale behind intranasal stimulation of the oxytocin system in recently trauma-exposed individuals at risk for PTSD will be discussed and the design of the trial will be presented. In addition, preliminary data of the trial will be shown.

### Efficacy of a brief dyadic cognitive behavioral intervention designed to prevent PTSD 15:30–15:45

#### A. Brunet^1^, M. J. Cordova^2^, I. Bousquet Des Groseilliers^3^, A. Marchand^3^ and J. I. Ruzek^4^

^1^McGill University; ^2^VA Northern California Health Care System, CA, USA; ^3^Université du Québec à Montreal, Canada; ^4^NC-PTSD Palo Alto, CA, USA

*Background*: Posttraumatic Stress Disorder is a public health problem for which there is a dearth of effective secondary prevention interventions. *Design*: In a randomized-controlled trial, we evaluated the efficacy of a new dyadic two-session cognitive-behavioral intervention vs. a wait-list, delivered by a trained social worker or a nurse. Participants were adults presenting to the emergency department in the aftermath of trauma exposure, mostly motor vehicle accidents. *Results*: In the intent-to-treat sample (N=74), we obtained a significant time by group interaction whereby the treated group was more improved at 3-month follow-up than the non-treated group, F(2, 72)=4.17, p=.019. Controlling for the moderate improvement observed in the wait-list group, the intervention yielded a net effect size of d=0.42. Similar results were obtained with the sample of study completers at the 3-month follow-up (F[2, 64]=3.30, p=.043; d=.43), as well as at the 2-year follow-up (F[3, 46]=4.11, p=0.008; d=.54). At follow-up, no treated participant met criteria for PTSD, compared to five in the control group (Fisher's exact test, p=0.01). From a public health perspective, the occurrence and severity of PTSD could be reduced if this new, easy to deliver, intervention was offered as part of regular care in the hospital setting.

### Results of randomized controlled trial of a Web-based early intervention for posttraumatic stress disorder: moving from universal to selective prevention 15:45–16:00

#### J. Mouthaan^1^, M. Sijbrandij^2^ and M. Olff^1^

^1^Department of Psychiatry, Academic Medical Center, University of Amsterdam, Amsterdam, The Netherlands; ^2^Clinical Psychology, VU University, Amsterdam, The Netherlands

In this presentation, we will present the results of a randomized controlled trial of a self-guided internet-based intervention (called Trauma TIPS) to prevent the onset of PTSD. Adult injury patients were randomly assigned to receive the Trauma TIPS internet intervention (*n=*151) or to no early intervention (*n=*149). Trauma TIPS consisted of psychoeducation, in vivo exposure and stress management techniques. Clinically assessed and self-reported PTSD symptom severity was measured at 1, 3, 6 and 12 months post-injury. The results showed that in both the intervention and control group PTSD symptoms significantly decreased over time (*P<*.001) without significant differences in trend. Results from post-hoc subgroup analyses indicated that the Trauma TIPS intervention may be efficacious in reducing PTSD symptoms in patients with high initial PTSD symptoms. In light of our results, future research should move from universal to selective prevention and focus on the efficacy of applying interventions to high risk individuals. Early risk screening strategies using highly sensitive instruments can facilitate in identifying persons at risk for developing PTSD in need of diagnostic follow-up.

### Coping coach: Web-based secondary prevention for school-age children 16:00–16:15

#### N. Kassam-Adams^1^, M. Marsac^1^, K. Kohser^1^, J. Kenardy^2^, S. March^3^ and F. Winston^1^

^1^The Children's Hospital of Philadelphia, Philadelphia, PA; ^2^University of Queensland, Australia; ^3^University of Southern Queensland, Australia

*Background*: The Internet can expand access to secondary prevention resources for a wide range of trauma-exposed children and families. Web-based interventions (alone or in combination with professional care) may play a key role in stepped care models for prevention and early intervention after acute trauma. This presentation will describe the development and initial evaluation of Coping Coach, a web-based prevention tool for school-aged children with recent acute trauma. *Method*: Based in the empirical research literature, we identified likely etiological mechanisms for the persistence of posttraumatic stress (PTS) in children after acute trauma. We then developed “Coping Coach,” an interactive online game that addresses trauma-related appraisals and specific coping strategies with the aim of reducing the development of PTS symptoms. We employed systematic user-testing with children to optimize usability and functionality of key interactive features. 12 trauma experts were asked to review Coping Coach content and activities to assess content validity and developmental appropriateness. *Results*: Preliminary results from expert review of Coping Coach indicate ratings of good to excellent for the relevance, likely effectiveness, and age-appropriateness of game activities. In the US, pilot-testing with children exposed to acute medical events found high acceptability and engagement with intervention activities. Pilot testing with children in Australia will begin soon. *Discussion*: Best practices in the development of web-based interventions and “serious games” require theoretically-grounded selection of intervention targets and interactive activities. Results of user testing and expert review indicate that Coping Coach is very engaging for children, and that the activities address the intended targets. Further testing in a randomized, controlled trial is now underway to evaluate the effect of Coping Coach on intervention targets (appraisals, coping) and to estimate its efficacy in reducing PTS symptoms and improving health-related quality of life after acute medical events.

## Responding to disasters
*Symposium: Optimal psychosocial care concerning shocking incidents. Experiences and evaluation part II*

### Toward optimal psychosocial care concerning shocking events: resilience, timely detection, and adequate referral 16:45–17:05

#### H. Te Brake
^1^Impact, Diemen, The Netherlands

Although there is agreement that most people recover on their own merit following shocking events, an overarching view on how this resilient stance relates to care for trauma victims needs further development. Given the wide variety of caregivers who provide psychosocial care in the early phase (both professionals from a clinical stance and non-professionals, e.g. volunteers from a supportive stance), such an overarching view would be needed to achieve optimal cooperation and provide the best possible care. Three questions are relevant: (1) how do we support people's resilience; (2) how (and when) do we detect the people in acute need; (3) how do we organize a timely referral to high quality treatment? However, the link between all three questions is of great importance and strongly relates to individual resilience. Based on qualitative and quantitative research on resilience, guideline development, and expert opinions, an evidence informed approach is used to address these issues. Vantage point is that optimal quality of psychosocial care makes maximal use of the victim's resilience.

### Evaluating quality of post-disaster psychosocial care from a victim's perspective 17:05–17:25

#### J. Holsappel^1^, T. Dorn^2^, T. Fassaert^2^ and H. Te Brake^1^

^1^Impact, Diemen , The Netherlands; ^2^GGD Amsterdam , The Netherlands

To measure the experienced psychosocial care following critical incidents, two important conditions should be met: 1) agreement on the features of optimal post-disaster psychosocial support; and 2) availability of an instrument to assess the experienced by victims. Both conditions are covered in the research project *Quality of Psychosocial Care (QPC)*. Consensus on features of good psychosocial care was found using the method of concept mapping. With a group consisting of both experts and victims, an extensive list of characteristics of optimal psychosocial care was made, which was then analyzed (e.g. in using cluster analyses). The analysis resulted in the formulation of eight principles of optimal psychosocial care, varying from ‘paying attention to the needs and abilities of the victims’ to ‘informing the victims on the emotions they can expect’. Based on these clusters, a questionnaire was designed, to be used about six to eight weeks after a critical incident. The questionnaire comprises thirty items, which victims have to assess for weight (how important is the specific item) and frequency (how often was the item realized in the contacts with caregivers). The presentation will present preliminary psychometric results of a pilot testing of the questionnaire among victims of a train accident in the Netherlands (N>130) which is currently taking place. Also, some specific issues will be discussed, like how to develop a questionnaire that can be applied in diverse situations and at the same time generates sufficiently specific results so they can lead to action and change. Reflecting on the value of expert opinions for the design of a measuring instrument, on its action ability, and on the extent to which the questionnaire specifically Dutch, it will be discussed how this instrument could bring quality management in psychosocial care any further.

### Monitoring the implementation of psychosocial care guidelines in uniformed service organizations 17:25–17:45

#### N. Burger, M. Duckers and F. Zwenk Impact, Diemen, The Netherlands

In recent years several instruments have been developed to organize psychosocial support in uniformed service organizations (USOs). Standards and guidelines are a valuable point of reference, however, there is a traditional gap between norms described in guidelines and everyday practice. Once the nature of the gap—large or small—is explored, one can undertake deliberate action to close it. Against this background a national monitor is conducted in the Netherlands to determine the extent to which USOs work in line with the Guidelines Psychosocial Support for Uniformed Workers. Police officers, firemen, paramedics and military staff run the risk of being confronted with potentially shocking events. The presentation will focus on the results from a combined qualitative and quantitative study. It is explored systematically whether their organization includes structure and process features as recommended in the guidelines, as well as the factors that stimulate or hinder guideline implementation. Particular attention is given to explanatory variables like organizational support; team organization; and external change agency support. Differences between types of USOs are identified. The monitoring study appears a useful implementation tool for organizations where professionals are indispensable guards of each other's well-being and health.

## ORAL, JUNE 8 HALL LADY G

## Morning Cultural issues and trauma
*Workshop: Pathways to healing of patients suffering from spirit possession*

### Pathways to healing of patients suffering from spirit possession 10:00–11:00

#### M. Van Duijl Netherlands Institute of Forensic Psychiatry, The Hague, The Netherlands

Refugees and migrants seeking mental health services often present with dissociative presentations and cultural explanations that psychologists and psychiatrists treating them find difficult to understand and deal with. Western diagnostic categories and treatment models seem limited in dealing with this in a transcultural setting. There is however increasing evidence for dissociative presentations, such as dissociative and possessive trance disorders, being related to traumatic experiences [1]. This workshop will give more insight in recognition, diagnosis and management of dissociative disorders in the transcultural practice. The applicability of the new diagnostic criteria for dissociative disorders in the DSM-5 will be discussed and practiced in the workshop. Case histories from the African and Dutch clinical setting with traumatized refugees and migrants will be discussed to illustrate different idioms of distress, explanatory models and culturally sensitive interventions. Presenters’ most recent research findings on classification [2], help-seeking and explanatory models of patients with spirit possession in Uganda will be referred to, as well as recent relevant literature. Aims of the workshop: The participants will achieve tools to recognize dissociative symptoms, learn how to deal with different explanatory models for dissociation and reflect on options for management of dissociative disorders in a transcultural context.

## Impact of trauma on communities
*Symposium: Positive and negative effects of war trauma on Iraqi civilians*

### Psychological problems related to war trauma in Iraqi civilians 11:45–12:05

#### M. M. Koryurek
^1^State hospital, Ankara, Turkey

Most studies on war-related psychological trauma have focused on posttraumatic stress disorder (PTSD) in soldiers returning home after deployment. Yet, the number of civilians affected by wars is rising. Following the invasion of Iraq by USA between 2003 and 2006, approximately four million Iraqis were displaced, including Turkmens, who are the third largest ethnic group in Iraq after Arabs and Kurds. Because of their cultural links with Turkey, many young Iraqi Turkmen pursue higher education in Turkey. This paper aims to detect the prevalence and predictors of psychological symptoms caused by war trauma in young Iraqi Turkmen students who came to Ankara for higher education. The sample consists of 203 Iraqi Turkmen students who were surveyed in a group setting using standardized instruments to measure depression and PTSD. Results show that the prevalence of PTSD and depression was 17.2% (21.2% for females, 16.3% for males) and 23.2% (20.0% for females, 23.9% for males), respectively. The most frequent traumas reported was waking up by the noise of an explosion or bombing, being at the scene during an explosion or bombing event and house being raided. Being at the scene during an explosion or bombing event was more frequent in men than women. Our results show that significant rates of war-related psychopathology (PTSD and depression) persist many years after the trauma among Iraqi youth, despite the fact that they are safely away from the war zone.

### Trauma types and posttraumatic growth in war-exposed Iraqi youth 12:05–12:25

#### C. Kilic Department of Psychiatry, Hacettepe University, Ankara, Turkey

Although traumatic events cause long-lasting psychological symptoms, many survivors may come out stronger. Benefiting in some way from the traumatic experience is called *posttraumatic growth* (PTG). Type of the trauma and personality seem to be important predictors of development of growth after traumas. Several possibilities exist to explain the differential effects of trauma type on the development of PTG, including severity of trauma, the degree of social network disruption, stigmatizing or shame-inducing nature of trauma, etc. Turkmens are the third largest ethnic group in Iraq and have suffered the burden of war, as well as ethnic and sectarian violence. The purpose of this study was to investigate the relationship between the traumatic experiences and PTG among Turkmen youth living in Ankara. We hypothesized that different trauma types would lead to differing levels of growth. A total of 203 students were included in the study. All were ethnic Turkmen university students living in Ankara. 163 (80.3%) were male. Mean age of the sample was 25.2 (sd: 3.8) (range 18–36). The students were assessed using self-report measures of PTSD, depression and PTGI. They were also asked about the details of war trauma. Regression analyses were conducted where the independent predictors of PTG scores were examined. Four different trauma types were included in analyses in addition to demographic variables. Growth in the personal domain was negatively predicted by personal trauma factor (i.e. higher personal trauma meant lower personal growth). Non-personal growth was not predicted by the study variables.

### Relationship of posttraumatic growth to symptoms of PTSD in young Iraqis studying in Turkey 12:25–12:45

#### K. M. Magruder Medical University of South Carolina, USA

While the scientific literature is replete with reports of the negative effects of war on both combatants’ and civilians’ mental health, there is considerably less concerning the positive effects. The concept of posttraumatic grown (PTG) captures the notion of positive psychological gains as a result of struggles with adversity. These are particularly important issues for young people who have grown up in war-torn areas. Iraq is a country that has been particularly hard hit, with almost continual war and civil unrest since 1980. The aim of this paper is to examine the experiences of 203 young Iraqi students studying in Turkish universities who grew up in Iraq during a period of 3 wars and investigate their levels of PTG and the relationship of PTG with symptoms of posttraumatic stress and depression. Eligible Iraqi students were administered the Posttraumatic Growth Inventory (PTGI), the Traumatic Stress Symptom Checklist (TSSC), the Beck Depression Inventory, a war and life experiences assessment, and socio-demographic information. There were no differences in total PTGI scores by age, gender, or depression score. However, PTGI scores were significantly higher (i.e., more growth) for those with TSSC scores suggestive of PTSD. Multivariate analyses showed that PTGI score, war-related traumatic events (post 2003), general life events (post 2003), and depression scores were positively related to TSSC score. Results show that higher levels of post-traumatic growth are related to higher levels of PTSD symptoms, even controlling for depression, trauma, and other life events. These findings suggest that in addition to adversity itself, the presence of PTSD symptoms may stimulate PTG, and that depression symptoms do not dampen this response.

## Afternoon Cultural issues and trauma
*Workshop: Local work with complex traumatized refugee families in Norway*

### Local work with complex traumatized refugee families in Norway 15:15–15:45

#### K. Jagmann, M. Braein and K. Holt The Regional Centre for Prevention of Violence, Trauma and Suicide, Region East, Norway

We are three clinicians working as special advices at The Regional Centre for Prevention of Violence, Trauma and Suicide, Region East (RVTS Øst). RVTS Øst is located at Oslo University Hospital and is financed by The Norwegian Directorate of Health. Our Centre covers almost half of the population of Norway. RVTS Øst has two main tasks: 1) to increase competence among professionals in public health and other relevant public institutions working with persons affected by trauma and suicide, and 2) to support local and regional cooperation and professional networking. In this workshop we want to share our experiences from our current program for increasing competence among the professionals in the local municipalities on work with complex traumatized refugee families. This program is a training of the local worker in using a model for the family work. This model is sensitive to trauma and culture as well as focusing on the complexity of the family situation. In the workshop we want to present this by using role play, showing video and pictures and share our knowledge through an oral presentation. We also want to create room in the workshop for the sharing of experiences of the workshop participants in similar work.

### A practical approach to enhancing local work with complex traumatized refugee families in Norway 15:45–16:15

#### K. Jagmann, M. Braein and K. Holt
^1^The Regional Centre for Prevention of Violence, Trauma and Suicide, Region East, Norway

Norwegian municipalities settle refugee families based on political decisions, but there is a lack of guidelines on how to help complex traumatized families after being settled in the communities. We want to demonstrate a model for work with refugee families that we have experienced as effective and helpful for complex traumatized families. The model is based on a trauma sensitive, culture sensitive and holistic approach. The children are our particular focus, but we also see the need of giving interventions to the family as a group and for the parents. We want to demonstrate the model through a case presentation of a complex traumatized refugee family we have worked with and followed for several years. The interventions used are trauma sensitive; based on a phase oriented treatment plan where stress reducing interventions and stabilization comes prior to integrating and resolving trauma. The model is also culture sensitive requiring that the service providers are aware of the cultural factors central in the meeting between the clients and us. Further, we believe that working with complex family situations require a holistic approach demanding a systematic long term cooperation between the different services in the community. Flexibility, as well as creativity is important for adjusting interventions to the particular family. By presenting our case example we will demonstrate how this work can be demanding, time consuming and at times frustrating. But we hope we as well will demonstrate the joy, meaningfulness and hope we experienced in the work with these families.

## Effects of trauma on families and children
*Workshop: Body awareness, new possibilities for children and young refugees*

### Body awareness, new possibilities for children and young refugees 16:45–17:45

#### G. Nielsen Oasis Copenhagen Denmark

The workshop describes the development of the body awareness through exercises, games and body therapy with tools from psycho-motor skills and Somatic Experience (developed by Peter Levine Ph. D.) The workshop invites to try out simple exercises from the young people's progress in the body therapy. A workshop on the body therapeutic work, with children and young people in the interdisciplinary treatment with traumatized refugees in Oasis, Copenhagen, Denmark. A little Iraqi girl and a young Afghan woman, both caring daughters of traumatized refugees. Common to them is the continuing concern for their parents. A young Kosovo Albanian man fled unaccompanied from war at the age of 17, when did his escape actually end? The workshop is based on their reports and progress in the therapy. Each represents one of the various defense mechanisms. Common to them is their emerging confidence in their perceptions and their eagerness to find a foothold in their own lives.

## ORAL, JUNE 8 HALL SAVOIA

## Morning Miscellaneous
*Invited symposium: New studies in the military: Evidence in tailored interventions*

### Does structured post-deployment rest (third location decompression) improve the mental health of military personnel? 10:00–10:15

#### N. Greenberg^1^, N. Jones^2^, N. Fear^2^, M. Fertout^3^ and S. Wessely^2^

^1^Academic Centre for Defence Mental Health, King's College London, and Department of Psychological Medicine, Institute of Psychiatry, London, UK; ^2^King's Centre for Military Health Research, King's College London, and Department of Psychological Medicine, Institute of Psychiatry, London, UK; ^3^Academic Centre for Defence Mental Health, Department of Psychological Medicine, Institute of Psychiatry, King's College London, Weston Education Centre, London, UK

*Objective*: Third location decompression (TLD) is an activity undertaken by UK Armed Forces (UK AF) personnel at the end of an operational deployment, which aims to smooth the transition between operations and returning home. We assessed whether TLD impacted upon both mental health and post-deployment re-adjustment. *Method*: Data collected during a large cohort study was examined to identify personnel who either engaged in TLD or returned home directly following deployment. Propensity scores and inverse probability of treatment weights (IPTW) were generated and used in adjusted regression analyses to compare mental health outcomes and post-deployment re-adjustment problems. *Results*: TLD had a positive impact upon mental health outcomes (PTSD and multiple physical symptoms) and levels of harmful alcohol use. However, when the samples were stratified by combat exposure, although post-deployment re-adjustment was similar for all exposure levels, personnel experiencing low and moderate levels of combat exposure experienced the greatest positive mental health effects. *Conclusion*: We found no evidence to suggest that TLD promotes better post-deployment re-adjustment, however, we found a positive impact upon alcohol use and mental health with an interaction with degree of combat exposure. This study suggests that TLD is a useful post-deployment transitional activity that may help to improve PTSD symptoms and alcohol use in UKAF personnel.

### Systematic review and meta-analyses of psychosocial interventions for veterans of the military 10:15–10:30

#### N. Kitchiner^1^, N. Roberts^1^, D. Wilcox^2^ and J. Bisson^1^

^1^Cardiff University, Cardiff, UK; ^2^South West Veterans Mental Health Service, Bristol, UK

*Background*: The efficacy of psychosocial therapies for common mental health disorders in veterans is unclear and requires further examination. *Method*: Systematic review and meta-analyses of randomized controlled trials (RCTs). Twenty databases were searched. Studies were included if they reported a psychosocial intervention designed to treat or reduce common mental health symptoms in veterans identified as being symptomatic at the time they entered the study. Studies of substance dependency disorders and psychosis were excluded. Eligible studies were assessed against methodological quality criteria and data were extracted and analyzed. *Results*: Twenty-nine RCTs were identified. There was evidence for the use of trauma-focused therapies for posttraumatic stress disorder (PTSD) and some evidence for psychological interventions in the treatment of borderline personality disorder, depression, insomnia, and panic disorder co-morbid to PTSD. However, methodological quality of many of the studies was less than optimal. *Conclusions*: Trauma-focused psychological therapies are likely to be effective for combat-related PTSD but there is a need for more research to determine the efficacy of psychological treatments for other mental health disorders in veterans.

### Clinical focus on MUPS; assessment and treatment 10:30–10:45

#### M. Zeijlemaker^1^ and E. Vermetten^2^

^1^Royal Dutch Airforce, Utrecht, The Netherlands; ^2^Military Mental Healthcare, Utrecht, The Netherlands

*Background*: Medical unexplained physical symptoms (MUPS) have been around for ages. This so-called “diagnosis” can be considered a nightmare for the “die-hard” scientific oriented clinician. It can also be an enormous challenge. MUPS are the third most common factor in the OIF/OEF returning veterans, after musculoskeletal disorders and mental disorder and account for approx. 34% of possible diagnoses in returning veterans. The clinical phenotype consists of persistent idiopathic symptoms that drive patients to extensively seeking medical care. The symptoms are diffuse and vary from fatigue to dizziness and chronic pain. Despite hypotheses about stress-relatedness, a pathophysiological substrate, thus far, is lacking. We propose a clinical treatment program that bypasses the diagnostic pitfalls. *Method*: Patients are assessed individually by family physician and carefully screened. Upon inclusion, they are invited in multidisciplinary day treatment for 12 weekly sessions, in a closed group. Modules that are offered are cognitive behavioral therapy, graded physical therapy (fitness, psychomotor therapy focus, psycho education, and case management. We use a model of allostatic load awareness. *Results*: We present data on 104 referrals and six groups that were treated at the Dutch Military Hospital. Patients were compliant, and satisfied with the interventions. There was less medical consumption and increased job participation at the end as well as with long-term follow-up. *Conclusion*: Military service is a challenging and demanding job. Loyalty to the organization can drive individuals to performances beyond normal reason. Deployments can pose additional stressors that can lead to syndrome complexes that are poorly understood, but effectively treated.

### Military multimodal memory desensitisation and reprocessing (3MDR); first results of a new exposure-based treatment for PTSD 10:45–11:00

#### E. Vermetten^1^, L. Meijer^1^, P. Van Der Wurff^2^ and A. Mert^2^

^1^Military Mental Health—Research/UMC Utrecht, The Netherlands; ^2^Military Rehabilitation Center, Doorn, The Netherlands

*Introduction*: The dual task processing in eye movement desensitization and reprocessing (EMDR) has proven effective for the treatment of posttraumatic stress disorder (PTSD). The procedure is typically performed in sedentary condition with imaginary exposure conditions. Therapeutic adherence has reported a problem in military populations compromising treatment efficacy. *Method*: We implemented a “high end” treatment procedure that preserved dual task processing principle, yet introduced new mode of exposure. We designed a treatment based on the six DoF motion base of the computer assisted rehabilitation environment (CAREN) facility. This adds to regular therapy physical (walking) elements, virtuality with visual (pictures/words with 180 deg field of vision) and auditory (Dolby surround). We used personal pictures of soldiers’ own deployment to which they were exposed while walking on a treadmill. Pictures had been rated before on a Likert scale of affect and arousal. We used multimodular strategies to enhance affective arousal. Dual task processing was installed by an oscillating ball (moving target) superimposed on the display that was followed with the eyes. Cognitive associations were recorded and typed on the screen. One session lasted 45 minutes. We used this procedure for four weekly sessions. Aspects of presence were adhered to, to maximize possible positive outcome. *Results*: First subjects reported to be captivated by the exposure, and felt a presence by being time distorted. They performed well in the procedure. They walked a repetitive cycle while walking and viewing high affect pictures of deployment scenes. *Conclusions*: The first patient encounter with the system was positive. This has potential for avoidant patients, therapy resistant patients, and patients in who a boost is needed to enhance compliance downstream therapy. The system allows avoidant soldiers to use this mode of exposure as a useful treatment addition. Future research is to develop this in a ‘low end’ device or tool and conduct research to assess its effectivity in PTSD.

## Miscellaneous
*Symposium: New studies in the military: Prevalences and trajectories of resilience and pathology*

### Trajectories of PTSD and delayed onset in a group of 746 Danish soldiers deployed to Afghanistan—a study of long-term consequences of deployment three years after homecoming 11:45–12:00

#### M. Bertelsen^1^, D. Berntsen^2^, T. Madsen^1^ and S. B. Andersen^1^

^1^Danish Veterancentre, Danish Defence, Ringsted, Denmark; ^2^Department of Behavioral Sciences, Aarhus University, Aarhus, Denmark

*Objective*: Effective planning and tailoring of treatment for PTSD and other trauma-related disorders in military settings depend on large-scale prospective studies on long-term incidence and development. A number of studies have shown incidence rates of PTSD following military deployment on 2–12%, and it has been shown that delayed onset of PTSD in some military cohorts is high. Prospective research designs with multiple assessments over a longer period of time are scarce, due to difficulties designing such studies in a population of civilians. This prospective study investigates the development of PTSD symptoms and other trauma-related disorders and outlines different trajectories in the development of PTSD. Further, it investigates to what degree delayed onset of PTSD can be expected up to three years after deployment to Afghanistan. *Method*: A number of 746 Danish soldiers deployed for Afghanistan, Helmand Province from February 2009 to August 2009 were included in a prospective study. The study was carried out in six waves, and the soldiers were assessed before, during, and at homecoming, three months after, seven months after and three years after homecoming. Primary outcome measures are PTSD, depression, anxiety, and substance abuse. A sub sample of the cohort was analyzed using latent class growth analysis to identify different trajectories of the development of PTSD. *Results*: It was shown that 9.6% of the soldiers displayed PTSD-symptoms above a cut-off at 44 on the PCL-C scale three years after homecoming. A total of 4% were delayed onsets. Further, our analysis revealed six different trajectories of PTSD, one large resilient group and five groups that showed variable patterns of vulnerability of early or late onset of PTSD symptoms.

### Persistence of stress sensitization following deployment in soldiers with high early life trauma 12:00–12:15

#### G. Smid^1^, M. Van Zuiden^2^ and E. Vermetten^3^

^1^Foundation Centrum '45 / Arq Research Program, Diemen, The Netherlands; ^2^Academic Medical Centre, Amsterdam, The Netherlands; ^3^Research Centre—Military Mental Health, Utrecht, The Netherlands

*Background*: Stress sensitization, i.e., increased responsiveness to stressful life events has been found in high trauma exposed adults within the first 18 months following trauma exposure (Smid et al., 2012) as well as in young children (Grasso, Ford, & Briggs-Gowan, 2012). However, it is unclear whether stress sensitization may persist over time. We hypothesized that soldiers exposed to high levels of early life trauma would be at risk of persistence of stress sensitization 2 years following deployment. *Method*: In a cohort of Dutch soldiers deployed to Afghanistan (N=814), we investigated the effects of stressful life events reported 1 and 2 years after deployment on change in PTSD symptoms. Early life trauma was assessed prior to deployment. Data were analyzed using latent growth modeling. Using multiple group analysis, we examined whether high early life trauma moderated the relation between post-deployment stressors and linear change in posttraumatic distress after deployment. *Results*: Stressful life events reported during the first year were associated with a steeper linear increase in PTSD symptoms post-deployment (from 2 to 26 months) in high combat stress exposed soldiers. Stressful life events reported during the second year continued to predict a steeper linear increase in high combat stress exposed soldiers with high early life trauma, but not in other soldiers. *Conclusion*: Stress sensitization following high combat stress exposure may persist in soldiers with high early life trauma during two years following return from deployment.

References

Grasso, D. J., Ford, J. D., & Briggs-Gowan, M. J. (2012). Early life trauma exposure and stress sensitivity in young children. *Journal of Pediatric Psychology, 38*(1), 94–103. doi: 10.1093/jpepsy/jss101

Smid, G. E., Van der Velden, P. G., Lensvelt-Mulders, G. J. L. M., Knipscheer, J. W., Gersons, B. P. R., & Kleber, R. J. (2012). Stress sensitization following a disaster: A prospective study. *Psychological Medicine, 42*(8), 1675–1686. doi: 10.1017/S0033291711002765

### Predicting PTSD: identification of pre-deployment biological vulnerability factors 12:15–12:30

#### M. Van Zuiden^1^, E. Geuze^2^, E. Vermetten^2^, A. Kavelaars^3^ and C. Heijnen^3^

^1^Department of Psychiatry, Academic Medical Center, University of Amsterdam, The Netherlands; ^2^Research Centre for Military Mental Health, Utrecht, The Netherlands; ^3^Laboratory for Neuroimmunology and Developmental Origins of Disease, University Medical Center, Utrecht, The Netherlands

The presence of PTSD is associated with numerous biological changes. However, it remains unknown whether these previously observed biological correlates of PTSD precede trauma-exposure and development of PTSD, and thus, represent a biological vulnerability factor for the development of PTSD. Alterations in the functioning of the glucocorticoid receptor (GR), which mediates the effects of cortisol throughout the body, is one of the previously observed biological correlates of PTSD. We hypothesized that altered functioning of the GR pathway is already present before PTSD development and represents a vulnerability factor. Therefore, we investigated the predictive value of several components of the GR signalling pathway for the development of high levels of PTSD symptoms in response to military deployment to a combat-zone. We included approximately 1,000 Dutch soldiers prior to their deployment to Afghanistan. Of these participants, 76% completed the assessment 6 months after return from deployment. A variety of GR pathway components were assessed in peripheral blood collected prior to deployment (e.g., GR receptor number, GR target gene messenger RNA expression). PTSD symptoms were assessed prior to deployment and 6 months after return. We will summarize the results of our study, which show that several components of the GR pathway measured prior to deployment to a combat-zone independently predicted development of high levels of PTSD symptoms, as assessed 6 months after return from deployment. In all, these results show that the GR pathway in peripheral blood is a vulnerability factor for development of a high level of PTSD symptoms. Implications of the identification of biological vulnerability factors will be discussed.

### Prevalence of delayed-onset PTSD in military personnel: is there evidence for this disorder? Results of a prospective UK cohort study 12:30–12:45

#### L. Goodwin^1^, M. Jones^1^, R. Rona^1^, J. Sundin^2^, S. Wessely^1^ and N. Fear
^1^King's Centre for Military Health Research, King's College London, London, UK; ^2^Academic Centre for Defence Mental Health, King's College London, London, UK

*Objective*: Delayed-onset posttraumatic stress disorder (PTSD) is defined as onset at least 6 months after a traumatic event. This study investigates the prevalence of delayed-onset PTSD in a representative UK military sample. *Method*: One-thousand three-hundred ninety-seven participants from a two-phase prospective cohort study of UK military personnel. Participants had been deployed to Iraq before the phase 1 assessment and were not deployed again during the study period. Delayed-onset PTSD was categorized as participants who did not meet the criteria for probable PTSD (assessed by the PCL-C) at phase 1, but who met the criteria by phase 2. *Results*: 3.5% of participants met the criteria for delayed-onset PTSD, representing 46% of all PTSD cases assessed at both phases 1 and 2. Twelve (27%) had previously met the criteria for sub threshold PTSD at phase 1. Sub threshold PTSD, common mental disorder (CMD), poor/fair self-reported health, and multiple physical symptoms at phase 1, and the onset of alcohol misuse or CMD between phases 1 and 2, were associated with delayed-onset PTSD. *Conclusions*: Delayed-onset PTSD exists in this UK military sample. Military personnel who developed delayed-onset PTSD were more likely to have psychological ill health at an earlier assessment, and clinicians should be aware of potential co-morbidity in these individuals, including alcohol misuse. Leaving the military, or experiencing relationship breakdown, was not associated.

## Afternoon Miscellaneous
*Symposium: Clinical pathways regarding trauma responses among journalists*

### Ethical dilemmas as a predictor for PTSD in news journalists working with large-scale violence 15:15–15:35

#### K. Backholm^1^ and T. Idaas^2^

^1^Abo Akademi University, Turku, Finland; ^2^The Norwegian Union of Journalists, Oslo, Norway

During work in crisis situations, a news journalist may be exposed to potentially traumatic events via personal exposure or in a more indirect manner when meeting first-hand victims. Thereby, journalists are at risk for developing long-term post-crisis stress reactions. Identifying the occupation-specific risk factors is important for promoting mental health among news professionals. In this study, possible relationships between experienced ethical dilemmas during a crisis assignment and level of PTSD symptoms were investigated with a web-based survey. The sample consisted of 371 Norwegian news journalists who worked during the Oslo/Utöya terror attack in 2011. The concept of journalistic ethical dilemmas as a risk factor is inspired by the work on moral injury in soldiers by Litz and colleagues (2009). Dilemmas were, in the current study, defined as doubts occurring when the journalist experienced going beyond individual work-related ethical boundaries when for example, carrying out dubious orders received from the home office. The study follows up findings from studies with Finnish journalists working in school shootings (Backholm, 2012) and Norwegian journalists covering the 2004 tsunami (Idås, 2010). Preliminary results show that ethical dilemmas of several subcategories predicted a higher level of PTSD symptoms. Going beyond ethical boundaries seems to be an important factor for understanding possible pathways to impairment in journalists, but further refinement of the concept and more research is needed.

References

Backholm, K. (2012). *Work-related Crisis Exposure, Psychological Trauma and PTSD in News Journalists*. PhD Thesis, Åbo Akademi University, Developmental Psychology.

Idås, T. (2010). Journalistene og tsunamien: Ekstreme inntrykk – men dilemmaene stresset mest. Master Thesis, University of Oslo.

Litz, B. T., Stein, N., Delaney, E., Lebowitz, L., Nash, W. P., Silva, C., et al. (2009). Moral injury and moral repair in war veterans: A preliminary model and intervention strategy. *Clinical Psychology Review, 29*, 695–706. doi: 10.1016/j.cpr.2009.07.003

### The effects of occupational-related intimidation among journalists: a predictor of posttraumatic stress symptoms 15:35–15:55

#### S. Drevo^1^, K. Parker^1^, E. Newman^2^, B. Brummel^1^ and N. Cook^1^

^1^University of Tulsa, Tulsa, USA; ^2^University of Tulsa and Dart Center for Journalism and Trauma, Tulsa, OK, USA

Although studies have demonstrated high rates of posttraumatic stress symptoms among victims of workplace harassment (Matthiesen & Einarsen, 2004; Tehrani, 2004), work-related intimidation and harassment among journalists has not been examined. The current study examined English speaking journalists who responded to an online survey assessing journalists’ workplace experiences. This study was conducted to determine whether occupational-related intimidation would predict posttraumatic stress symptoms when controlling for previous occupational-related trauma exposure. Initial regression analyses (N=155) revealed a significant prediction model with intimidation as a statistically significant predictor of posttraumatic stress symptoms, irrespective of the contributions of previous occupational-related trauma exposure. The results from our sample suggest that occupational-related intimidation tactics (e.g., followed on foot, phone tapped, damaged property) have a more deleterious effect on psychological well-being than does previous exposure to work-related traumatic events (e.g., mass casualties, war zones, natural disasters, etc.), particularly regarding the experience of posttraumatic stress symptoms. Posttraumatic stress symptoms among journalists may not only be a function of, and a manifestation from, the very nature of the stories they pursue, but from experiencing intimidation due to their occupation. Results will be updated based on sample size at the time of the conference.

Reference

Matthiesen, S. B., & Einarsen, S. (2004). Psychiatric distress and symptoms of PTSD among victims of bullying at work. *British Journal of Guidance and Counselling, 32*, 335–356.

Tehrani, N. (2004). Bullying: A source of chronic posttraumatic stress? *British Journal of Guidance and Counselling*, *32*, 357–366.

### Predictors for posttraumatic growth among journalists covering large-scale violence 15:55–16:15

#### T. Idaas The Norwegian Union of Journalists, Oslo, Norway

A survey investigating the potential stressful experiences faced by first responders and journalists working in the disaster area after the 2004 Tsunami indicated that the journalists, compared to the first responders, were far more exposed to grotesque impressions of the disaster scene. They also experienced significantly higher levels of stress reactions than first responders. At the same time 9 out of 10 journalists answered that they were glad to take part in the coverage and that they would be happy to take part in the coverage of a similar situation in the future. (Idås, 2010; Thoresen, 2007). This paradox was investigated further in the current study, focusing on 371 Norwegian news journalists covering the terror attack in 2011, and the aftermath (response rate 72%). The web-based survey included questions covering social support, PTSD (IES-R) and, posttraumatic growth. Preliminary results from the July 22-study show that posttraumatic stress was significantly related to posttraumatic growth. It also indicates that the level of posttraumatic growth was related to the type of stressor the journalists were exposed to and the social support and recognition they experienced.

References

Idås, T. (2010). Journalistene og tsunamien: Ekstreme inntrykk—men dilemmaene stresset mest, Master Thesis, University of Oslo.

Thoresen, S. (2007). Mestring og stress hos innsatspersonell og journalister mobilisert til Tsunamikatastrofen, Nasjonalt kunnskapssenter om vold og traumatisk stress (NKVTS), Rapport nr 2/2007.

## Impact of trauma on communities
*Symposium: Creating resilience in organisations*

### Managing the risk of trauma in high risk policing roles 16:45–17:05

#### R. Hooke Metropolitan Police, London, UK

London's Metropolitan Police Child Abuse Investigation Command employs over 600 officers and staff and is part of the Specialist Crime and Operations Directorate. In addition to 16 London borough Child Abuse Investigation Teams there are some 60 officers and staff involved in investigating the activities of organized on-line child sex abusers and others involved in the manufacture and distribution of child abuse images. This work is supported by a computer forensic team providing intelligence on offender activities, covert on-line investigations and officers involved in interviewing offenders and their child victims. In March 2011, a review was conducted by the presenter, the then Head of the Paedophile Unit, into the risks that this work presented to staff and the mechanism required to manage that risk. Although there had been a program of support provided by Occupational Health, it was now felt that a more scientific approach was needed which provided anonomized management information to be used to reduce the trauma risks. Previous research had indicated that this work presented a risk and that dealing with child sex abuse involved many variables which could only be managed using a holistic approach and skilled professionals. With the support of a specialist psychologist and criminologist, a comprehensive review was undertaken to identify who needed support and what kind of support would be most effective. The adopted program was reviewed by ACPO and recognized as best practice. Over the past two years, each officer within the Directorate has gone through a comprehensive psychological screening involving the use of psychometric questionnaires and a structured interview. This initial screening has been followed up with regular follow up screening. This presentation will provide results from the screening and evidence of the factors which lead to increased resilience and the organizational and performance benefits that have resulted.

### Training and support for front-line officers and staff in dealing with trauma 17:05–17:25

#### E. Eades Surrey Police, Guildford, UK

Surrey Police is responsible for policing the county of Surrey in South East England. The Occupational Health Department is based at Force Headquarters in Guildford. Surrey Police have been proactive in finding ways to support their 1,840 regular police officers, 278 special constables, 211 police community support officers, and 2,000 support staff. Despite having one of the lowest crime rates in the UK, Surrey has had to deal with a number of high profile murders and has one of the busiest motorway networks in the country. The force has been proactive in its approach to supporting its officers and staff and has initiated a risk assessment process, which has identified a number of roles as being high risk. However, it is mindful that policing can place significant pressure and stress on its workers and therefore has been running a program of training for front line managers and supervisors, which train them in how to deal with a crisis and how to provide support to members of their teams. This presentation will provide a brief outline of the training and support provided to teams when handling traumatic operations including shooting, murder, major road crashes, child abuse, and many other challenging policing events. The crisis management, demobilizing, and defusing interventions undertaken by front line supervisors will be described together with the additional support, which is made available from the Occupational Health service. The last part of the presentation will review the evaluation processes and results and describe how the program is reviewed and adapted to the needs of the officers and staff.

### The role of RoadPeace in building resilience in bereaved family members 17:25–17:45

#### N. Tehrani Twickenham, London, UK

RoadPeace is a charity created for all victims of road crashes and was set up in response to the desperate need of road crash victims for timely information, immediate, and long-term support and practical help with legal proceedings. RoadPeace provides a range of support services for road crash victims including emotional support, practical information, and an advocacy and casework service together with a tenacious campaigning presence, championing the rights of road crash victims and working to improve road safety in the UK and wider through its representations in Europe and beyond. The focus of this presentation is the support provided to groups of bereaved, affected by deaths on the roads. Unfortunately, despite the large number of people affected by road deaths, (around 13,000 each year in the UK), many find that there is little support to meet their particular needs. The goal of RoadPeace was to introduce a brief, economically viable and effective group model of resilience building to support bereaved families and friends. The six seminars followed a manual-based approach, and each participant had a workbook which was used during the sessions and for their homework. Each session began with an outline of the session's goals, a review of the previous homework, an introduction to new information, psycho-education and skills training, practice and homework setting. The findings from the program are encouraging and suggest that the intervention has been successful. Whilst not designed as a treatment for posttraumatic stress, clinically significant improvements have been found in anxiety, depression, and trauma scores plus some improvement in lifestyle. More important has been the impacts of the program in helping the participants re-engage with others and to learn skills for dealing with distressing symptoms. The qualitative results provide a strong endorsement for the program.

## ORAL, JUNE 8 HALL STUART TUDOR

## Morning Cultural issues and trauma
*Symposium: Mental health of children and adults exposed to mass violence and conflict*

### Relationship of posttraumatic stress and persistent pain and somatization 10:00–10:15

#### N. Morina^1^, U. Schnyder^1^, C. Martin-Soelch^2^ and J. Mueller^1^

^1^University Hospital Zurich, Zurich, Switzerland; ^2^Department of Psychology, University of Fribourg, Fribourg, Switzerland

This cross-sectional study examined the prevalence and correlates of persistent pain and somatization in the aftermath of war, and the role of traumatic exposure and posttraumatic stress symptoms in their development. Civilian war survivors (*n*=147, 55.8% female; mean age: 43.8) from Kosovo were assessed regarding their trauma history, posttraumatic stress symptoms, current persistent pain, and somatization. Participants reported on average more than five types of traumatic war exposure, 41 (27.9%) met criteria for a PTSD, which was associated with higher persistent pain and somatization symptoms. 54.8% reported somatization symptoms and an average of 3.2 (1–6) persistent pain. Hyper-arousal symptoms mainly explained the relationship of posttraumatic stress symptoms and persistent pain and somatization. The preliminary results indicate that persistent pain and somatization are highly prevalent among war-exposed civilians and that hyper-arousal may be a significant factor in understanding and treating traumatized people who are experiencing pain and somatization.

### Trauma, mental health and intergenerational aspects: Kosovar children and their parents 11 years after the war 10:15–10:30

#### M. Schick, N. Morina, R. Klaghofer, U. Schnyder and J. Mueller University Hospital Zurich, Zurich, Switzerland

*Objective*: This cross-sectional study investigated the intergenerational interplay of trauma-related mental health problems among families 11 years after the Kosovo war. *Methods*: In a cross-sectional study, 51 randomly selected Kosovar families (encompassing school-age child, mother and father) were examined with regard to traumatic exposure, posttraumatic stress (UCLA, PDS), anxiety, (SCAS, HSCL-25) and depressive symptoms (DIKJ, HSCL-25) as well as differential intergenerational aspects. *Results*: Considerable trauma load and high prevalence rates of posttraumatic stress, anxiety, and depression were found in both children and parents with mothers showing the highest symptom scores. While strong correlations were found between children's depressive symptoms and paternal posttraumatic stress, anxiety, and depression, maternal symptomatology did not correlate with child mental health. In multiple regression analyses, children's depressive symptoms were significantly related only to the PTSD symptom severity of their fathers. *Conclusion*: Eleven years after the Kosovo war the presence of posttraumatic stress, anxiety, and depressive symptoms in civilian adults as well as children is still substantial. In this sample if war survivors, children's depression seems to be related to paternal, but not maternal, psychological impairment. This finding may be reflective of the centric position of fathers within the Kosovar familial system and should be considered in the treatment of families affected by trauma.

### Effects of trauma on families and children: migration and aggressive behavior in children of traumatized parents 10:30–10:45

#### J. Mueller and N. Morina University Hospital Zurich, Zurich, Switzerland

*Background*: Research shows correlations between posttraumatic stress disorder (PTSD) and aggressive behavior. It is unclear, however, if forced migration into exile adds to these problems. The aim of our study was to compare aggressive behavior of children whose parents had been traumatized by the Kosovo war who live in exile (Switzerland) with the same behavior of children still living in their home country. *Methods*: We assessed N=150 pairs of children and at least one of their parents, N=114 of those were still living in Kosovo. Trained interviewers conducted the assessment that included traumatic event types, posttraumatic stress disorder (UCLA PTSD INDEX for DSM-IV and Posttraumatic Diagnostic Scale), aggression, (The Aggression Questionnaire) and the children's social behavior (Strengths and Difficulties Questionnaire children version). *Results*: Children of the Swiss sample indicated significantly more traumatic event types as those of the Kosovar sample. However, children of the Kosovar sample showed higher PTSD symptom severity as well as higher aggression than the Swiss sample. Children of both samples did not differ regarding their social behavior. Generally, PTSD symptom severity was correlated with aggression and social behavior respectively. *Discussion*: The rates of posttraumatic stress disorder in Kosovar adults and their children are still high 11 years after the Kosovo war. According to previous studies, aggression was correlated with PTSD symptom severity. Possibly, the fact of living in a post-conflict country is more stressful than having to adapt to a new culture of a safe exile, leading to a higher vulnerability of individuals living under the first condition.

### Posttraumatic stress and prolonged grief disorder in resettled Iraqi refugees 10:45–11:00

#### A. Nickerson and R. Bryant University of New South Wales, Sydney, Australia

While reserach has documented high levels of psychopathology in refugees, there is inadequate research identifying symptom profiles in individuals exposed to mass trauma and violence. This study employed latent class anlaysis to determine whether there are distinctive classes of bereaved people based on PTSD and PGD symptom profiles following mass trauma. Participants were 248 Mandaean adult refugees who were assessed at an average 4.3 years since entering Australia following persecution in Iraq. Latent class analysis revealed four classes of participants: 1) PTSD/PGD class (16%), 2) predominantly PTSD class (25%), 3) predominantly PGD class (16%), and 4) low symptoms class (43%). Predictors of class membership were also investigated. These findings provide evidence of specific symptom patterns following exposure to mass trauma and loss, which are associated with different types of pre- and post-migration experiences.

## Evidence-based practice on trauma
*Symposium: Evidence based community approaches for survivors of childhood trauma in Germany*

### Developmentally Adapted Cognitive Processing Therapy for adolescents and young adults with PTSD symptoms after physical and sexual abuse: design of a randomized clinical trial 11:45–12:05

#### A. Vogel^1^, H. Koenig^2^, F. Neuner^3^, B. Renneberg^4^, U. Schmidt^5^, R. Steil^6^ and R. Rosner^1^

^1^Department of Clinical and Biological Psychology, Catholic University Eichstaett-Ingolstadt, Eichstaett, Germany; ^2^Department of Medical Sociology and Health Economics, University Medical Center Hamburg-Eppendorf, Hamburg, Germany; ^3^Department of Psychology, University of Bielefeld, Bielefeld, Germany; ^4^Department of Education and Psychology, Freie Universität Berlin, Berlin, Germany; ^5^Max Planck Institute of Psychiatry, Munich, Germany; ^6^Department of Clinical Psychology and Psychotherapy, Johann Wolfgang Goethe University, Frankfurt, Germany

Although the severe consequences of childhood sexual or physical abuse (SA/PA) for mental and physical health are well known and may dramatically reduce quality of life, evidence for psychotherapeutic interventions for adolescents and young adults is lacking. Many survivors develop chronic posttraumatic stress disorder (PTSD). The D-CPT consortium addresses the need to evaluate an evidence-based treatment for PTSD related to SA/PA in adolescents using a new cognitive behavioural psychotherapy called developmentally adapted cognitive processing therapy (D-CPT; study 1) as well as the need to assess further consequences of effective treatment of PTSD for the patients themselves as well as for society: study 2 aims to assess possible moderators of treatment response; study 3 will focus on PTSD-related epigenetic changes and whether these changes are reversible through effective intervention; study 4 investigates whether direct health care costs (i.e., treatment costs) and indirect costs of the disorder (unemployment, productivity losses) can be reduced due to successful treatment; study 5 assesses the neurophysiological processing of threat cues in PTSD and their respective changes in the course of treatment. Study 1 investigates the efficacy of the already piloted D-CPT outpatient treatment manual in an open, rater-blinded, multicenter, randomized clinical trial. Developmental adaptations of the therapy protocol concern emotion processing, therapy format, and addressing typical developmental tasks. The manual will be explained in greater detail. The intervention has been piloted successfully and a very large effect size has been obtained. For the RCT the consortium will recruit 90 participants aged 14–21 years who suffer from PTSD after SA/PA across three study sites in Germany. The trial will include assessments at pre-treatment, post-treatment, and three and six month follow-up comparing D-CPT with treatment as usual (TAU).

### Childhood abuse and neglect as a cause and consequence of substance abuse—understanding risks and improving services (CANSAS) 12:00–12:25

#### I. Schäfer^1^, S. Barnow^2^, M. Klein^3^, M. Muhlhan^4^, N. Scherbaum^5^, M. Driessen^6^, R. Thomasius^7^, U. Ravens-Sieberer^8^, M. Haerter^9^ and S. Pawils^9^

^1^Department of Psychiatry and Psychotherapy, Universit Medical Center Hamburg-Eppendorf, Germany; ^2^Department of Clinical Psychology and Psychotherapy, University of Heidelberg, Germany; ^3^Catholic University of Applied Sciences, Cologne, Germany; ^4^Institute of Clinical Psychology and Psychotherapy, Technische Universität Dresden, Germany; ^5^Department of Psychiatry and Psychotherapy, University of Duisburg-Essen, Germany; ^6^Department of Psychiatry and Psychotherapy, Bethel, Bielefeld, Germany; ^7^German Center for Addiction Research in Childhood and Adolescence (DZSKJ), Hamburg, Germany; ^8^Department of Child Public Health and Health Psychology, University Medical Center Hamburg-Eppendorf, Germany; ^9^Institute of Medical Psychology, University Medical Center Hamburg-Eppendorf, Germany

Substance use disorders (SUD) belong to the most frequent behavioral consequences of childhood abuse and neglect (CAN). Lifetime diagnoses of SUD are found in about 20% of adult survivors of CAN in community samples and in 30% of individuals who seek treatment for the consequences of CAN. Conversely, 24–67% of all patients in treatment for SUD have a history of CAN, which makes them one of the groups with the highest burden of CAN in the health care system. Moreover, parental substance abuse and dependence is one of the most important risk factors for the perpetration of CAN. Regarding both perspectives, CAN as cause and as a consequence of SUD, a better understanding of relevant mediators and risk factors is necessary, to improve prevention and develop adequate treatments. Given the high prevalence of SUD in survivors of CAN and its important role as a risk factor for the perpetration of CAN, the objectives of the CANSAS-network are 1) to gain a better understanding of the relationships between these two important public health problems, 2) to provide evidence-based treatments for survivors of CAN with SUD, and 3) to provide services with trainings to improve the assessment of CAN among clients with SUD assess risk factors for the perpetration of CAN in this population. These aims will be achieved in a multidisciplinary network, including experts in the fields of trauma treatment for patients with (comorbid) SUD, epidemiology and risk factor research, biological, and psychological moderators, as well as health services research.

### “Recognize-realize-act” strategic concept to improve child protection in children's hospitals in Saxony 12:25–12:45

#### J. Schellong^1^, A. Neumann^1^, A. Heilmann^2^, F. Schwier^3^, U. Schmidt^4^, C. Erfurt^4^, R. Berner^2^, G. Fitze^3^ and K. Weidner^1^

^1^Department of Psychotherapy and Psychosomatic, Medical Faculty Technical University of Dresden, Dresden, Germany; ^2^Department of Child and Youth Medicine, Medical Faculty Technical University of Dresden, Dresden, Germany; ^3^Department of Child Surgery, Medical Faculty Technical University of Dresden, Dresden, Germany; ^4^Institute of Forensic Medicine, Technical University Dresden, Dresden, Germany

*Background*: A major risk factor for impaired health in later life is experiencing violence in childhood. Throughout childhood almost every person will be seen by representatives of the health care system. Therefore, these representatives hold a key position in recognizing signs and symptoms of violence or child abuse. In most cases though, caregivers are unaware of their important role. Moreover, a previous survey of awareness of caregivers of this topic has shown high levels of uncertainty how to act in cases of suspected child maltreatment. Special training programs, adapted to the realities of medicine, are needed to improve the appropriate care of victims. *Methods*: Previously recorded material on how to act in case of suspected child maltreatment was adapted and approved in a pilot project in hospitals in the City of Dresden in 2011.These experiences helped to generate a strategic concept to introduce and strengthen child protection groups in children's hospitals in Saxony. The aim is to establish a standard for all clinics including already existing material and procedures as well as networking processes with the youth welfare system. The project is explicitly supported by the government of Saxony and covers the period of 03/2012–12/2013. *Results*: The project is currently in the implementation and training phase with a high rate of participating hospitals (40 out of 43). Adaption of the existing documents and the privacy-oriented communication to the local youth welfare system in case of suspected child maltreatment proves worthwhile. Regional conferences with all professions involved already took part in the first hospitals. *Conclusion*: The project receives highly positive responses from a huge number of targeted clinics. One of the reasons for acceptance may be the government-based approach. This corroborates the importance of political support in addition to standardized procedures dealing with child protection issues.

## Afternoon
*Open Papers: Children and young people I*

### “You, son of an Interahamwe!”—The trauma of being a child of a genocide suspect in Rwanda 16:00–16:15

#### H. Rieder and T. Elbert University of Konstanz, Konstanz, Germany

Psychological research in post-conflict societies often focuses on mental health problems or social changes regarding different groups of a population. Children and adolescents whose parents were accused of participation in acts of violence are mainly missing in these studies. The present study aimed at deepening the understanding of how descendants’ deal with their parents’ past and how they integrate affectively what they know about what had happened. In 2010 and 2011, a qualitative study was conducted applying in-depth interviews with descendants of former prisoners and genocide suspects in Muhanga, in the Southern Province of Rwanda. Ten young adults (age range 19–31 years old) who survived genocide in 1994 and who have had at least one parent in prison over the past years were interviewed using a narrative biographical approach. Additionally, questions were included on their own traumatic experiences, communication on the parent's participation, truth-seeking strategies, and parent–child relationship during and after imprisonment. Data were then combined with findings from a quantitative analysis on communication, relationship patterns, and changes in the course of life of the descendants based on 60 structured interviews conducted within the same setting. A first explanatory model was produced to describe internal processes of dealing with the parent‘s past over the interviewees’ lifetime. It suggests that rather than openly criticizing their parents, descendants point at their own inhibited development of autonomy, parentification processes, and forced premature adulthood, and experienced discrimination as stressful consequences of their parents’ actions. The parent's confession is a determining factor in how emotions can be expressed and how they can be further processed by descendants. These preliminary findings are discussed with regard to the current Rwandan context.

### Types of attachment among adults victimized as children 16:15–16:30

#### A. Widera Wysoczanska Institute of Psychology, University of Wroclaw, Wroclaw, Poland

The aim of the study was to discover links between childhood victimization on one side and the type of parent–child relationship and the attachment in adulthood on the other side. The sample consisted of 50 females and males who were victimized in their childhood (sexually, physically, and by substance abuse), 25–55 years of age, selected by purposeful sampling. “The Type of Abuse Questionnaire” and “Intimate Situations Questionnaire” were used to determine the type of childhood abuse experienced by the investigation group. The questionnaire “Retrospective Perception of Parental Attitudes” was used to identify the sample group's retrospective and subjective perception of their parents’ parental attitudes. Four dimensions of parental behaviors were measured: support and distance, interpersonal boarders, meeting the needs of a child, and acceptance of own parenting. The types of attachment in adulthood were investigated using the “Attachment Styles Questionnaire” by Plopa. In the sample group, sexual violence experienced in childhood significantly correlated with a distance parent, transition of interpersonal borders, and lack of acceptance of oneself as a parent—these correlated with disorganized and ambivalent attachment in adulthood. Physical abuse experienced in childhood significantly correlates with lack of support, restricted meeting of a child's needs, and disorganized and ambivalent attachment. Parents’ addiction significantly correlates with lack of support and restricted meeting of a child's needs as well as with escaped attachment in adulthood. The stages of an integrated therapeutic program for persons who experienced childhood abuse are discussed.

## Open Papers: Children and young people II

### Traumatic experiences and psychological well-being of unaccompanied refugee minors in Germany 16:45–17:00

#### V. Mueller, J. Morath, J. Baumann, M. Schauer, K. Dohrmann, D. Isele, T. Elbert and M. Ruf-Leuschner Department of Psychology, University of Konstanz, Konstanz, Germany

*Objective*: Only little is known about the mental health of unaccompanied refugee minors (URM) who seek asylum in Europe. The objective of this cross-sectional study is to investigate the extend of mental health problems (PTSD, Depression, Aggression) of URM and to assess the exposure to adversity, with a focus on family and organized violence. *Methods*: A total of 56 unaccompanied refugees who came to Germany as minors were interviewed by trained clinical psychologists. The participants were between 13 and 21 years of age. They came from 20 different countries of origin. Adverse and traumatic life experiences including domestic and organized violence were assessed using structured interviews. Moreover, mental health problems including symptoms of PTSD, depression, and somatization, and also behavioral problems and appetitive aggression were investigated. *Results*: More than two-thirds of the respondents had experienced organized violence and 77% had lost at least one parent. A minimum of two traumatic events were reported. The greater the exposure to organized violence the greater was the severity of trauma-spectrum symptoms. 36% of the URM fulfilled the DSM-IV criteria for PTSD. Half of the participants presented with moderate to severe depression symptoms. Violence experienced within the family correlated with the amount of self-reported violent acts. *Conclusion*: The preliminary results of this study indicate that unaccompanied refugees, who immigrated to Germany under the age of 18, frequently suffer from mental disorders. The substantial experience of adversity and violence may contribute to limited self-control and thus reduce the threshold for aggressive behavior. It is important to sensitize social workers and legal guardians for the problems of this group and to improve their access to mental health services.

### To be a therapist and a parent in shared trauma situations 17:00–17:15

#### O. Nuttman-Shwartz^1^, R. Dekel^2^ and T. Lavi^3^

^1^Sapir college, Israel; ^2^Bar Ilan University, Ramat Gan, Israel; ^3^Sderot Resilience Center, Qassam-ridden, Israel

Recent literature has been focusing greater attention on the challenges and the implications of situations, in which both the intervener and the client are exposed to a similar threat which is coined as S*hared Traumatic Reality (STR)*. The research examined the experiences of being a professional and a parent in continuing STR situations. Three focus groups with 30 therapists from different social services in the areas exposed to continuous missiles attacks were conducted. The meetings were recorded, documented, and analyzed by two scholars. The results show that despite the intensive and ongoing professional work in situations of STR, the participants felt a sense of professional competence and growth and were confident about their ability to continue helping the residents of their locality. In addition, they use a continuum of strategies for handling the demands of the personal and professional spheres in the context of STR. While the family domain found to be the vulnerable space for the participants in this study, the professional role served as a shield that enabled the participants to continue functioning. During the presentation, we will present the complexity of the STR phenomena, factors which contributed to this positive feeling as well as the necessity to strengthen the therapists’ family functions and to help the therapists to deal better with their dual role.

### The predictive role of parental and child reported social support in therapy with traumatized children 17:15–17:30

#### T. Holt^1^, T. Jensen^2^ and S. Ormhaug^1^

^1^Norwegian Centre for Violence and Traumatic Stress Studies, Oslo, Norway; ^2^Department of Psychology, University of Oslo, Oslo, Norway

*Aims*: Social support has been found to play a significant role in the development and maintenance of different types of problems after traumatic experiences. In the child field, however, there is still a need for studies investigating this. This presentation focuses on 1) the contribution of social support (as reported from both youth and parents) on the child's trauma related symptoms pretreatment, and 2) the role of social support on the child's therapeutic change. *Method*: Data is derived from an effectiveness study in Norway comparing TF-CBT with treatment as usual (TAU) in eight ordinary community clinics where 156 traumatized children aged between 10 and 18 (M age=14.7, SD=2.2, 80.5% girls) and their primary caregivers are investigated. Instruments examining youth's trauma exposure, their trauma related symptoms, their perceived social support, and parental reported support were employed. *Results*: Linear regression analyses demonstrated that *youth's own* perceived social support was significantly linked to all trauma-related symptoms pre-treatment; indicating that less self-perceived support was related to higher symptom level. Further, this association was stronger among the oldest youth. *Parental reported support* had a significant impact on outcome, but contrary to our expectations, more support was related to higher symptom-level post-therapy. Treatment group moderated this effect, as it was only present in TAU-group. Age had no moderating effect. *Conclusion*: Findings show that social support is related to child's trauma symptoms, but that youth own perceived support and parental reported support play somewhat different roles within this sample.

### The integration of traumatic experiences and the changes of relationship values of perpetrators of domestic violence (PoDV) 17:30–17:45

#### D. Dyjakon University of Lower Silesia, Wrocław, Poland

*Background*: This study is part of a wider program of research relating to relationships in violent partners. Traumatic events experienced by PoDV are critical to the violence strategy development in their adult lives. The study involved 36 men who had carried out violence against partners and children. This research sought to address: “whether the therapeutic work on the integration of traumatic events has any impact on the change in valuation of relationships especially with a partner and children?” *Methods*: 1. Collecting research material by autobiographical narrative interview (Schütze, 1983). Transcripts of interviews subject to analysis with valuations according to “Method of Self-confrontation” (Hermanas, Hermans-Jansen 1995). Narrative question: “Tell me the story of your life with particular emphasis on relationships with other people, especially loved ones.” 2. The partnerships test (Shostrom and Kavanaugh, 1975) describes the type of current relationships. 3. The nature of psychotherapy was integrative therapy of groups, consisting of offenders, with the primary aim of integrating traumatic events. The study was conducted in three stages: 1) a narrative interview and partnerships test (completed by the offenders and their partners); the psychotherapy for perpetrators; 3) an interview with offenders. *Results*: The research showed that: 1) Traumatic experiences influence the formation of values such as: anger and hatred, unfulfilled longing, and helplessness and isolation (Hermans, Hermans-Jansen, 1995), which during therapy were transformed into primarily values of “strength and unity”. 2) Based on the collective material of the research, seven types of relationships were identified and transformative patterns distinguished for each partnership type. *Conclusions*: Drawing attention to using valuations as a method of self-confrontation in PoDV psychotherapy is an important step to deepen and consolidate the changes their relationships with other people, to understand other people's experiences and establishing acceptable standards of social functioning.

### Keeping children in evidence-based trauma treatment: factors impacting attrition 17:45–18:00

#### G. Sprang^1^, C. Craig^2^, J. Clark^3^, K. Vergon^4^, J. Cohen^5^, M. Staton-Tindall^6^ and R. Gurwitch^7^

^1^Department of Psychiatry, Center on Trauma and Children, University of Kentucky, Lexington, USA; ^2^Unviersity of Kentucky, Center on Trauma and Children, Lexington, USA; ^3^University of Cincinnati, School of Social Work, Cincinnati, USA; ^4^Louis de la Parte Florida Mental Health Institute, University of South Florida, Tampa, USA; ^5^Allegheny General Hospital, Pittsburg, PA, USA; ^6^University of Kentucky, Center on Trauma and Children, Lexington, USA; ^7^Duke University, Psychiatry and Behavioral Sciences, Chapel Hill, USA

This study expands our understanding of treatment attrition by investigating factors predicting treatment drop out in a large national data set of clinic-referred children and parents seeking trauma-specific evidence-based practices. Using de-identifed data (N=2579) generated by the National Child Traumatic Stress Network (NCTSN) Core Data Set (CDS) collected between Spring 2004 and Fall 2010, the study uses sequential logistic regression analyses to assess prediction of the probability of a given subject having prematurely dropped out of treatment. The findings of this study suggest that race, placement in state custody, and a diagnosis of posttraumatic stress disorder, oppositional defiant disorder, and major depressive disorder predict treatment attrition. Based on the findings of this study, drop out management recommendations are made, as are implications for further research and ongoing practice.

## ORAL, JUNE 8 HALL SYDNEY

## Morning
*Open Papers: Complex traumatisation and comorbidity*

### The body remembers; examples of the mind body connection in survivors after severe traumatic experiences 10:00–10:15

#### A. Arnautovic Vive Zene Center For Therapy And Counseling, Tuzla, Bosnia and Herzegovina

Dismissal of trauma is never final and recovery is never complete. During and immediately after the traumatic event, organism, in a psychological sense, uses its existing defense mechanisms in order to survive. Especially remembered are experiences of severe physical trauma. The concept of mind body connection is not new. Many tried to explain it over the centuries. The human body, cell tissues, and organs, remembers and carries a base memory of perfect health common to the entire human race. Individuals are unaware of the link between their existing health conditions and symptoms with what was happening. The most common symptom among those who survived the detention camp and experience of rape is chronic pain syndrome. Psychological factors play a significant role in the onset, severity, exacerbation, or maintenance of the pain. Dismissal of pain occurs only when they revive the memory of pain. The pain can severely disrupt different aspects of daily functioning. Treatment of people with severe traumatic experience followed by painful syndrome is complex, time consuming, and it should be multidisciplinary. The combination of drug therapy and psychotherapy is necessary. Also important is the support of the community to the traumatized persons and their family members. Professionals who help people with severe traumatic experiences must not forget the personal reactions and feelings that may not have been worked through and those may be ‘blind spots’ that hinder the therapeutic process. The author wants to show the parts of psychotherapy sessions where there has been connecting memories and experiencing symptoms that clients have in the present and personal experiences of recognizing relived.

### Psychiatric and social impairment in refugee out-patients and psychiatric in-patients 10:15–10:30

#### S. Palic^1^, M. Lind Kappel^2^, M. Stougaard Nielsen^2^, A. Elklit^1^ and P. Bech^3^

^1^Danish National Centre for Psychotraumatology, University of Southern Denmark, Odense, Denmark; ^2^Clinic for PTSD and Transcultural Psychiatry, Aarhus University Hospital, Aarhus, Denmark; ^3^Psychiatric Research Unit, Frederiksborg General Hospital, Hillerød, Denmark

There are no commonly applied measures of psychiatric and social impairment in treatment seeking refugees in the West. Consequently, the level of impairment in this psychiatric group relative to other psychiatric groups in the West is unknown. In the present study, 448 consecutive outpatients from a Danish clinic for traumatized refugees were assessed with the Health of Nation Outcome Scales (HoNOS) over a 3-year period. Their pre- and post-treatment scores were compared to those of Danish psychiatric inpatients collected over a 10-year period. Diagnoses represented were affective, anxiety, and personality disorders, schizophrenia, dementia, and addiction. Despite being outpatients, the refugees had the highest levels of psychiatric and social impairment pretreatment. Post-treatment, the refugees further showed the smallest percent wise improvement. Consequently, better cooperation between the specialized clinics for refugees and other parts of the psychiatric and health care system is urged to meet the very complex needs of this population.

### Posttraumatic stress disorder in bulimia nervosa 10:30–10:45

#### R. Schumann, L. Tieghi and D. Ballardini Centro Gruber, Bologna, Italy

*Objective*: Comorbidity among eating disorders, traumatic events, and post-traumatic stress disorder (PTSD) have been reported in research with various prevalences due to differences in assessment, diagnostic criteria, and recruitment methods. The main objectives of this study were to explore the prevalence of PTSD and the relation with bulimia nervosa (BN) and to describe the nature of traumatic events experienced in a female outpatient sample. *Methods*: Two-hundred fifty-seven outpatient females with BN were assessed (EDI-II, EAT, TFEQ, GHQ and SCID-I/P) for comorbidity, anxiety disorders and PTSD. *Results*: From the 257 women with BN (age 25,81±6,83; BMI 20,98±4,01; duration 3,44±2,62 years) 18.2% (*n*=47) met DSM-IV criteria for PTSD. The BN with PTSD did not present significant differences in the eating disorders characteristics, the majority (95.7%) reported the first traumatic event before the onset of BN. The most common traumatic events reported were sexually related during childhood and adolescence (41%), 8.5% reported childhood obesity experienced as a traumatic event of their PTSD. The BN patients with PTSD referred significantly more multipulsivity (52%) than in BN patients without PTSD (22%). *Conclusions*: BN and PTSD do co-occur and traumatic events tend to occur prior to the onset of BN. It is necessary to assess trauma history and PTSD in BN and it might be effective to enlarge the interdisciplinary treatment program with a trauma therapy module for this subgroup of BN patients to address their PTSD-related psychopathology.

### Patients as partner on the trauma team to improve quality and safety of care processes 10:45–11:00

#### E. Van Der Schrieck De Loos CBO Dutch Institute for Healthcare Improvement, The Netherlands

Engaging the patient's perspective on care processes is crucial to enhance quality and safety of trauma care. Healthcare is teamwork and patients must be identified as a team member. Only patients participate during the entire care process. They can be a partner on the trauma team as they have an unique perspective on their care. Patients can therefore have an important role in preventing medical errors by providing information about their medication history or asking questions about their treatment. Current interventions are mostly based on patients themselves and need to be further developed, evaluated, and innovated while implementation is moving on. Interventions for healthcare professionals to engage patients has to be further developed. Engaging patients is only possible when initiatives are based on the relationship between patients and healthcare professionals. Active patient participation can only be achieved on a voluntary basis. Hence, it depends on the patient's willingness and ability to become a partner in trauma care. Healthcare professionals can, while keeping full responsibility for their patients, improve their care by using the patient's eyes and ears. This makes a good relationship with an active dialogue between healthcare professionals and their patients crucial. Optimizing this relationship requires education of both patients and healthcare professionals by raising awareness and using practical tools. To create a long term effect of the patient's role it is essential that incorporation of the patient's perspective is developed at the levels of the individual care process, healthcare organizations, the healthcare system, and laws and regulations. The session is based on the results of the international qualitative exploratory research report: *“The role of the client in patient safety. A necessity, not a desirability”* and consisted of an international literature review, web search, semi-structured interviews, and an expert meeting to discuss recommendations for implementation of the patient's role.

### Influence of comorbid depression on the course and on the prognosis of the posttraumatic stress disorder 11:00–11:15

#### G. Grbesa, M. Simonovic and M. Stankovic Medical School University Nis, Nis, Serbia

*Background*: Comorbidity of posttraumatic stress disorder (PTSD) and depression offers the possibility to explore broad spectrum of interactions of mood and anxiety disorders in several domains: in the domain of clinical presentation, course and prognosis as well as in the treatment effectiveness. *Method*: Totally 60 patients were divided into the experimental: PTSD-depression (30), and control: PTSD—only group (30). The assessment was made by means of the following intruments: SCID for DSM-IV, CAPS-DX, MADRS, and 17-items HDRS. The patients were evaluated the three sessions: initially upon treatment-seeking, after 6 months and after 2 years during the longitudinal follow-up. The data were analyzed using the methods of the descriptive statistics and of corellational and regressional analyses of the data. *Results*: Experimental PTSD-depression group is characterized by the increased emotional reactivity, more intense re-experiencing symptoms, and by more severe depressive cognitive symptoms cluster. The evolution and the clinical course reveal recurrence of the depressive episodes. The control PTSD-only group is characterized by the emotional numbing, affect restriction, and by evolution towards DESNOS or towards personality changes with prominent impulse control difficulties. *Conclusion*: The experimental PTSD-depression group shows more intense cognitive engagement and increased emotional reactivity in comparison with the control group. The subjects of the PTSD—depression group are more attainable to treatment, but also, to the potential risks of the triggering of the depressive episodes during any treatment modality, either by using medication or psychotherapeutic approach.

## Open Papers: Barriers to trauma care

### Does PTSD increase the risk of self-poisoning during out patient psychiatric treatment? 11:45–12:00

#### J. Siqveland^1^, E. Hauff^2^ and T. Ruud^1^

^1^Akershus University Hospital, Nordbyhagen, Norway; ^2^University of Oslo, Oslo, Norway

PTSD as comorbid diagnosis to depression or borderline personality disorder has in some, but not all, investigations found to be related to more severe pathology and worse clinical outcome. One aspect of a worse clinical outcome is self-destructive behaviors, such as self-poisoning. The present project investigates whether PTSD as a comorbid condition to major depressive disorder (MMD) and borderline personality disorder (BPD) is predictive of increased risk of self-poisoning. Using data from the electronic patient administration system at a large University Hospital in Norway, all patients receiving outpatient psychiatric care from three community mental health centers in the period 2006–2011 will be included. These data are linked to registry data of all self-poisoning episodes from the corresponding emergency medical department. All self-poisoning episodes during treatment at the community mental health centers are included in the further analysis. Based on clinician rated diagnosis, four comparison groups will be created (1) MDD, (2) MDD+PTSD, (3) BPD, and (4) BPD+PTSD. Based on previous research, we expect that the MDD+PTSD group will have a higher risk for self-poisoning than the MDD group, whereas there will be no difference in risk for self-poisoning between the BPD+PTSD and the BPD group. Using a case-control design, risk ratio for self-poisoning for the different diagnostic groups will be calculated. Results from these analyses will be presented.

### Crisis intervention in the acute phase after trauma: the client's subjective needs 12:00–12:15

#### B. Juen^1^, E. Mohr^1^, H. Siller^2^ and S. Nindl^1^

^1^Department of Psychology, University of Innsbruck, Innsbruck, Austria; ^2^Women's Health Centre of Innsbruck Medical University Hospital, Medical University Innsbruck, Innsbruck, Austria

The acute team offers support for relatives after acute loss of a loved one. A team of psychologists and social workers carries out this support. In a study on 426 cases of acute loss taken from the documentation of the acute team lower Austria risk factors, problematic acute reactions, as well as interventions and satisfaction with the interventions were analyzed. Additionally, we analyzed narrative protocols of 25 cases using qualitative content analysis. In the poster, the focus is set on the qualitative results. Results showed that the clients’ subjective needs to emphasize the importance of specific resources and interventions to enhance manageability, sensibility, and comprehensibility. The results showed that the clients’ most important resources were somebody who listened to them, the support from and to family, social integration, as well as personal resources. The most important interventions from the clients’ viewpoint were the giving of information, the enhancement of social resources, and the listening. As indicators of positive change, they saw positive emotions, ability to perform everyday routines, reduction of intrusive thoughts, and arousal as well as acceptance of the events.

### Comorbidity of depression and posttraumatic stress disorder 12:15–12:30

#### M. Simonovic^1^, G. Grbesa^1^, M. Radisavljevic^1^ and T. Milenkovic^2^

^1^Medical Faculty University of Nis, Nis, Serbia; ^2^Clinic for Mental Health, Clinical Center Nis, Nis, Serbia

*Objective*: The aim of this investigation is to determine the group of symptoms which are the most prominent in depression comorbid to PTSD and to compare delineated features with the similar features of the depressive episode which is a part of the primary major depressive disorder. The results were interpreted regarding the patterns of cerebral activity in PTSD and depression. *Method*: One-hundred twenty patients were divided in experimental (depression-PTSD) and control (depression-only) group, and evaluated using the following instruments: MADRS, HDRS-17, and QIDS-SR. The statistical analysis of the data was performed by mean of Student t-test and Mann -Whitney U test. *Results*: Symptoms which differed most significantly between the two groups were: On MADRS instrument inner tension, sleep disturbances trouble concentrating, lassitude, inability to feel, and pessimistic thoughts (p<0.001). On HDRS-17 instrument: early, middle, and late insomnia, agitation, work, and activities (p<0.001). On QIDS instrument: early, middle, and late insomnia, concentration, interest and, decision-making (*p<*0.001). *Conclusion*: By interpretation of the results obtained regarding the patterns of cerebral activity in PTSD and depression, we reached the conclusion that depressive symptoms which were followed by PTSD are not the results of the increased activation of the neural circuits by the two pathophysiological processes, but there is the case of differential engagement of neural networks in which stimulation from the brain structures responsible for generation of emotional input, increasingly arrives into the brain centers involved in the processing of emotional contents, in which in depressed individuals, the more intense and deeper emotional processing takes place causing more intense experience of emotional stimuli and bridging the connection from perceptual and cognitive contents up to affective and visceral center of the organism. That is why the depression comorbid to PTSD is so severe and difficult to treat and to live with.

### Barriers to mental health care in a high risk profession; a study on the Dutch police 12:30–12:45

#### N. Burger^1^ and B. Gersons^2^

^1^Impact, Arq Psychotrauma Expert Group, The Netherlands, ^2^Arq Psychotrauma Expert Group, AMC University of Amsterdam, Amsterdam, The Netherlands

*Introduction*: Police officers experience many traumatic events next to organizational and daily life stressors. This results in 25–37% mental health problems in Dutch police. These MH-problems interfere with work, like avoidant behavior when action is necessary, or using excessive violence, wrong judgments, and concentration problems. The police organization is often legally responsible for the prevention and treatment of MH-problems. *Method*: A mixed method study was conducted to establish the demand for mental health care, the accessibility of care, and the care actually delivered to Dutch police officers. Quantitative data on the use of mental health were gathered and qualitative data were collected from focus groups and laptop conferences on the access to and experience of MH-care. *Results*: Annually at least 8% of Dutch police officers receive mental health care. Many police officers with MH-problems however do not receive or avoid MH care. In our study, we encountered important barriers towards care. Police officers avoid to show and to talk about even minor MH-problems. They are afraid to be stigmatized as weak and not fit for the job. Also, officers are afraid that looking for MH care will interfere with job promotions. Moreover, people surrounding the officer who needs help, do not recognize the psychosocial problems or are hesitant to discuss these with the officer in question. In a blueprint delivered by Arq to the Dutch police, some solutions to break the barriers are listed and will be presented at the conference.

### A national study of the psychological impact of bank robbery with a randomized control group 12:45–13:00

#### M. Hansen and A. Elklit National Centre for Psychotraumatology, University of Southern Denmark, Odense, Denmark

*Background*: Despite numerous annual bank robberies worldwide, research in the psychological sequelae of bank robberies is limited. Thus, research needs to investigate the prevalence of acute stress disorder (ASD) and posttraumatic stress disorder (PTSD) in bank employees, whilst comparing how bank employees exposed to bank robbery differ from employees not exposed to bank robbery. *Objective and design*: We studied the prevalence of ASD one week after the robbery (*N*=458) and the prevalence of PTSD 6 months after the robbery (*n=*378) in a national Danish bank employees exposed to bank robbery. We also investigated several other forms of psychological sequelae and related factors in bank robbery victim for instance prior traumatic experience, anxiety symptoms, and general traumatic symptoms. The results were compared to a randomized control group of bank employees never exposed to bank robbery (*N=*303). *Results*: The estimated ASD rate was 11.1% (*n*=41), and the estimated PTSD rate was 6.2% (*n=*23). However, the ASD and the PTSD prevalence rates were limited by the avoidance diagnostic criteria (ASD without avoidance=14%, PTSD without avoidance=17.8%). Preliminary results indicate that the control group scored significantly lower than the ASD robbery group but surprisingly significantly higher than the PTSD robbery group on for instance general traumatization and anxiety. *Discussion and conclusions:* The results are discussed in relation to existing research and the effect of other factors such as prior traumatic exposure. A preliminary conclusion is that a bank robbery is a traumatizing event for employees, especially when disregarding the avoidance symptoms. This seems to be particularly pertinent in relation to the acute phrase following the bank robbery.

## Open Papers: Contesti traumatici complessi

### The simulacra of shame: the complexity of trauma in adoptive parenthood of second generation   15.15–15.30

#### Elena Acquarini Dipartimento di Scienze dell'Uomo, Università degli Studi di Urbino, Urbino, Italy

The silent suffering of the early and unprocessed abandon digs noisily even in the parental attitude, especially in adoptive possibility. Adopted children who in turn create a foster family found a potential traumatogenic dangerous living *on the side* of the parental adoptive child. Sometimes, these family structures, which have failed to define the perimeters of roles and that should provide elements to be able to give meaning to child experience, jump by choosing as the sole-language practicable symptom. Children become unwitting simulacra of the other person's shame, bare only in reflective-mode branch which is delivered in its entire unprocessing mode. The destructive power of what has not been addressed puts it bare helplessness and shame that continues its disorganizing work pushing the subject towards self-destruction and concealment. If so, when the traumatic experience had its best on individual resources and environmental, leaves in your baggage tools not effective that alter the reading ability of self and the world, discovering suddenly the wetsuit despair of these children. The trauma of orphan children transforms autoplastically and their own psyche temporarily renouncing to attempt to modify alloplastically the outside world as not yet known. From the parental attitude, shame hides deep sentiments of annihilation, impotence, and consequent liabilities for the usual defensive schemes cannot remain effective and the regression becomes a massive response to an impressive sense of unreality and a traumatic suspension.

References

Ardino V. (Ed.) (2011), *Posttraumatic Syndromes in Childhood and Adolescence*. Wiley-Blackwell.

Caretti V., Schimmenti A. (2007), *Prefazione. Il fallimento delle relazioni primarie e il trauma evolutivo*. In: Bifulco A., Moran P. (1998), *II Bambino Maltrattato*. Casa Editrice Astrolabio, Roma.

Ferruta A. (2004), *Esperienza clinica e ricerca empirica a confronto*. In: Alberto G.G. (Ed), *Il cambiamento nella psicoterapia di crisi*. Franco Angeli, Milano.

### The transmission of trauma inside survivor families: from Shoah to current conflicts and persecutions 15.45–16.00

#### Valentina Cassese “Carlo Bo” Dipartimento di Scienze dell'Uomo via Saffi, Università degli Studi di Urbino, Urbino, Italy

Theories of trauma transmission offer explanatory models of the suffering of the second and third generations of the survivors of conflict and persecution horrors then trying to understand the mechanisms by which individuals born also in times and places far away from those traumatic events experience the effects *as if* they had lived through the experience directly. This work summarizes the debate concerning the mechanisms that allow the transmission between the generations and psychological difficulties registered in each of them. Indeed, only a relatively small number of survivors of mass violence were present with mental illness, such as to require intense psychiatric intervention, most of them suffer from mental problems of lesser gravity but long term and these sufferings seem to be perpetrated in some way on their descendants. Particularly, by examining the contribution of Dr. Wardi, it has been observed and proposed that the need for therapeutic support for survivors’ subsequent generations, there is a deep link between this status and the inability to implement the natural process of identification. The second generation seems to have the task of filling the huge vacuum left in the lives of their parents, representing for the latter a ring of conjunction between past and present, a *candle of memory* lit, so that the remembrance can remain.

**References**

Wardi D. (1992), *Le candele della memoria: i figli dei sopravvissuti all'Olocausto*, Sansoni (1999).

Zajde N. (1993), *Enfant de survivant. La transmission du trumatisme chez les enfantes Juifs survivantes de l'extermination nazie*, Edition Odile Jacob, Paris.

Nicolò Corigliano A. M. (1996), Il transgenerazionale tra mito e segreto. *Interazioni*, 1.

### Arrivals: tell the migration with images 16.00–16.15

#### Diego Manduri Istituto di Terapia Familiare di Bologna

Starting from the difficulty of giving support and consultation to migrants on their stories and pain, a technique of storytelling that departed from the suggestions of the image has been created and tested. By integrating two aspects of training, the systemic-relational thinking and the use of images, at the Institute of Family Therapy of Bologna, we created a narrative tool *ad hoc* using “The Arrival”, a graphic novel by Shuan Tan. The images are highly suggestive views, choices, and then narrated within a group of migrants who have shared a path of teaching. We tested “Arrivals” in five classes of an Italian course for a total of about 100 migrants. The experimental results confirm that the image facilitates recall, recount, accept, and understand complex, rich, and painful stories.

## POSTERS, JUNE 8

## Psychobiology and Trauma

### The roles of peritraumatic heart rate and acoustic startle reflex in predicting traumatic memory processing

#### C. Chou^1^, R. La Marca^2^, A. Steptoe^3^ and C. Brewin^1^

^1^Clinical Psychology, University College London, London, UK; ^2^Klinische Psychologie und Psychotherapie, Psychologisches Institut, Universität Zürich, Zurich, Switzerland; ^3^Institute of Epidemiology and Health Care, University College London, London, UK

*Objectives*: Heart rate (HR) has been studied as an index of cognitive processing and stress defense. In a previous study with the trauma film paradigm, low peri-film HR was found to predict greater intrusion and regarded as indicative of dissociation. This study attempted to replicate this finding and directly examined the correlation between HR and dissociation. In addition to frequency, the vividness of intrusion was also investigated. In addition, differences in cardiac startle response to a sudden loud noise were related to the psychophysiological reactions to traumatic cues. *Methods*: Participants showing and not showing startle were categorized into the startle (n=14) and non-startle group (n=19), respectively. All participants were exposed to the trauma film with HR, state dissociation, and fear being assessed pre-, peri-, and post-film. The frequency and vividness of intrusion were recorded with an intrusion diary for 7 days. *Results*: The non-startle group showed higher trait dissociation and higher HR across all phases than the startle group. Overall, HR decreased by 0.63 bpm peri-film. For the non-startle group, lower peri-film HR was associated with higher fear and state dissociation. Moreover, the more the peri-film HR decreased, the less vivid were the intrusions. However, for the startle group, these associations were not significant. A moderating effect of group was shown in the relationship between peri-film HR decrease and intrusion vividness. *Discussion*: This study was the first to examine how startle moderates the relationship between HR and intrusion. The findings suggested interesting individual differences in stress defense style. Moreover, the group discrepancy in the psychological correlates of HR deceleration suggests the importance of considering individual differences. Finally, this study is one of the few to examine intrusion vividness. The findings suggest different mechanisms underlie intrusion frequency and vividness.

### Neural correlates of memory dysfunctions in PTSD: preliminary findings of a systematic review and a mixed image/voxel-based meta-analysis

#### W. El-Hage^1^, Y. Quidé^2^, J. Radua^3,4^ and M. Olff^5^

^1^INSERM UMR U930, Université François Rabelais de Tours & Clinique Psychiatrique Universitaire, CHRU de Tours, Tours, France; ^2^INSERM UMR U930, Université François Rabelais de Tours, Tours, France; ^3^Institute of Psychiatry, King's College London, UK; ^4^FIDMAG, CIBERSAM, Sant Boi de Llobregat, Spain; ^5^Department of Psychiatry, Anxiety Disorders, Academic Medical Centre, University of Amsterdam, Amsterdam, The Netherlands

*Background*: Memory is a central component of posttraumatic stress disorder (PTSD) as defined by its three symptom clusters. Structures part of the “fear network” show abnormal activities in PTSD (i.e., hippocampus, medial prefrontal cortex/anterior cingulate cortex (mPFC/ACC), and amygdala) and are also involved in memory processes. *Methods*: We conducted a systematic review and a mixed image/voxel-based meta-analysis on neural correlates of memory dysfunctions in PTSD on the past 20 years of neuroimaging literature. A total of 26 publications (*Global Memory*; PTSD: n=368; Controls: n=291) comparing PTSD patients to their control groups (Healthy and/or Exposed), fulfilled the study criteria: 21 used long-term memory (LTM) paradigms (PTSD: n=266; Controls: n=234) and 9 used short-term memory (STM) paradigms (PTSD: n=123; Controls: n=102). *Results*: When performing a memory task (*Global Memory*), PTSD patients had significant greater activations than controls in left supramarginal gyrus (SMG, BA40) and diminished activations in bilateral insula/inferior frontal gyrus (IFG, BA13/47), mPFC/ACC, and left medial/superior frontal gyrus (BA8/9). During LTM paradigms, they exhibit significant greater activations in right IFG (BA44), as well as decreased activation in right superior temporal gyrus (BA21/22/38), right insula (BA13), left SMG/Inferior parietal lobule (BA40), and precuneus/posterior cingulate cortex (BA7/23/31). During STM tasks, patients show significant less activation in left IFG (BA47), right motor area (BA4/6), and left precuneus/angular gyrus (BA39). *Conclusion*: In PTSD, the dysfunction in the insula/IFG is common to all memory paradigms. This structure, together with the ACC, composes the salience network (SN). The results provide evidence that dysfunctions of the SN can disable PTSD patients to cognitively engage in a cognitive task (switch from resting state to task).

### The role of sleep in emotional memory processing in PTSD patients

#### M. De Boer^1^, M. J. Nijdam^2^, W. F. Hofman^1^, M. Olff^2^ and L. M. Talamini^1^

^1^Brain and Cognition group, Department of Psychology, Cognitive Science Center Amsterdam, University of Amsterdam, The Netherlands; ^2^Department of Psychiatry, Centre for Psychological Trauma, Academic Medical Centre at University of Amsterdam, The Netherlands

Sleep appears to play an important role in emotional memory processing and emotional coping. Disturbed sleep (nightmares and insomnia) is one of the key symptoms of posttraumatic stress disorder (PTSD) and may play an important role in the aetiology and/or maintenance of PTSD. Polysomnographic studies in PTSD patients have reported mainly on changes in REM characteristics and arousal regulation. However, little is known about the relation between sleep disturbances and emotional memory processing in PTSD. A previous sleep study in healthy subjects suggests the occurrence of adaptive changes in sleep architecture after emotional experiences, which benefit emotional housekeeping and the attenuation of emotional responses towards negative emotional experiences (manuscript under submission). The current controlled patient study assesses the impact of an induced, emotionally distressing experience on sleep parameters in PTSD patients, including the distribution of sleep stages, REM sleep-related variables, and EEG power spectral parameters. In addition, we will analyse how sleep changes in response to the stressor relate to emotional attenuation over sleep. The main experimental groups are traumatized police officers and veterans with PTSD (N=25) and without PTSD (N=25). We will also include a control group of non-trauma exposed controls (N=25). The experimental set up involves presentation of neutral or distressing film fragments in the evening, followed by polysomnography (EEG - F3, F4, C4, O2- referenced to linked A1+A2; EOG; EMG; ECG; respiratory signals; limb movements) of undisturbed, whole night sleep, and cued recall of film content on the next evening. The order of the film conditions is counterbalanced across subjects. Emotional state and physiological measurements (ECG, respiratory effort, GSR, and plethysmogram) are assessed before and after film viewing and cued recall. Physiological signals are recorded during the film and stills as well. Preliminary results will be presented and discussed.

### Physiological reactivity of individuals with PTSD and support during a trauma oriented social interaction with a significant other: a gender-comparative analysis

#### S. Guay^1^, N. Nachar^2^, M. E. Lavoie^3^, A. Marchand^4^ and K. P. O'Connor^3^

^1^School of Criminology, University of Montreal, Canada; ^2^Department of Psychology, University of Montreal, Canada; ^3^Department of Psychiatry, University of Montreal, Canada; ^4^Department of Psychology, University of Quebec in Montreal, Canada

Overt behavioral support processes and physiological responses are dimensions that have been much overlooked in the exploration of the links between social support and posttraumatic stress disorder (PTSD). A multi-method strategy was developed to study physiological reactivity during a supportive interaction with a significant other. The mean and variability of heart rate (HR) of 52 participants with PTSD (40 women) were respectively measured in four phases: (1) a 2-minute resting baseline, (2) a-10 minute neutral interaction with the significant other, (3) a 15-minute active interaction with the significant other evoking the impacts of PTSD on their lives, and (4) a 2-minute recovery phase. Our results revealed a significant increase in HR responses during the trauma-oriented discussion. This HR response increase was significant in comparison to all other control periods, i.e., the preceding neutral discussion with a significant other as well as the initial and final resting periods (*p*<0.01). Men and women from our sample showed similar HR mean and variability during each phase. Although there was no link between the intensity of PTSD symptoms (measured with the CAPS) and women's HR at all phases, significant positive correlations were found for men during phases 1, 3, and 4 (*r*s>0.62, *p*s<0.05) with HR variability. During phase 3, the more the men expressed emotions to their significant other, the less HR variability was observed (*r*=0.40, *p<*0.05). Our findings suggest that PTSD symptoms are more strongly associated with the physiological reactivity of men before, during, and after an interaction with a significant other about their trauma. Clinical strategies addressing these issues will be discussed.

## Miscellaneous

### Development and validation of a scale to measure trauma-related guilt and shame

#### K. Derks^1^, W. Van Der Veld^1^, G. Näring^1^, E. Becker^1^ and J. Krans^2^

^1^Behavioural Science Institute, Radboud University Nijmegen, The Netherlands; ^2^University of New South Wales, Sydney, Australia

Although scholars agree that emotions of guilt and shame are critical in the development of posttraumatic stress disorder (PTSD) symptoms after a traumatic event, measurement instruments of these emotions in relation to trauma are still limited. Additionally, the existing scales principally measure trauma-related guilt, and the emotion of shame is often not included, even though a body of clinical research on psychological trauma indicates that the emotion shame is important in the development and course of PTSD symptoms. Moreover, the existing measures fail to recognize that these moral trauma-related emotions do not only have a cognitive component but also a behavioral reaction. As guilt is essentially a constructive moral emotion, associated with feelings of responsibility and agency, it results in a desire to repair what one has possibly done wrong. However, this repair behavior is not part of the existing instruments that measure trauma-related guilt. Just like guilt, shame has, next to the cognitive component (negative self-evaluations, ‘'I am a bad person’’), its own behavioral element: withdrawal (e.g., hiding). Shame makes one want to withdraw and to avoid dealing with the consequences of traumatic events. We addressed these issues by developing and validating a new scale that measures both trauma-related guilt and shame experiences. The scale contains two guilt subscales that assess negative behavior-evaluations (cognitive) and the tendency to repair (behavioral) following a traumatic event, and two shame subscales that measure negative self-evaluations and withdrawal behavior following a traumatic event. Our scale's ability to distinguish these two classes of responses (cognitive and behavioral) and its ability to include both trauma-related guilt and shame represents a vital advantage of the scale over existing instruments. Consequently, it has the potential to be an important tool for identifying trauma-related guilt and shame.

### The degree of dissociative and posttraumatic stress in oncology

#### A. Gallo Dipartimento di Scienze dell'Uomo, Università degli studi di Urbino “Carlo Bo”, Italy

A traumatic event is considered a stressful event that overwhelms the resilience of the subject. A traumatic event can be an isolated incident or repetitive causing a chronic trauma in the patient. The shift to the subjective experience of trauma led to a definition of traumatization as an individual response at cognitive, affective and defensive level. In this sense, an event becomes “traumatic” according to the way in which the subject experiences it in his or her inner world, i.e., in relation to the quality of his or her personal reality. Traumatic experiences act on splitting up higher integrative functions and this creates the existence of dissociative phenomena and psychopathological disorders such as posttraumatic stress disorder (PTSD). The disruption resulting from psychological trauma however does not seem to be a defense of the mind, but rather a side effect that has grave repercussions on the ability of the individual to regulate emotional, and metacognitive capabilities in relation to one's own identity. The seriousness of the dissociative disorder and PTSD when associated with traumatic histories of development can worsen the prognosis if they are present as an illness in combination with other disorders. In fact, if we try to analyze a dramatic context such as cancer, it is noted that the communication of a poor diagnosis can be characterized as a critical time for the development of this phenomena. In this situation, it seems to be essential for a specific intervention to reduce symptoms and return the patient to a normal level of functioning in order to be able to manage the organic pathology.

References

Liotti, G., & Farina, B. (2011). *Sviluppi traumatici. Eziopatogenesi, clinica e terapia della dimensione dissociativa*. Milano: Raffaello Cortina.

Lingiardi, V. (2004). *La personalità e i suoi disturbi. Lezioni di psicopatologia dinamica*. Milano: Il Saggiatore.

National CancerInstitute: http://www.cancer.gov/cancertopics/pdq/supportivecare/post-traumatic-stress

### Psychometric properties of the Hungarian versions of the Impact of Event Scale-Revised and the Impact of Future Events Scale

#### K. Fodor^1,2^ and D. Perczel Forintos^1^

^1^Department of Clinical Psychology, Semmelweis University, Budapest, Hungary; ^2^Doctoral School of Mental Health Sciences, Semmelweis University, Budapest, Hungary

A study that investigated the psychometric properties of the Hungarian versions of the Impact of Event Scale-Revised (IES-R; Weiss & Marmar, 1996) and the Impact of Future Events Scale (IFES; Deeprose & Holmes, 2010) in a sample of healthy subjects is presented. The IES-R is a 22-item self-report measure that assesses subjective distress along three subscales after traumatic events. The previously available and validated Hungarian version of the Impact of Event Scale (Horowitz et al., 1979) is updated and retranslated to fully assess all posttraumatic symptoms. The IFES is a 24-item scale that was developed based on the IES-R and assesses the impact of intrusive, prospective, personally relevant imagery of events occurring to the respondent in the near future. The two scales are tested in one time with the purpose of exploring possible connections between effects of past events and the impact of future events on the individual. The psychometric properties of the Hungarian versions of the scales were tested in a sample of 200 healthy subjects. The internal consistency, test–retest reliability, convergent and divergent validity, factor structure, as well as information about the translation process are discussed. The process of the analysis of the convergent and divergent validity raises transdiagnostical questions.

### H.O.W.? Now!: Logotherapy and a rapid strategic integrated approach to treating PTSD with or without SUD

#### R. Garman Walden University, Minneapolis, USA

In an interview from the documentary film “RESTREPO,” after returning in 2006 from the Korengal Valley region of Afghanistan, U.S. Army Staff Sergeant Joshua McDonough said, “They're gathering intel right now basically on how to deal with us, because they haven't, there's no … really … research, or intel, on how to treat us right now. They haven't had to deal with people like us, since WWII and Vietnam, dealing with guys who are coming back with 15-month deployments with as much fighting as we went through.” According to the George Washington University Face the Facts Initiative, about 300,000 veterans do date, one in five of the wars in Iraq and Afghanistan, have been diagnosed with posttraumatic stress disorder (PTSD). Currently, atleast 30,000 U.S. military veterans are ineligible for disability benefits because there were found to have a personality disorder, something the military says is a pre-existing condition. In April 2012, General Eric Schinseki, secretary of the Department of Veterans Affairs, announced that an increase in 1,900 mental health professionals would be reduced to 1,600. From current statistics regarding the dramatic increase in military suicides and the diagnosis of PTSD, and a variety of other mental health disorders including substance use disorders (SUD), it is clear that the shortage of professionals may just be the tip of the iceberg.

## Cultural Issues and Trauma

### Holocaust survivors and their post-war relationships: women's coping, healing, and interpersonal bonds

#### G. Mapel New York University, New York, NY, USA

There is an unidentified discrepancy in the literature. One body of literature suggests that social support aids recovery from trauma (Tedeschi & Calhoun, 2004), while another asserts that trauma leads to difficulties in establishing and maintaining relationships so that access to support is restricted (Krystal, 2006). Thus, the two bodies of literature, taken together, reflect a *paradox* first identified and referred to as a “*bind*” by Banks (2006): trauma survivors need social support to heal; yet, due to their exposure to trauma, some survivors are left relationally challenged. This paradox is exacerbated in the case of *complex trauma*. Research supports the presence of a traumatic syndrome that differs from posttraumatic stress disorder (PTSD) in terms of severity and complexity of symptom presentation (Ford, Stockton, Kaltman, & Green, 2006; Ford & Kidd, 1998). Herman (1992) coined the term “complex post-traumatic stress disorder” (p. 119) to capture this more severe and complex presentation. People with complex PTSD suffer in the interpersonal realm. It is not just that complex trauma is (1) severe in nature and (2) often occurs over an extended period of time, but that (3) *it is trauma inflicted upon individuals by human perpetrators* that leads to its devastating and long-lasting effects, including impaired relations with others (Ford, Stockton, Kaltman, & Green, 2006). Survivors of the Holocaust have endured the extreme end of the complex trauma spectrum.

### Ethnic minority youth survivors of the Utøya-massacre, and their sense of belonging in the Norwegian society in the aftermath of 7/22

#### M. Aadnanes and M. Hauge Norwegian Centre for Violence and Traumatic Stress Studies (NKVTS), Violence and Trauma - Children and Youth Unit, Oslo, Norway

*Background*: On 22 July 2011, the Norwegian Labor Party's youth organization was attacked during their annual gathering at the small island of Utøya. The terrorist action was an attack on the government's immigration policy and the multicultural Norway. The perpetrator's intention was to start a war against Islam and against multiculturalism. His goal was a monocultural Norway. Thus, the politically active youth from ethnic minority groups who was at Utøya did not only represent the “liberal immigration policy,” they were also a manifestation of it. The study is a part of the larger ongoing study: “The terrorist attack: Experiences and reactions among Utøya survivors,” conducted at the Norwegian Center of Violence and Traumatic Stress Studies. *Aim*: The purpose of the study is to explore ethnic minority youths’ sense of belonging in the Norwegian society in the wake of the terror attack at Utøya. In the aftermath of the attack, there has been a heated debate in the media about immigration and integration policies in Norway. Among the 325 participants in the first round of data collection, 11.3% (N=36.7) had immigrant background. In the second phase of data collection, these were asked about whether their sense of belonging in the Norwegian society had changed in the wake of the terror attack, and if so, in what way. *Method*: Open-ended, qualitative questions about belonging were included in the semi-structured interviews with survivors of ethnic minority background 12 months after the attack. Narratives about experiences of belonging will be subject of qualitative content analysis. The content is currently analysed, and results will be presented at the conference.

### Victimization and PTSD in a Greenlandic youth sample

#### S. Karsberg, M. Lasgaard and A. Elklit Danish National Center for Psychotraumatology, University of Southern Denmark, Odense, Denmark

*Background*: Despite a growing number of studies and reports indicating a very high and increasing prevalence of trauma exposure in Greenlandic adolescents, the knowledge on this subject is still very limited. *Methods*: In a Greenlandic sample from four different schools in two different minor towns in Northern Greenland, 269 students, aged 12 to 18 (M=15.4; SD=1.84) were assessed for their level of exposure to 20 potentially traumatic events (PTEs) along with the psychological impact of these events. *Results*: Of the Greenlandic students, 86% had been directly exposed to at least one PTE and 74.3% had been indirectly exposed to at least one PTE. The mean number of directly experienced PTEs was 2.8 and the mean number of indirectly experienced PTEs was 3.9. The most frequent direct events recorded were death of someone close, near drowning, threatened to be beaten, humiliation or persecution by others, and attempted suicide. The estimated lifetime prevalence of PTSD was 17.1%, whereas another 14.2% reached a subclinical level of posttraumatic stress disorder (PTSD) (missing the full diagnosis by one symptom). Following exposure, girls were three times more likely to suffer from PTSD compared to boys. Education level of the father, type of school, living in a single parent household, and being exposed to multiple direct and indirect PTEs were significantly associated with an increase in PTSD symptoms. *Conclusion*: The findings indicate that various types of PTEs that Greenlandic adolescents are exposed to have the potential to result in substantial mental health problems. Furthermore, the findings indicate that Greenlandic adolescents are more exposed to certain specific PTEs than adolescents in similar studies from other nations. This study revealed that Greenlandic girls are particularly vulnerable toward experiencing PTEs. Indeed, in general, girls reported more experiences of direct and indirect PTEs. Furthermore, girls reported being more commonly exposed to specific types of PTEs compared to boys.

### Project TIC-Talk: tailoring trauma informed care to lesbian, gay, bisexual, transgender, and questioning youth

#### I. Seilicovich and S. Strahl The Village Family Services, North Hollywood, CA, USA

Childhood trauma has been proven to have detrimental effects into adulthood oftentimes resulting in mental and physical health challenges as well as substance abuse (Felitti et al., 1998). Research indicates that lesbian, gay, bisexual, transgender, and questioning (LGBTQ) youth are at higher risk for trauma and face greater psychosocial challenges compared to other teens (GLSEN, 2009). Effective intervention is critical to maximizing outcomes for trauma-exposed youth, and trauma informed care is a “seminal concept in emerging efforts to address trauma in the lives of children” (Hodas, 2006, page 6). Trauma-informed care (TIC) uses a strengths-based approach to address trauma and promote resiliency (Hodas, 2006). There is currently a gap in tailoring TIC to LGBTQ adolescents, in spite of their increased exposure to traumatic events. To fill the gap, The Village Family Services in Los Angeles, California, developed TIC-Talk, a replicable, single-session training specifically for providers working with LGBTQ trauma-exposed youth. The evidence-supported lessons of TIC-Talk include concepts and theory as well as concrete steps required to diffuse this innovation. The information provided generalizes to both clinical and non-clinical settings including schools, community-based organizations, and juvenile justice facilities. Evaluation results indicate an increased understanding and implementation of TIC.

References

Felitti, V. J., Anda, R. F., Nordenberg, D., Williamson, D. F., Spitz, A. M., Edwards, V. & Marks, J. S. (1998). Relationship of childhood abuse and household dysfunction to many of the leading causes of death in adults. *American Journal of Preventive Medicine*, *14*(4), 245–258.

GLSEN, 2009. From Teasing to Torment: Survey Results Demonstrate the Severity of Bullying and Harassment in North Carolina Schools. *Gay, Lesbian, & Straight Education Network (GLSEN)*.

Hodas, G. R. (2006). Responding to childhood trauma: The promise and practice of trauma informed care. *Pennsylvania Office of Mental Health and Substance Abuse Services*, 1–77.

## Responding to Disasters

### Psychosocial crisis management in CBRN incidents: recommendations for a hospital staff training curriculum

#### S. Ludwig, G. Zurek, D. Wagner, K. Cummings and R. Bering Center of Psychotraumatology, Krefeld, Germany

*Introduction*: The risk of chemical, biological, and radioactive and nuclear (CBRN) accidents and attacks has grown in the past several years. Studies have shown that CBRN incidents have an impact on population mental health. However, it is clear that even small-scale CBRN incidents can cause psychological stress that affect disaster management. For this reason, the European commission supports international collaboration in CBRN risk management. *Methods*: According to our survey, hospitals are often not prepared for such incidents. Based on the current knowledge on stress response reactions in crisis management, we examined the differences between general disaster situations and stress responses in CBRN incidents. Pilot trainings and workshops were conducted in Berlin, Krefeld, and Madrid. *Results*: We created a model that clarifies the interface between stress responses and CBRN incidents, and focuses on differentiated knowledge about CBRN specialties. Our CBRN stress response model focuses on the psychological impact as a framework for addressing the emotional, cognitive, and behavioral effects. Via a consensus process, we defined recommendations on how to prepare hospital staff on psychosocial care assistance in case of CBRN incidents. We conclude that psychological models are needed to understand the difference between CBRN and other major incidents. We recommend implementing CBRN training for nursing staff and physicians in hospitals as a regular part of the training curriculum.

### Posttraumatic growth following rape and sexual assault

#### N. B. Hansen^1^, A. H. Jensen^2^ and A. Elklit^1^

^1^University of Southern Denmark, National Research Centre for Psychotraumatology, Odense, Denmark; ^2^Aarhus University Hospital, Sexual Assault Centre, Aarhus, Denmark

Posttraumatic growth is an increasing popular field, which emphasizes the potential developmental possibilities after a trauma. Posttraumatic growth has been defined as the experience of positive change that occurs following highly challenging life crises. It is supposed to be manifested as an increased appreciation for life in general, more meaningful interpersonal relationships, an increased sense of personal strength, changed priorities, and a richer existential and spiritual life. A number of concerns have been raised pertaining method and normative pressure on clients. During the last 10 years, the Danish rape crisis center in Aarhus has gathered information from victims of rape and sexual assault in relation to the victims’ qualitative experience of changes in life perspective and trauma-specific learning following the traumatic experience. We will present data from 350 Danish victims of rape or sexual assault on their experiences of changes after the incident. We have conducted a qualitative analysis of positive and negative life changes three months post-assault. Also, we have conducted a longitudinal study of positive and negative life changes 3, 6, and 12 months post-assault exploring the associations between life changes and psychological well-being. The data are currently being processed, but preliminary results show that only 1/7 of the participants report a positive change three months following the assault. Furthermore, the experience of positive change three month following the assault is significantly associated with lower levels of PTSD symptoms. We hope that the results will be able to contribute to the discussion concerning the concept and methodology of posttraumatic growth following rape.

### Coordinating research after the 2011 Norway terrorist attacks

#### N. O. Refsdal The Norwegian Research Ethics Committees, Oslo, Norway

How can we learn about the causes and effects of disasters without adding to the trauma of survivors, the bereaved, and personnel involved? This poster will present the coordinating function that has been set up after the terrorist attacks in Norway in 2011 with this explicit ambition in mind. The terrorist attacks in Norway on 22nd July in Norway left 77 people dead, most of them youths, several hundreds wounded, and an entire nation in shock and grief. A small, peaceful country marked by openness and trust saw its the government district in smoke and ruins, and some of its most idealistic and politically engaged young people callously massacred. A number of project in disciplines ranging from trauma medicine via psychology and the social sciences to the humanities has raised a broad array of research questions regarding causes, effects, the response of institutions, and the public at large. The plethora of possible angles raised the question of shielding those directly affected from the possibility of further research-induced traumatization. The Norwegian Research Ethics Committees were given the task of coordinating research where those directly affected participate. The primary objective is to safeguard the interests of those affected by the attacks. The tasks are:

Monitoring the load on the informant group
  Maintaining an overview of ongoing and planned research activities
  Contributing to the exchange of information between researchers
  Building networks and creating meeting places

This poster will introduce the setup of the function, reflect on the work and the lessons learned so far, and introduce plans for the way forward. The purpose is to share the experiences made and to invite the conference participants into a discussion about the concept of such a coordinating effort.

### Complexity for residents in Fukushima: Forced migration, evacuation decision, and discrimination after the nuclear power plant accident

#### M. Oe and M. Maeda Kurume University, Kurume, Japan

Still ca. 150,000 Fukushima residents are leaving their homes. They are worrying about the radiation levels in space and their radiation exposure, ceaselessly after the Fukushima Daiichi power plant accident. For example, although it was already known that radioactive substance were not released in a circle and strongly affected by geographical conditions, the evacuation zone was settled only within 20 km from the power plant at March 2011. Afterward, Japanese government recognized that there were high radioactive areas outside 30 km radius. This zone was called “planned evacuation zone” and 7,000 residents (including 2,100 voluntary evacuees in advance) were forced to leave at April 2011. This means thatinhabitants in this area had exposed high radioactivity without official warning for one month. They show anger even against the local people who live nearby the power, because some local governments have reaped a high profit margin for about 40 years. The parents have guilty to their small children. On the other hand, within 30 km zone, this was set up as the emergency evacuation preparation zone, were relaxed at September 2011. However, after the one year of this notice, only 10 % of inhabitants returned, due to lack of infrastructure construction and anxiety for the potential health risk in the long term. It might seem a strange phenomenon that only few people decided to relocate permanently outside Fukushima. This is partly because they are discriminated as “radioactive material-contaminated citizens” by others. Shigemura et al. reported about discrimination among TEPCO workers (JAMA, 2012). Discrimination against residents in Fukushima was also reported. In this poster presentation, we will try to focus on migration, discrimination and thecomplexity of their feelings and emotions according to the interviews of staffs in a psychiatric hospital in Minami-soma city.

## The Spectrum of Trauma-Related Disorders

### The effect of time perspective and of the emotional regulation difficulties on the PTSD symptoms among substance abuse Inpatients

#### M. Almeida^1^ and J. Rocha^2^

^1^Universidade Portucalense Infante D. Henrique, DCEP, Porto, Portugal; ^2^Instituto Superior de Ciências da Saúde - Norte, CESPU-UnIPSa-CICS, Porto, Portugal

Given the clinical relevance of the co-occurrence of post traumatic stress disorder (PTSD) among substance abuse inpatients, as well as the fact that PTSD is frequently underdiagnosed in this population, becomes relevant to research connections between factors that may be implicated in PTSD. Furthermore, recent publications highlight the relevance of time perspective in PTSD treatment strategies. Also, recent research suggests that emotion regulation difficulties may contribute to the development, maintenance, and exacerbation of PTSD among substance abusers. We aim to assess the importance of the studied constructs in order to integrate them, if justifiable, in the therapeutic program treatment. Sample consists of 72 substance abuse inpatients being treated in a therapeutic community, who received a questionnaire composed by a socio demographic section and the Portuguese versions of the Zimbardo Time Perspective Inventary—Revised, Trancendental Future Time Perspective Scale, Temporal Perspective Inventory—Negative Future Subscale, Difficulties in Emotin Regulation Scale and Impact of Event Scale—Revised. The frequency of participants with IES-R results above the cutoff value (35) was 71%. Time perspective dimensions, in particular, past perspectives, on stepwise multiple regression predict 35.5% of IES-R. Furthermore, emotional regulation difficulties have also revealed of high importance, Emotional Clarity and Strategies model has *R* ^2^=.343. In addition, several significant correlations between traumatic stress, emotional regulation difficulties, and time perspective dimensions are observed. Screening PTSD should be integrated in routine assessment and both time perspective and emotional regulations difficulties are relevant when defining treatment plans. The present findings support the existence of pervasive effects on the way patients consider their past experiences.

### Complex posttraumatic stress disorder presenting as somatization disorder

#### V. Sar^1^, O. Taycan^2^ and C. Celik^3^

^1^Department of Psychiatry, Clinical Psychotherapy Unit and Dissociative Disorders Program, Medical Faculty of Istanbul, Istanbul University Istanbul, Turkey; ^2^Adult Psychiatry Unit, Tokat State Hospital, Tokat, Turkey; ^3^Department of Psychology, Faculty of Arts and Sciences, Mus Alparslan University, Mus, Turkey

This study sought to determine the correlates of somatization disorder among a group of women recruited from a semi-urban and rural area in eastern Turkey. Dissociative Disorders Interview Schedule, Posttraumatic Stress Disorder (PTSD) section of the Structured Clinical Interview for DSM-IV, Dissociative Experiences Scale, Beck Scale for Suicidal Ideation, Hamilton Depression Rating Scale, Childhood Abuse and Neglect Questionnaire, and a Checklist for PTSD criterion A Traumatic Events were administered to participants with somatization disorder and 40 non-clinical controls recruited from the same region. Exposure to traumatic events of any type was high in both groups. However, women with somatization disorder reported criterion A traumatic events and/or childhood abuse and/or neglect more frequently than the comparison subjects (90% and 60% reported at least one type of trauma, respectively). Current depressive disorder (N=33, 77.5%), (N=22, 55%), current PTSD (N=12, 30%), dissociative disorder (N=11, 27.5%), borderline personality disorder (N=6, 15%) were more frequent in the somatization disorder group compared to the controls. Childhood emotional (25%) and physical abuse (20%), and emotional neglect (30%), suicide attempts (22.5%), and self-mutilative behavior (20%) were reported significantly more often in the somatization group. Interestingly, 37.5% of the somatization group reported at least one type of extrasensory/supernatural experience (including possession), whereas none of the controls did. In this group of women with endemically high exposition to traumatic events in childhood and adulthood, the high number of somatic complaints represented a complex PTSD covering wide psychiatric comorbidity rather than merely a somatization disorder.

References

Sar, V., Akyüz, G., Öztürk, E., & Alioglu, F. (in press). Dissociative depression among women in the community. *Journal of Trauma and Dissociation*.

Sar, V. (2011). Developmental trauma, complex PTSD and the current proposal of DSM-5. *European Journal of Psychotraumatology*, 2, 5622, doi: http://dx.doi.org/10.3402/ejpt.v2i0.5622

### Stressors and anxiety in pediatric patients with somatization

#### A. Bogdanic and M. Grubic University Hospital Centre, Zagreb, Croatia

Somatization refers to the expression of psychological distress through somatic symptoms. In order to help a person with somatization, it is important to identify the source of his/her psychological distress. The aims of this study are to identify the main subjectively perceived stressors in children with somatization and to explore the relationship between somatic symptoms, anxiety, and number and intensity of those stressors. Research was made at the Department of Pediatrics, University Hospital Centre Zagreb. Participants were all children (14 boys and 46 girls) aged from 10 to 18 years referred to pediatric psychologist due to somatic complaints of an unexplained organic origin in the period from May to December 2012. Participants filled in anxiety questionnaire (SKAD-64) and sentence completion test. Based on the sentence completion test and clinical interview, main stressors were identified and participants rated each of these stressors on a scale from 1 to 5. In 36% of participants the main stressor was school, in 21% family relationships, in 16% relationships with peers, and 18% highlighted their somatic symptoms as a main source of stress. In this sample, 43% of children had heightened level of anxiety with17% in a clinical range. We found significant positive correlation between anxiety score and number (r=0.351, p=0.01) and overall intensity (r=0.363, p=0.01) of stressors. No significant difference in anxiety, number, and overall intensity of stressors was found regarding the type of symptoms (headache, syncope, cardiac, gastrointestinal). Our results showed a positive relationship between anxiety and number and intensity of stressors in children with somatization. Since stress is an important factor in development of somatization, it is important to identify its sources in order to help our patients develop more effective coping mechanisms.

### Attachment styles in posttraumatic stress disorder

#### S. Leistner^1^, R. Mestel^2^ and R. Rosner^1^

^1^Institute für Clinical and Biological Psychology, Catholic University Eichstätt-Ingolstadt, Germany; ^2^HELIOS Clinic Bad Groenenbach, Germany

*Objectives*: Previous studies have shown insecure attachment as a risk factor for mental disorders. Furthermore, research has uncovered attachment styles as moderators between critical incidents and the occurrence of PTSD. However, there is little information whether patients with PTSD differ in their attachment patterns from patients with other mental disorders and healthy controls. *Method*: Therefore, we compared patients with PTSD (*n=*2666), patients with other mental disorders (*n=*11110) and students as healthy controls (*n=*84). Attachment style was assessed by the Relationship Questionnaire (RQ-2). Chi-square tests and ANOVAs were applied for estimating group differences. *Results*: Results demonstrate that 64.3% of controls may be classified as having a secure pattern of attachment while the majority of patients with PTSD as well as patients with other mental disorders developed a fearful-avoidant (39.9%/30.3%) or preoccupied (28.9%/27.0%) attachment style. There were statistically significant effects for the secure (p<0.001, η^2^=0.01) and the fearful-avoidant (p<0.001, η^2^=0.02) attachment patterns between the three groups: Patients with PTSD showed more rarely a secure but more often a fearful-avoidant attachment style compared to patients with other mental disorders and healthy controls, too. While only 35.7% of healthy controls had insecure attachment patterns, 81.5% of the patients with other mental disorders and 87.5% of the persons with PTSD belonged to the group of insecure-attached persons. *Discussion*: Results stress the importance to give attention to attachment patterns and their possible consequences in working with psychosomatic patients, particularly in presence of PTSD. Limitations of the study are the small sample size of healthy controls as well as measuring attachment styles by a self-report instrument.

### Psychometric evaluation of the Grief Questionnaire for children and adolescents

#### P. Fornaro, J. Unterhitzenberger and R. Rosner Katholische Universität, Psychologie, Eichstätt-Ingolstadt, Germany

Complicated Grief (CG) is discussed to be added as a new diagnosis in DSM-V and ICD-11. Therefore, the need for evaluated inventories on CG will be high. For adults, e.g., the Inventory of Complicated Grief (ICG) is well disseminated and evaluated. However, concerning children and adolescents, there is hardly any psychometric evaluation reported for grief instruments. We investigated the psychometric properties of the Grief Questionnaire for Children and Adolescents (CG-CA) which was first used in a study with adolescents in Rwanda. The CG-CA consists of 36 items, which were mainly extracted from the Extended Grief Inventory (EGI) and supplemented with grief-related trauma items. 69 Adolescents (52% male) aged 14 to 18 years (*M*=16.3, *SD=*1.16) completed the CG-CA at two measurement points and provided data for the evaluation. An exploratory factor analysis revealed the existence of two factors. The questionnaire showed a high internal consistency (*α=*0.94). Furthermore, the CG-CA showed good concurrent and construct validity. The effect size for a correlation with impairment of daily functioning was high. A cut-off for an indicated CG-treatment was computed with a sensitivity of 85.3% and a specificity of 85.9%. This suggests evidence for the test's high predictive validity. The findings indicate that the CG-CA is a suitable questionnaire for assessing CG. Nevertheless the examination of its psychometric properties took place in a small orphaned sample in a third-world country setting. To increase external validity, it needs to be evaluated in a general population sample and—as criteria for CG are mainly based on research in western countries—evaluation should take place in this region as well.

### The traumatic real beyond the dream: the repetition in the symptomatic phenomena related to trauma

#### F. D'Antonio Dipartimento di Scienze dell'Uomo, Universitá degli Studi di Urbino “Carlo Bo”, Urbino, Italy

Symbolic and real define two aspects in opposition of subjective experience; they offer a clinical perspective to the reading of trauma and trauma-related symptomatic phenomena. The symbolic order coincides with the “laws of language” that structure the unconscious, whereas the real order is opposed to the symbolic; it is the “unknown,” anxiety and drive, and concerns the “unassimilable” part of the trauma. A traumatic event is a “real” experience that is “beyond the functioning of the unconscious” and beyond the laws that structure the dream formation, as theorized by Freud and transposed into linguistics by Jacques Lacan. The trauma breaks the defensive power of the symbolic order and creates a fixation on the real of the body. This fixation implies a real repetition of the traumatic event that is persistently identical with itself. Repetition, beyond the power of representation of the dream, is the generating principle of symptomatic phenomena, such as nightmares and flashbacks, which characterize the tendency to relive the traumatic event compulsorily within the posttraumatic disorders. Anxiety is the signal of the encounter with real: the phenomenon that reveals the irruption of the “traumatic real” beyond the protective shield of the symbolic. Within this theoretical and clinical perspective, “put in to words the events,” as a process of symbolization and attribution of meaning to the “real” traumatic experience, becomes the principle that guides a possible therapeutic intervention on the traumatized subject.

References

American Psichiatric Association. (2000). *Manuale diagnostico e statistico dei disturbi mentali – Text revision (DSM-IV-TR)*. Tr. it. Masson, Milano 2002.

Lacan, J. (1964). *Il Seminario, Libro XI, I quattro concetti fondamentali della psicoanalisi*. Tr. it. Einaudi, Torino 2003.

Recalcati, M. (2012). *Jacques Lacan. Desiderio, godimento e soggettivazione*. Raffaello Cortina, Milano.

## The Spectrum of Trauma-Related Disorders

### Trauma and identity: a narrative study among refugee torture survivors

#### Adrienn Kroó Department of Psychology, University of Pécs, Pécs, Hungary; Cordelia Foundation for the Rehabilitation of Torture Victims, Budapest, Hungary

The classical concept of PTSD has been criticized for many reasons, including its cultural limitations and negligence of long-lasting changes to personality, which has been documented in cases of chronic interpersonal trauma. The concept of complex PTSD has addressed these shortcomings and specifies “alterations in self-perception” as one of the characteristic changes in functioning following extreme and chronic trauma. Torture survivors and refugees are a population particularly affected by cumulative traumatization. The experience of torture comprises the deliberate and systematic devastation of values, beliefs, self-concept, and personality development. It has severe effects on the survivors’ fundamental trust, self-identity, and attachment. Meanwhile, refugee trauma involves the long-term experience of discrimination, persecution, helplessness, and humiliation, which also has a significant impact on the individual's personal and collective identity. A number of studies and theories now exist, which describe the psychological effect of torture and refugee trauma on identity. However, the issue of torture and life in exile is generally discussed separately. The present research aims at integrating the two phenomena and focuses on the unique and combined effect that torture and refugee trauma has on the survivors’ identity. The qualitative study comprises narrative in-depth interviews with refugee torture survivors and applies a psychoanalytic framework for interpreting the data.

## Effects of Trauma on Families and Children

### Individual differences in mothers’ response to their infant's affective states: a functional MRI case study and meta-analysis

#### J. Thome^1^, P. A. Frewen^2^, M. Densmore^2^, A. Schore^3^ and R. Lanius^2^

^1^Department of Psychiatry, University of Western Ontario & Central Institute of Mental Health, Medical Faculty Mannheim, Heidelberg University, Germany; ^2^Department of Psychiatry, University of Western Ontario, Canada; ^3^University of California at Los Angeles, USA

We investigated using behavioral coding and functional neuroimaging of a series of four mothers, three with childhood trauma histories, two of whom had current posttraumatic stress disorder (PTSD). Behavioral measures were obtained while mothers interacted with their infants. Subsequently, mothers viewed videos of their own child as well as a standard infant in each of three affective states (happy, neutral, and sad). Behavioral findings indicated, with regard to their maternal sensitivity scores, that mothers who suffered from PTSD showed a more healthy maternal behavior (0.61 [case 1] and 0.59 [case 2]) as compared to mothers with no psychopathology (0.41 [case 3] and 0.37 [case 4]). Interestingly, supporting those findings, neuroimaging results for mothers with childhood trauma histories showed greater brain activation during exposure to one's own infant relative to an unfamiliar infant in regions associated with social cognition (e.g., fusiform gyrus, precuneus) and emotion-empathy (e.g., anterior cingulate cortex, insula). In contrast, the mother without childhood trauma history exhibited a lack of response in brain regions associated with social cognition and emotion-empathy. In conclusion, the findings in traumatized mothers with PTSD provide some evidence for these mothers’ ability to break the cycle of intergenerational transmission of trauma. We also performed a voxel-based whole-brain meta-analysis of functional neuroimaging studies investigating the neural correlates of healthy mothers’ attachment experiences by examining brain response while mothers viewed pictures, videos, or heard sounds of their infants. Results indicated a comparable neuronal response pattern observed in three mothers with a childhood trauma history described above, thus providing further evidence for the capacity of women with a history of critical life events for resilience.

### Perceived anxiety of family among firefighters: a comparison of Korean and Japanese

#### S. Yoo^1^, Y. Matsui^1^, M. Hatanaka^2^, H. Joo^3^, C. Park^3^, M. Han^3^ and H. Ahn^3^

^1^Institute of Psychology, University of Tsukuba, Japan; ^2^Faculty of Human Studies, Meijo University, Japan; ^3^Ewha Womans University, Korea

Perceived anxiety of family among Japanese and Korean firefighters was investigated. 535 Japanese firefighters dispatched to the disaster areas severely affected by the Great East Japan Earthquake participated in the study following 3–4 months after the earthquake (N=511, who gave valid responses to the questionnaire). 1,507 Korean firefighters were requested to answer one of the two questionnaires (A: traumatic stress, B: general mental health) via e-mail, the valid responses to the questionnaire were 533 (N_A_=267, N_B_=266). Results of Korean firefighters (N=266) were compared with Japanese in terms of perception of family's anxiety, 9 items about whether one has felt the anxiety of one's family. First of all, of the participants, only 23.4% of Japanese and 22.6% of Korean participants responded affirmatively to the item, “There were no family that felt stress, or anxiety about my dispatch,” suggesting that three-quarters of participants in both country believed that their families had experienced anxiety related to their rescue work. With regard to the difference between two countries, affirmative rate of Japanese was higher than Korean in following items. (1) My family felt anxiety because of the frightful spectacle of the disaster area in media coverage (χ^2^(1)=57.902, *p*<0.001). (2) My family felt anxiety because they didn't know about my activities in the disaster area (χ^2^(1)=5.245, *p*<0.05). In following items, the affirmative rate of Korean was higher. (1) My family felt anxiety because my appearance was changed by stress (χ^2^(1)=22.321, *p*<0.001). (2) My family felt anxiety because they did not know how to relieve my stress (χ^2^(1)=28.026, *p*<0.001). These findings indicate the need to provide mental health care to family members of firefighters when conducting interventions for firefighters.

### Corporal punishment in childhood and subsequent physical health and mental health risk and outcomes in early adulthood: the moderating effects of parental warmth and consistency

#### P. Petretic, K. Burleson, M. Calvert, M. Karlsson and J. Henrie
University of Arkansas, Fayetteville, AR, USA

This study examined the relation between experience of corporal punishment in childhood and later health outcomes as measured by number of physical illnesses, health risk behaviors, psychological risk, and lack of health-promoting behaviors in young adulthood. It has been suggested that physical abuse and physical discipline exist on a continuum, such that they are quantitatively, not qualitatively, different. Research supports a link between child physical abuse and numerous negative outcomes, including physical health sequelae (e.g., Felitti, 1998). Considering that corporal punishment is used by the majority of American parents, it is important to examine if this parenting practice is associated with comparable developmental outcomes. Thus, the current study investigated if corporal punishment might have a similar, albeit less severe, impact on later health outcome as physical abuse. Research has further suggested that family environment can affect the relation between corporal punishment and outcome, with parental warmth and consistency moderating the relation between parenting practices and subsequent outcomes. We further examined the moderating effect of parental warmth and consistency to determine if harsh parenting has a less detrimental impact within the context of a warm and consistent environment. In this sample of 188 young college adults, corporal punishment did not predict physical illnesses, risk behaviors (including activities related to substance use and sexual behaviors), psychological risk (including symptoms and diagnoses of mental illness, sleep problems, life difficulties, and disabilities), or health-promoting behaviors (including routine health maintenance activities) when controlling for age and sex. However, the relation between corporal punishment and number of physical illnesses was significantly moderated by parental consistency. In addition, parental warmth was found to be a significant, unique predictor for risk behaviors and psychological risk, with higher levels of warmth related to lower levels of risk behaviors and psychological risk.

### Self-blame and PTSD in adolescents surviving terrorism: the mediating role of school connectedness

#### U. Moscardino^1^, S. Scrimin^1^, F. Capello^1^ and G. Altoè^2^

^1^Department of Developmental and Social Psychology, University of Padova, Padova, Italy; ^2^Department of Pedagogy, Psychology, Philosophy, University of Cagliari, Cagliari, Italy

Researchers agree that coping strategies are key determinants of youth psychological adjustment following terrorism (Pfefferbaum, Noffsinger, & Wind, 2012). In particular, self-blame related to survivor guilt has been shown to increase the risk of posttraumatic stress disorder (PTSD) in adolescents (Drury & Williams, 2012). School connectedness, defined as students’ perceptions of being accepted by the school and identifying themselves as being part of the school, is strongly associated with positive psychological outcomes (e.g., Resnick et al., 1997). However, the role of school connectedness in the relationship between self-blame and adolescent PTSD after terrorist activities remains unexplored. The aim of this small-scale, cross-sectional study is to examine whether school connectedness mediates the link between self-blame and PTSD in adolescents who survived the 2004 terrorist attack against school no. 1 in Beslan, Russia. Sixty adolescents (aged 14–18 years) directly and indirectly exposed to the attack completed measures of coping, school connectedness, and PTSD three years after the traumatic event. More than half of adolescents (N=41, 68.3%) met full criteria for PTSD. No associations emerged between age, gender, exposure, and diagnosis of PTSD. We found a relationship between self-blame and diagnosis of PTSD (OR=1.88, 95% CI=1.12, 3.16). We also found a relationship between self-blame and school connectedness (B=−0.26, SE=0.06, p<0.05). Mediation analysis indicated that, after adjusting for relevant covariates, school connectedness partially mediated the relationship between self-blame and presence of PTSD, with an OR reduction of 23%. Findings suggest that adolescent survivors of terrorist attacks may benefit from school-based interventions aimed at teaching proactive coping skills as well as supporting students’ sense of belonging and emotional bonding to teachers, peers, and the school environment.

### Intimate partner violence victimization and perpetration: the predictive role of attachment and other risk factors in young adult females

#### P. Petretic, J. Henrie, M. Karlsson and M. Calvert
University of Arkansas, Fayetteville, AR, USA

Attachment style has been hypothesized as a mediating variable which may predict differential outcome in causal models of intimate partner violence (IPV) (Lettieri, 1996). Perpetrators and victims of IPV are more likely to have insecure attachment types when compared with individuals in non-violent relationships (Goldenson et al., 2007). This study examines the predictive role of adult attachment styles in relation to IPV perpetration and victimization to determine if attachment insecurity is a unique predictor of victimization or perpetration when child abuse experiences, witnessing interparental abuse, and adult cognitive distortions are incorporated in the causal model. Female college students (*N*=189) completed the following measures: The Revised Conflict Tactics Scale (IPV), Childhood Maltreatment Interview Schedule Short Form (childhood maltreatment and witnessing parental IPV), Experiences in Close Relationships Revised (adult anxious- and avoidant-attachment), and the Cognitive Distortions Scale (negative cognitions). In this relatively high-functioning sample, preliminary regression analyses revealed that anxious attachment predicted psychological abuse perpetration [*F*(1, 149)=9.075, *p*=0.003] and psychological abuse victimization [*F*(1, 146)=13.493, *p*<0.001]. Anxious attachment and cognitive distortions of self-blame were correlated [*r*(178)=0.517, *p*<0.001], and both emerged as unique predictors depending on the order of entry of the variables within hierarchical regression analyses. These analyses indicated childhood abuse, as well as anxious attachment and self-blame, are important pathways to adult IPV in our sample of young adult females. While childhood abuse appears to be an important distal predictor of IPV victimization and perpetration, adult anxious attachment and self-blame serve as more proximal predictors. The common thread between these latter variables is a negative self-evaluation in relational functioning. Our findings suggest that childhood maltreatment experiences set in motion a cognitive framework that predicts later trauma, such as IPV.

### Posttraumatic stress disorder symptoms in the first weeks following the preterm infant's hospital discharge

#### N. Goutaudier^1^, E. Bui^2^, N. Séjourné^1^ and H. Chabrol^1^

^1^Octogone – Centre d’études et de recherches en psychopathologie, Université de Toulouse II – le Mirail, Toulouse, France; ^2^Department of Psychiatry, Massachusetts General Hospital & Harvard Medical School, Boston, Massachusetts, USA

*Background*: Although over 5% of women develop clinically significant posttraumatic stress disorder (PTSD) symptoms directly related to their experience of giving birth, few data are available regarding prevalence and features associated with PTSD symptoms following preterm birth. This study aims to examine features associated with PTSD symptoms following the preterm birth. *Method*: Within 4 weeks of the infant's hospital discharge [mean (SD) time since discharge=2.2 (1.0) weeks), 110 French women (mean (SD) age=29.5 (4.3) years] who delivered prematurely [mean (SD) time since delivery=14.5 (3.5) weeks] completed the Impact of Event Scale-Revised (IES-R, range 0–88) and the Edinburgh Postnatal Depression Scale (EPDS, range 0–30), the Multidimensional Scale of Perceived Social Support and the Dyadic Adjustment Scale. Demographic and clinical data and information related to traumatic event exposure were also collected. *Results*: Mean (SD) IES-R and EPDS scores were 25.24 (18.31) and 22.19 (6.79), respectively and 30% of mothers (n=33) scored above the cut-off for probable PTSD. IES-R score correlated with depressive symptoms (*r*=0.42, *p*<0.05), C-section delivery (*r=*0.22, *p*<0.05), prior traumatic exposure, (*r=*0.21, *p*<0.05), and gynecological history (*r=*0.20, *p*<0.05) but not with perception of partner's support and quality of marital relationship (all *p*s > 0.10). Multivariate analyses revealed that increased postpartum depressive symptoms (*ß=*0.45, *p*<0.05), having undergone a c-section (*ß=*0.23, *p*<0.05), traumatic event exposure in the 12 months prior to childbirth (*ß=*0.19, *p*<0.05), were independently associated with PTSD symptoms, and explained 28.0% of the variance in PTSD symptoms. *Conclusion*: PTSD symptoms were independently associated with increased depressive symptoms, c-section and prior traumatic exposure, suggesting that these factors might be involved in the development or maintenance of PTSD symptoms after preterm delivery. Future longitudinal studies examining the long-term impact of premature birth are warranted.

### Rates and predictors of posttraumatic stress disorder of children and adolescents in foster care

#### E. Rimane^1^, E. Groh^2^, J. Arnold^2^, M. Hagl^2^ and R. Rosner^1^

^1^Kathollische Universität, Psychologie, Eichstaett/Ingolstadt, Germany; ^2^Ludwig-Maximilians-University, Munich, Germany

*Background*: Causes for children to be placed in foster families are very often connected with psychotrauma. Regarding these background, it is surprising that there exists comparably only little research about posttraumatic stress disorder (PTSD) in foster children. Furthermore, a comparison between the results of international studies is complicated as the foster care systems in different countries vary considerably. The aim of this study is to examine the rate of PTSD in a sample of German foster children. Possible risk factors for the development of PTSD in foster children are analyzed. *Methods*: Seventy-four foster children (10–18 years old) and their foster parents were studied using a wide range of diagnostic instruments. Among these were the Child Behavior Checklist, the Child Dissociative Checklist, the Childhood Trauma Questionnaire, and a detailed questionnaire to explore family relationships. PTSD was assessed using the German version of the Clinician-Administered PTSD Scale for Children and Adolescents (CAPS-CA). *Results*: Five percent of the foster children fulfilled a PTSD diagnosis according to DSM-IV criteria, 22% regarding to ICD-10 criteria. Significant correlations between the severity of PTSD and some risk factors were found. These include the sum score of the Childhood Trauma Questionnaire (CTQ), the age of entering the foster family, and the reasons for the outplacement. Entering these three predictors in a regression model, only the CTQ sum score remained significant. *Discussion*: Compared to other internationally published foster children studies, the rate of PTSD is quite small. Possibly this might be due to the recruitment conditions, leading to an oversampling of healthy children. As there were only few PTSD cases, it is not surprising that most of the assumed predictors remained insignificant. Nevertheless, the CTQ seems to be a good predictor for PTSD in foster children.

### Resilient emotional competence in pediatric diabetes

#### G. Regini Dipartimento di Scienze dell'Uomo, Università degli studi di Urbino “Carlo Bo”, Urbino, Italy

Diabetes is a chronic disease whose abrupt onset triggers traumatic experiences and requires a psychological adjustment to the patients and their family. Achieving this adaptation is a necessary goal for the proper control of the disease. On the contrary, patients may conflict with it, exposing them at high risk of psycho-physical complications. The ability to favour bio-psycho-behavioural adjustments, post-traumatic, allows the remodelling of the internal state of the child, promotes, and activates resilience, i.e. the capacity of the Self to self-organize. This process is facilitated by the integration of a sense of Self by state transitions ensuring continuity of experience and inner cohesion, as well as the emotional regulation and attachment experiences. According to Schore's model, situations of attachment influence the development of the right hemisphere, dominant for processing, expressing and regulating the emotional information. The two components (sympathetic and parasympathetic) of the ANS not only regulate automatic and somatic aspects of emotional states but also of the stress response, so the attachment relationship is able to directly model the maturation of systems of stress management that act on an unconscious level in the brain of the child. Affective experiences regulated (and unregulated) are stored in the orbitofrontal system in its cortical and sub-cortical connections, these internal interpersonal representations fulfil the role of biological regulators that control the mental processes, allowing the development of homeostasis, but also the maturation of the orbitofrontal cortex itself, so of self-regulatory and stress recovery mechanisms. In chronic disease, activation of recovery through emotional re-channelling represents an important protective factor both with respect to the possibility of future re-traumatisation and to facilitate the adaptation.

References

ISPAD Clinical Practice Consensus Guidelines Compendium (2009): Psychological care of children and adolescents with diabetes. *Pediatric Diabetes 10*(12), 175–184.

Schore A. N. (2003b). *I Disturbi del Sé. La disregolazione degli affetti*. Astrolabio Editore, Roma, 2010.

Schore A. N. (2003a). *La regolazione degli affetti e la riparazione del Sé*. Astrolabio Editore, Roma, 2008.

### Combat trauma and intimate partner relationships: a review and analysis

#### M. Weavers and C. Piotrowski Department of Family Social Sciences, University of Manitoba, Winnipeg, Canada

Posttraumatic stress disorder (PTSD) symptoms have been consistently linked to a range of negative family functioning outcomes. Combat veterans with PTSD have a higher likelihood of multiple divorces, verbal and physical aggression, sexual dysfunction, impairments in emotional expressiveness, and emotional numbing symptoms associated with relationship dissatisfaction (Monson, Taft, & Fredman, 2009). Recent work has supported the notion that trauma not only affects the primary victim but also those to whom they are intimately connected. However, there has been lack of attention given to the course of combat trauma and couple distress—specifically the mechanisms by which symptoms and distress are maintained or exacerbated. This review addresses this gap in the literature by providing a critical review of empirical work on the interaction between combat trauma and intimate relationships. In addition, theoretical perspectives that attempt to explain mechanisms of how trauma influences family functioning, including caregivers burden, ambiguous loss, reintegration, secondary traumatization, couple adaptation to traumatic stress model, and cognitive-behavioral interpersonal model are critically reviewed. The need for a bidirectional causal framework is emphasized; however, limitations of these perspectives necessitate further research. To this end, Conservation of Resources Theory (Hobfoll, 1988, 1989) is presented as a promising framework for the investigation of the cyclical intersection of combat trauma symptomology and couple distress, using the avoidance cluster symptoms as a specific example. Recommendations for future research utilizing this framework are outlined.

Reference

Monson, C. M., Taft, C. T., & Fredman, S. J. (2009). Military-related PTSD and intimate relationships: From description to theory-driven research and intervention development. *Clinical Psychology Review, 29*, 707–714.

### Does age at trauma exposure matter in the development of motivational abilities?

#### K. Simmen-Janevska, A. Horn, S. Krammer and A. Maercker Department of Psychology, Psychopathology and Clinical Intervention, University of Zurich, Zurich, Switzerland

*Objectives*: Exposure to traumatic stress may have a negative impact on subsequent motivational development. This study examined the relationship between childhood adversities and motivational abilities in late adulthood depending on developmental psychological stages. *Methods*: The motivational abilities self-efficacy, conscientiousness, and impulsivity (self-control) were investigated in a sample of 114 former Swiss indentured child laborers (so-called “Verdingkinder”) with a mean age of 77.6 years. These individuals were separated from their biological families early in life and were placed mostly in farmer families where they were forced to work to earn their own living. Potentially traumatic events during childhood were assessed by using the Childhood Trauma Questionnaire (CTQ). The sample was split in four age groups according to the beginning of the trauma: infancy (0–2), preschool (3–5), early childhood (6–9), and early adolescence (≥10). *Results*: 81.6% of the participants reported clinically relevant CTQ values, with emotional neglect being the most prominent childhood adversity. Age group comparisons did not reveal significant differences with regard to the three motivational variables. The strongest relationship was found between self-efficacy and CTQ total score (*r=*−0.59, *p*<0.01) in the group early adolescence, followed by the relationship between conscientiousness and CTQ total score (*r=*−0.39, *p*<0.05) in the same age group. Finally, impulsivity and CTQ total score were most strongly associated in preschoolers (*r=*0.37, *p*<0.05). *Conclusion*: Trauma factors seem to have a negative impact on self-efficacy and conscientiousness after the age of 10. In contrast, impulsivity (self-control) seems to be affected by the deleterious effect of trauma already at an earlier age. This study is the first in trying to answer the question, whether traumatic stress in childhood negatively influences the development of motivational abilities over the life span, which is of ultimate importance in dealing with life requirements.

### Exploring the support networks of breast cancer survivors

#### P. Nirgude, N. Hunt and S. Thomas Institute of Work, Health & Organisations, University of Nottingham, UK

*Background*: The importance of social support in chronic illnesses like breast cancer has been well-documented. However, currently missing from the literature is an in-depth exploration of the support networks of breast cancer survivors. *Aim*: This study aimed to investigate the support networks of breast cancer survivors with a view to understanding the type of support (e.g., emotional, practical, medical) that was provided by sources and the way in which these sources are linked and the extent to which they support each other. *Methods*: Using a qualitative, narrative approach, 10 breast cancer survivors were interviewed about their breast cancer journey. In addition to the interviews, ecomaps were developed to provide a framework which depicted the strength of relationships and direction of support between members of the support network and the breast cancer survivor. *Findings*: Thematic analysis revealed two core themes: gender stereotyping in support and treatment side-effects on relationships. The first theme illustrates how male partners tended to provide practical support, whereas female friends provided emotional support. The second theme relates to the debilitating effect of treatment and how this left the breast cancer survivor unable to socialise. The impact and relevance of these themes are diagrammatically represented using examples from the ecomaps. Associations between the sources of support are also represented. *Discussion*: This study highlights the effect of gender differences and treatment side-effects on the support networks of breast cancer survivors. Through ecomapping, the complexity of the support network can be represented, which shows that there does not seem to be a main provider of support to the breast cancer survivor, but instead there are a number of sources which provide different types of support. This research gives insight into the complexity and availability of support to breast cancer survivors.

## Impact of Trauma on Communities

### Transition between past and present: surrendering inverse rules of history through peace and reconciliation efforts with a new generation in Bosnia-Herzegovina

#### J. White
The Chicago School of Professional Psychology, Chicago, IL, USA

Bosnia is shaped like a human heart in Southeastern Europe. The unique pre-war ethnic composition of Bosnia included Muslims or Bosniaks, Serbians or Catholics, and Croatians or Orthodox Christian. Genocide in Bosnia attempted to ethnically cleanse unarmed Muslims from their communities. The cold war, the longest siege in modern history represented a tragedy in Bosnia-Herzegovina. Children war victims are now young adults and have faced intergenerational transmission of pathogenic emotions. The universal existence of intergenerational trauma has had a lasting effect. The cold war has shaped lives of victimized children and the same images will continue to shape the lives of generations to come. Communal wounds are the reality of Bosnia-Herzegovina. In turn, such anti-human behavior may create a disconnect with memory. The product of intractable ethnic hatreds cannot be a simplistic explanation when recalling violent memories. The further the past recedes, the closer it becomes. With a qualitative design, Bosnian young adults will be asked to remember wrongs suffered, which represents an unbearable crime against humanity. Reconciliation efforts have become the forerunner in post-conflict peace building. A process toward sustainable peace includes changing destructive behavior patterns between former enemies into constructive relationships to further empower Bosnian young adults for communal reconciliation.

### Posttraumatic stress reactions in middle-age non-clinical sample and effects of social transformations in a country

#### N. Grigutyte, I. Vaskeliene, D. Gailiene and E. Kazlauskas Vilnius University, Vilnius, Lithuania

*Background*: In 20th century, Lithuania underwent two World Wars, Nazi and Soviet occupations—the last one lasted for 50 years. These social–political transformations included forced integration into Soviet Union, political repressions, and constraints in all the country. The research question is what kind of effect this has on current mental health in the general population. *Methods and participants*: We analysed a non-clinical sample of middle-aged participants. The sample was divided into two groups: one of those participants, whose mother or father survived Soviet or Nazi political repression, and the others, who were matched according to socio-demographic characteristics and whose parents did not directly experience political repressions. The participants completed the questionnaire which assessed their life-time trauma experiences, present posttraumatic stress reactions, and subjective consequences of parents’ political repression to their life. In continuing study, participants from the general population were asked about their attitudes towards the social transformations in the country to analyse the consequences of political repression to broader population. *Results*: The results show that two non-clinical samples of middle-aged participants did not differ in PTSD reactions, but parents’ experiences of political repression were considered as having affected their life and psychological well-being. So in a broader sample, effects of social transformations were analysed and the results show the importance of these historical events in the country.

## Evidence-Based Practice on Trauma

### Psychotherapeutic interventions from the western world in war-traumatized children—a meta-analysis

#### A. Nocon, S. Von Jan and R. Rosner Katholische Universität Eichstaett-Ingolstadt, Eichstaett, Germany

*Background*: There has been lately some effort in the treatment of traumatized child and adolescent post-conflict populations, and a growing body of evidence shows that psychotherapyis effective in this group. However, with treatments usually being designed and applied in industrialized countries, little is known whether the treatment context has any impact on its outcome. A bibliography and meta-analysis were used to examine interventions for children and adolescents that were applied in industrialized countries vs. those applied in war-torn countries of origin. *Methods*: A literature search produced 21 studies covering 14 different kinds of psychotherapeutic interventions; of these, only 9 both (1) were randomized and (2) reported pre- and post-intervention scores. Five studies investigated the effects of psychotherapy in refugees seeking asylum in western countries, 2 investigated the effects of psychotherapy in refugees seeking asylum in countries with similar to the original culture, and 14 examined interventions in displaced youth in their country of origin. *Results*: Both cognitive behavior therapies (CBT) and psychodynamic interventions were effective for trauma symptoms. The methodological quality of the retrieved studies, however, was very diverse. Most treatment studies for refugees in western countries did not use a control group. The only randomized controlled trial (RCT) applied to refugees in the United States reported no differences between play therapy and trauma-focused CBT. RCTs applied to refugees in their country of origin/similar cultureyielded Cohen's d between 0.27 and 1.80. Non-RCT effects were between 0.61and 1.31 for treatment in western countries, and between 0.03 and 0.91 in the original/neighbor country. *Limitations*: Limitations included methodological inconsistencies across studies and lack of a randomized control group design, yielding few studies for meta-analysis. *Conclusions*: The superiority of a specific intervention might change with the treatment context. Further research is needed to identify the most effective treatment in a specific context.

### Moderators of intervention outcomes among children disaster survivors: a meta-analysis

#### N. Kirlic^1^, R. Tett^1^, S. Nelson^1^, B. Liles^1^, E. Newman^1^ and B. Pfefferbaum^2^

^1^Tulsa Institute of Trauma, Abuse and Neglect and Department of Psychology, The University of Tulsa, USA; ^2^College of Medicine, The University of Oklahoma Health Sciences Center, USA

Post-disaster environments pose a unique set of mental health delivery challenges, requiring intervention delivery readily deployable and maximally effective. Our knowledge is limited with respect to the impact such challenges have on intervention outcomes among children survivors of disasters presenting with post-traumatic symptoms. We used meta-analysis to assess whether interventions vary in efficacy across intervention types, settings, and levels of professional training. Thirty-three studies were identified that provided outcome data on interventions for children exposed to natural and man-made disasters, wars, accidents, and other sudden traumatic events. Interventions were carried out in school and health or mental health settings, and intervention providers included mental health professionals and teachers and other school professionals. Large effect sizes were found for interventions in reducing PTSD symptoms, and intervention conditions resulted in better outcomes than control conditions. Outcomes varied by the type of intervention, but not by the setting in which the intervention was carried out or by the providers’ training level. Generally, exposure therapies yielded the largest and psychological debriefing/crisis the smallest effect sizes. Children receiving interventions in schools did not differ from children in health or mental health settings. Mental health professionals and teachers and other school professionals had similar success when delivering interventions for children survivors of disasters. Our results suggest that special attention be paid to the type of intervention utilized to reduce PTSD symptoms in this population, but that schools and teachers can serve as appropriate resources for effective intervention delivery.

### Evaluating a multidisciplinary public approach for treating victims of rape and sexual assault in Denmark

#### L. H. Nielsen and A. Elklit Department of Psychology, National Center for Psychotraumatology, University of Southern Denmark, Denmark

In Denmark, around 500 rapes are reported to the police every year and it is estimated that around three to four times more are actually committed. International research has established that rape is an extremely traumatic event that can have long-term negative consequences for victims including psychological, sexual, behavioral, and physical problems. Rape traumas do not exist in a cultural and societal vacuum. Hence, experiences with the legal, medical, and mental health system following a rape can profoundly affect victims’ well-being following an assault—both in a positive and negative way (Campbell et al., 1999). In Denmark, the first multidisciplinary public approach for treating victims of rape and sexual assault was established in 1999 (Bramsen, Elklit & Nielsen, 2009). This approach has not yet been evaluated, so we do not know whether we are inadvertently hurting the victims we are trying to help and how this might affect them. The aim of the current Ph.D. project is to evaluate how victims of rape and sexual assault in Denmark experience the help they receive through the multidisciplinary public system and how they perceive their interactions with the different professionals they meet in this system (police officers, nurses, medical examiners, psychologists, and attorneys among others). The aim of the project is to evaluate: (1) Does the multidisciplinary public approach meet the needs of victims of rape and sexual assault when they approach the system for help? (2) Does the system unintentionally re-victimize the victims they are trying to help and in what way does this affect the psychologically well-being of the victims following a sexual assault? Evaluation data is collected in the acute phase following the rape and at follow-up six months post-assault and this is combined with psychological data already collected at the rape crisis center at the same intervals.

### Spanish-validated tests in paediatric psychological trauma assessment

#### A. M. Garcia-Sanchez^1^, J. M. Martin-Jimenez^2^ and R. A. Garcia-Oliva^3^

^1^Universidad de La Laguna, Tenerife, Spain; ^2^Servicio Canario de Salud, Las Palmas, Spain; ^3^Servicio Canario de Salud, Las Palmas, Universidad Nacional de Educacion A Distancia, Bogota, Colombia

Assessment trauma is, as much in children as in adults, the first step when planning the therapy. But, there is a lack of validated children assessment instruments in some trauma fields when a research is designed in Spanish. Some complex trauma symptoms as dissociation in paediatric population could not be measured by any validated questionnaire in our language. We think revising the most accurate assessment instruments in Spanish could be useful for Spanish-speaking researchers in children's trauma. In addition, revising also the main fields related to complex trauma could be interesting to recall researchers and clinicians that it is important not to forget any of those fields to have a wide and exhaustive profile of the trauma impact in children.

### ”Actimeter” as an innovative tool for the objective measurement of sleep disorder of torture survivors with complex PTSD

#### U. H. Harlacher and L. Nordin DIGNITY—Danish Institute Against Torture, Copenhagen, Denmark

An “actimeter” is a watch-like device worn around the wrist, which continuously measures and stores (hand/arm) movements. The accumulated data over about one week deliver, besides other, sleep-related data that allow for the quantitative analysis of important parameters like total sleep duration, sleep-latency, and frequency of sleep interruptions. First experiences using this tool as a part of the interdisciplinary treatment of sleep problems at DIGNITY in Copenhagen, where torture survivors with complex PTSD and other complex problems are treated, are positive. Wearing the advice continuously during one week is well tolerated by most clients. The quantitative measurement seems to be reliable since there is a god correlation with the client‘s subjective description of physical activity during the day. Besides for measurement, the advice is also usable as a therapeutic tool since most clients become motivated and curious about to inspect and analyze the results since corrections of negative expectations, e.g., about the duration of the first sleep-phase, can be made and since it is easier to identify potential interventions, e.g., correction of timing of medication. The device will be presented, its use explained, and experiences made with the tool so far will be presented using case descriptions including actimeter-outcome graphs.

### Very brief exposure in PTSD—a pilot project on tortured and traumatised refugees

#### L. Nordin and U. H. Harlacher DIGNITY—Danish Institute Against Torture, Copenhagen, Denmark

Fear responses can be activated outside of awareness by masked phobic stimuli with a very brief stimulus onset (Öhman & Soares, 1994). Within experimental psychology research, it has long been known that very brief stimuli can trigger physiological responses, i.e., stimuli that do not lead to conscious perception may trigger a response. When an anxiety provoking image is shown on the computer screen so fast that it only appears as a flash of light, subjects respond by exhibiting a measurable physiological response corresponding to an anxiety response. Siegel and Weinberger (2009) have shown that very brief exposure (25 ms) to images of spiders promoted approach towards a live tarantula. This pilot trial is a modified replication on tortured and traumatised refugees suffering from posttraumatic stress disorder (PTSD). Additional to Siegel and Weinberger's (2009) experiment, physiological parameters will be measured with a non-invasive 64-channel electroencephalograph, heart rate, and electric skin conductance. The objective is to evaluate whether very brief evoked responses can also be observed with PTSD-related stimuli in traumatised refugees and whether repeated very brief exposure will result in decreasing avoidance of trauma stimuli. The experiment and experiences made will be presented, using case-descriptions including data from the physiological parameters.

References

Siegel, P., & Weinberger, J. (2009). Very brief exposure: The effects of unreportable stimuli on fearful behavior. *Consciousness and Cognition, 18*, 939–951.

Öhman, A., & Soares, J. J. F. (1994). “Unconscious Anxiety”: Phobic Responses to Masked Stimuli. *Journal of Abnormal Psychology, 103*, 231–240.

